# Giese-type alkylation of dehydroalanine derivatives via silane-mediated alkyl bromide activation

**DOI:** 10.3762/bjoc.20.271

**Published:** 2024-12-17

**Authors:** Perry van der Heide, Michele Retini, Fabiola Fanini, Giovanni Piersanti, Francesco Secci, Daniele Mazzarella, Timothy Noël, Alberto Luridiana

**Affiliations:** 1 Department of Chemical and Geological Sciences, University of Cagliari, S.S. 554, bivio per Sestu, 09042 Monserrato (CA), Italy; 2 Flow Chemistry Group, Van ’t Hoff Institute for Molecular Sciences (HIMS), University of Amsterdam Science Park 904, 1098 XH Amsterdam, The Netherlands; 3 Department of Biomolecular Sciences, University of Urbino ‘‘Carlo Bo”, Piazza Rinascimento 6, 61029 Urbino, Italy; 4 Department of Chemical Sciences and Technologies, University of Rome “Tor Vergata” Via della Ricerca Scientifica, 1, 00133 Rome, Italy; 5 Department of Chemical Sciences, University of Padova Institution, Via Francesco Marzolo, 1, 35131 Padova, Italy

**Keywords:** dehydroalanine, Giese-type reaction, hydroalkylation, photocatalysis, water

## Abstract

The rising popularity of bioconjugate therapeutics has led to growing interest in late-stage functionalization (LSF) of peptide scaffolds. α,β-Unsaturated amino acids like dehydroalanine (Dha) derivatives have emerged as particularly useful structures, as the electron-deficient olefin moiety can engage in late-stage functionalization reactions, like a Giese-type reaction. Cheap and widely available building blocks like organohalides can be converted into alkyl radicals by means of photoinduced silane-mediated halogen-atom transfer (XAT) to offer a mild and straightforward methodology of alkylation. In this research, we present a metal-free strategy for the photochemical alkylation of dehydroalanine derivatives. Upon abstraction of a hydride from tris(trimethylsilyl)silane (TTMS) by an excited benzophenone derivative, the formed silane radical can undergo a XAT with an alkyl bromide to generate an alkyl radical. Consequently, the alkyl radical undergoes a Giese-type reaction with the Dha derivative, forming a new C(sp^3^)–C(sp^3^) bond. The reaction can be performed in a phosphate-buffered saline (PBS) solution and shows post-functionalization prospects through pathways involving classical peptide chemistry.

## Introduction

**Figure 1:**
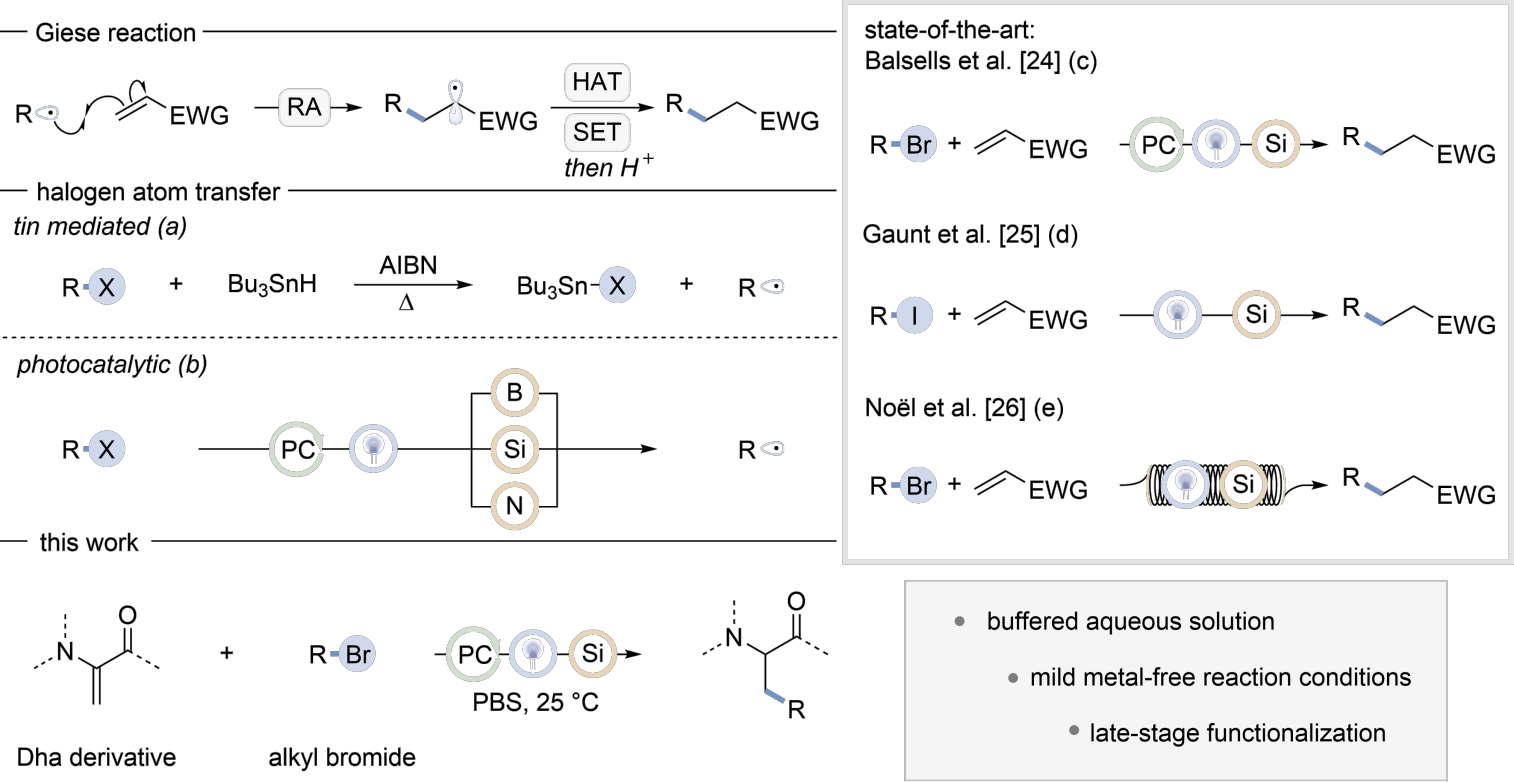
Giese reaction: Radical addition on olefins with an electron-withdrawing group (EWG) followed by a HAT or SET and protonation; halogen-atom transfer: (a) tin-mediated XAT, (b) XAT initiated by a photocatalyst (PC) and mediated by boranes (B), silanes (Si) or alkylamines (N); state-of-the-art: (c) silane-mediated alkylation initiated by a photocatalyst, (d) silane-mediated alkylation initiated by photolysis of alkyl iodides, (e) silane-mediated alkylation initiated by photolysis of alkyl bromides in flow; this work: silane-mediated alkylation of Dha derivatives initiated by a photocatalyst.

The construction of C(sp^3^)–C(sp^3^) bonds is a highly important target in synthetic organic chemistry. Historically, polar conjugate additions have been a benchmark method for constructing these bonds by functionalizing an electron-deficient olefin [1–3]. Recently, however, radical-based approaches have also gained widespread attention for their unique advantages in these transformations [4]. Radical chemistry often exhibits complementary reactivity to two-electron pathways and can be performed with high selectivity, atom economy, and functional group tolerance [5]. A well-known radical pathway for the functionalization of an electron-deficient olefin is the Giese reaction (Figure 1) [6–7]. This reaction involves the hydroalkylation of the olefin via radical addition (RA), followed by either hydrogen-atom transfer (HAT) or single-electron transfer (SET) and protonation.

Traditionally, alkyl radicals have been produced from alkyl halides, using azobisisobutyronitrile (AIBN) as initiator, promoting a tin-mediated XAT (Figure 1a) [8–9]. However, tin-based compounds are highly toxic and require harsh conditions for the initiation event.

Fortunately, a renaissance in the field of photochemistry has introduced new ways of generating radicals like photoredox catalysis and via electron donor–acceptor (EDA) complexes [10–13]. These advances, coupled with modern electrochemical methods, chemical reactor engineering and light emitting diodes (LED), have eliminated the need for thermal radical activation, resulting in milder and safer reaction conditions [14–16]. Given the toxicity of tin-based compounds, there has been significant interest in developing alternative halogen-atom-transfer reagents. Borane, alkylamine, and silane compounds have emerged as effective XAT reagents upon photocatalytic activation (Figure 1b) [17–21]. A photocatalytic HAT or SET generates the corresponding boryl, α-amino or silyl radical, which can abstract a halogen atom from alkyl halides to form the corresponding alkyl radical.

However, the use of TTMS as a XAT reagent had already been established by Chatgilialoglu et al. [22] under non-photoredox conditions, MacMillan et al. [23] sparked renewed interest in silanes as XAT reagents by generating a tris(trimethylsilyl)silyl radical through photoredox catalysis for arylation reactions [22–23]. In 2018, Balsells et al. [24] reported a similar strategy to generate alkyl radicals and explored the feasibility of a Giese-type reaction (Figure 1c). More recently, Gaunt et al. [25] showed that irradiation of alkyl iodides combined with TTMS leads to the formation of an alkyl radical, which can be used in a Giese-type reaction without the need of a photocatalyst (Figure 1d) [25]. Noël et al. [26] have further extended this approach to include alkyl bromides (Figure 1e) [26]. Despite the effectiveness of the photolysis, benzophenone derivatives have also been shown to enhance the productivity of silane-mediated conjugate additions, using alkyl halides [27].

Amid the growing popularity of biomolecular drug candidates, the late-stage modification of peptide scaffolds has gained significant importance [28]. A particularly interesting class of amino acids for late-stage diversification consists of dehydroamino acids (Dha). Dha derivatives have shown modification potential by means of polar, metal-, or organo-catalyzed and radical additions [29–36]. As such, Dha derivatives make an excellent candidate for exploring a photochemical Giese-type reaction [37]. To foster a physiological reaction environment, reactions can be conducted in aqueous solution, meeting important requirements with regard to bioorthogonal chemistry [38].

Considering previous research that demonstrated photochemical hydrogen atom abstraction by benzophenone derivatives from trialkylsilyl hydrides [27], as well as advances in alkyl radical formation using these hydrides, we sought to combine these findings. Herein, we report a photochemical alkylation methodology targeting the olefin moiety of Dha derivatives, conducted in an aqueous solution for the aforementioned bioorthogonal advantages.

## Results and Discussion

Inspired by previously conducted research concerning benzophenone hydrogen-atom transfer and silane-mediated activation of alkyl bromides to perform a photochemical Giese reaction, methyl 2-(1,3-dioxoisoindolin-2-yl)acrylate (**1**) and bromocyclohexane (**2**) were dissolved in CH_3_CN (0.1 M) together with a stoichiometric amount of tris(trimethylsilyl)silane and a substoichiometric amount of (4-methoxyphenyl)(4-(trifluoromethyl)phenyl)methanone (BP **l**) (Table 1) [26–27].

**Table 1:** Methyl 2-(1,3-dioxoisoindolin-2-yl)acrylate (**1**, 0.5 mmol), bromocyclohexane (**2**, 1.25 mmol), tris(trimethylsilyl)silane (0.55 mmol), (4-methoxyphenyl)(4-(trifluoromethyl)phenyl)methanone (0.1 mmol), PBS 0.2 M solution (2.5 mL), λ = 390 nm, 25 °C, overnight. The yield of **3** was calculated by ^1^H NMR with 1,1,2-trichloroethene as external standard.

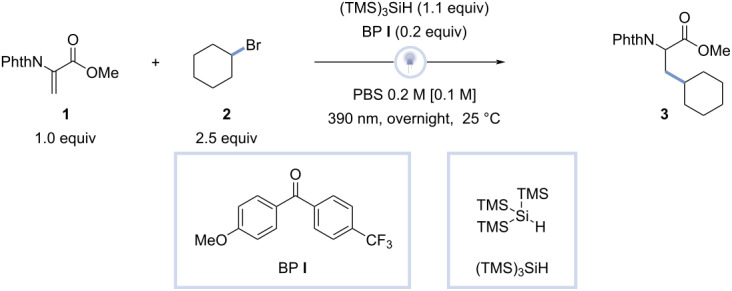			

Entry	Deviation from the reaction conditions	Conversion **1** (%)	NMR yield **3** (%)

1	CH_3_CN instead of PBS 0.2 M; 1.5 equiv (TMS)_3_SiH	85	51
2	CH_3_CN instead of PBS 0.2 M; no BP **l**; 1.5 equiv (TMS)_3_SiH	90	38
3	H_2_O instead of PBS 0.2 M; 1.5 equiv (TMS)_3_SiH	100	58
4	PBS 0.1 M instead of PBS 0.2 M	100	44
5	none	100	60
6	PBS 0.4 M instead of PBS 0.2 M	100	60
7	**3 hours**	**100**	**67**

The reaction was performed in batch, using a 390 nm Kessil UV-A lamp, stirring at 600 rpm overnight at 25 °C (see Supporting Information File 1 for a more detailed optimization).

**Figure 2:**
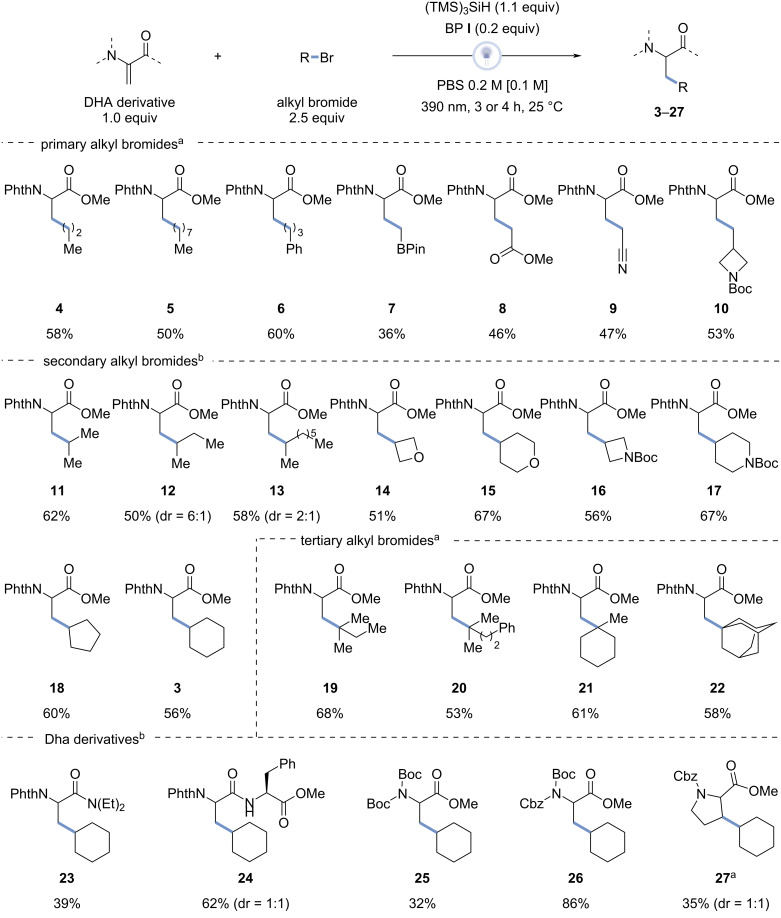
Alkyl bromide and Dha derivative scope. Reaction conditions: Dha derivative (0.5 mmol), alkyl bromide (1.25 mmol), tris(trimethylsilyl)silane (0.55 mmol), (4-methoxyphenyl)(4-(trifluoromethyl)phenyl)methanone (0.1 mmol), PBS 0.2 M solution (2.5 mL), λ = 390 nm, 25 °C. ^a^4 hours of reaction time, ^b^3 hours of reaction time. The isolated product yields are reported.

The initial reaction, using CH_3_CN as solvent, led to formation of methyl 3-cyclohexyl-2-(1,3-dioxoisoindolin-2-yl)propanoate (**3**, 51% yield, 85% conv.; Table 1, entry 1). To demonstrate the importance of the photocatalyst, BP **l** was excluded (Table 1, entry 2), resulting in a slightly higher conversion and a decrease in product formation (38% yield, 90% conv.), meaning BP **l** increases the productivity of the reaction. Having established that the reaction works in CH_3_CN, we evaluated its compatibility with water. To our delight, the reaction in water provided full conversion and a higher yield than the one observed in CH_3_CN (58% yield, 100% conv.; Table 1, entry 3). These conditions were further refined by adding of phosphate-buffered saline and decreasing the amount of (TMS)_3_SiH to 1.1 equiv, as no further increase in yield was noticed during the optimization. Similar to the reaction in deionized water, all entries with PBS solution reached a full conversion. While the yield of the reaction with a 0.1 M PBS solution was slightly lower than that of the reaction in deionized water (44% yield, 100% conv.; Table 1, entry 4), a 0.2 M PBS solution resulted in an increased yield (60% yield, 100% conv.; Table 1, entry 5). Upon increasing the concentration of the PBS solution to 0.4 M, no change in yield was observed (60% yield, 100% conv.; Table 1, entry 6). Lastly, the optimal reaction time was determined to be 3 hours (67% yield, 100% conv.; Table 1, entry 7). Having optimized the reaction conditions, a scope of primary, secondary, and tertiary alkyl bromides and different Dha derivatives was investigated (Figure 2).

A slight modification of the reaction protocol was used in regard to primary and tertiary alkyl bromides, requiring a longer reaction time (4 hours instead of 3 hours) to achieve full conversion.

The reaction worked well with aliphatic primary bromides (**4**–**10**). However, a longer chain length slightly decreased the yield, comparing compound **4** (58%) to compound **5** (50%). The reaction was not influenced by the presence of phenyl rings, as the yield of compound **6** (60%) was comparable to the yield of **4**. Besides, the introduction of a boronic pinacol ester group, useful for subsequent post-functionalization, compound **7** was achieved in a relatively lower yield of 36%. Interestingly, the reaction also worked adequately for primary alkyl bromides with electron-withdrawing groups as demonstrated in compounds **8** (46%) and **9** (47%). Moreover, primary alkyl bromides like bromomethylazetidines as used in compound **10** (53%) also resulted in decent yields. Acyclic secondary alkyl substrates all resulted in good yields (**11**–**13**, 50–62%). For the secondary alkyl substrates, a higher yield was notably obtained for compound **13** with a longer chain length than for **12**. Similarly, the secondary cyclic alkyl substrates used in compound **18** (60%) and **3** (56%) worked well for this reaction. Besides, oxygen-containing compounds **14** and **15** were obtained in yields of 51% and 67% and medicinally interesting groups like the azetidine in compound **16** (56%) and the piperidine in compound **17** (67%) also resulted in good yields. Concerning tertiary alkyl substrates, acyclic alkyl substrates used in compound **19** (68%) and **20** (53%) as well as cyclic alkyl substrates like bromomethylcyclohexane used in compound **21** (61%) and bromoadamantane in compound **22** (58%) were obtained in satisfactory yields.

Subsequently, different Dha derivatives were subjected to the optimized reaction conditions, using bromocyclohexane (**2**) as the alkyl bromide. In order to explore the effect of the presence of an amide moiety in the Dha derivatives, a tertiary amide was firstly used to exclude selectivity issues arising from the hydrogen atoms of the amide functionality, yielding compound **23** in synthetically useful yield (39%). Interestingly, a secondary amide was formed in a higher yield of compound **24** (62%) compared to a Dha with a tertiary amide on the same position. Alternatively, the use of a double Boc-protected Dha resulted in a rather low yield of compound **25** (32%), while varying one Boc-protecting group with a Cbz-protecting group increased the yield substantially to 86% for compound **26**. In addition, the use of a cyclic, *N*-Cbz-protected Michael acceptor, derived from proline, allowed for preparation of compound **27** with a 35% yield in 4 hours without control of diastereoselectivity (dr = 1:1).

**Figure 3:**
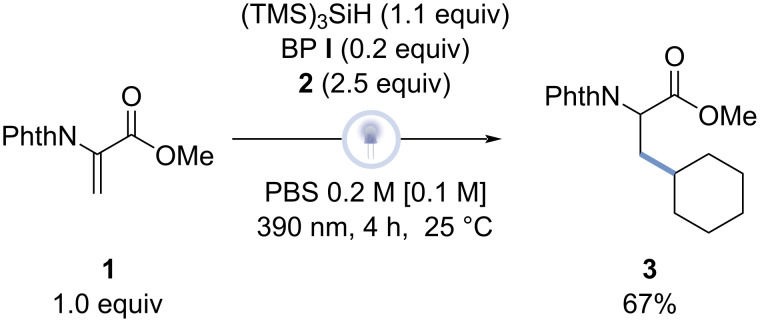
Scaled-up reaction. Reaction conditions: Dha derivative (2.2 mmol), alkyl bromide (5.4 mmol), tris(trimethylsilyl)silane (2.4 mmol), (4-methoxyphenyl)(4-(trifluoromethyl)phenyl)methanone (0.4 mmol), PBS 0.2 M solution (10.8 mL), λ = 390 nm, 25 °C, 4 hours, isolated product yield.

Finally, to prove that the optimized reaction also works at a larger scale, the model reaction was carried out on a 2.2 mmol scale (Figure 3), obtaining a slightly elevated yield (67%) compared to compound **3**, which was previously formed at a 0.5 mmol scale.

## Conclusion

In conclusion, a photochemical methodology to promote the metal-free alkylation of dehydroalanine derivatives was developed, by means of silane-mediated alkyl bromide activation. The biocompatibility of the reaction enabled by the PBS solution and the mild photochemical reaction conditions makes the transformation useful for late-stage functionalization under physiological conditions. Besides, the reaction was successfully scaled up by roughly four times, leading to a slight increase in chemical yield.

## Supporting Information

File 1 ^1^H NMR, ^13^C NMR, and HRMS spectra of all the synthesized compounds.

## Data Availability

All data that supports the findings of this study is available in the published article and/or the supporting information of this article.
