# Supplementary material for: Giese-type alkylation of dehydroalanine derivatives via silane-mediated alkyl bromide activation
Source: Beilstein J Org Chem. 2024 Dec 17;20:3274–80. doi: 10.3762/bjoc.20.271 (PMC11665442; doi:10.3762/bjoc.20.271)
Supplement: File 1 — 1H NMR, 13C NMR, and HRMS spectra of all the synthesized compounds. [file Beilstein_J_Org_Chem-20-3274-s001.pdf]

BEILSTEIN JOURNAL OF ORGANIC CHEMISTRY

Supporting Information

for

Giese-type alkylation of dehydroalanine derivatives via silane-mediated alkyl bromide activation

Perry van der Heide, Michele Retini, Fabiola Fanini, Giovanni Piersanti, Francesco Secci, Daniele Mazzarella, Timothy Noël and Alberto Luridiana

Beilstein J. Org. Chem. **2024,** *20,* 3274–3280. doi:10.3762/bjoc.20.271

^{1}H NMR, ^{13}C NMR, and HRMS spectra of all the synthesized compounds

License and Terms: This is a supporting information file under the terms of the Creative Commons Attribution License (https://creativecommons.org/licenses/by/4.0). Please note that the reuse, redistribution and reproduction in particular requires that the author(s) and source are credited and that individual graphics may be subject to special legal provisions.
The license is subject to the *Beilstein Journal of Organic Chemistry* terms and conditions: (https://www.beilstein-journals.org/bjoc/terms)

Table of contents

1. General information....S2
2. Reactor design....S3
    2.1. Reaction setup....S3
    2.2. Photocube reactor....S4
3. Reaction Optimization....S5
    3.1. Solvent screening....S5
    3.2. Solvent effects on deprotected N-Dha....S6
    3.3. Base screening....S6
    3.4. Photocatalyst screening....S7
4. General procedures....S8
    4.1. Giese-type reaction....S8
    4.2. Starting material preparation....S10
5. Characterization of compounds....S11
6. NMR spectra....S20
7. References....S46

1. General information

Materials. All used commercially available reagents and solvents were bought from Fluorochem, TCI, Merck and BLD Pharm and used without any further purification. Most of the substrates are commercially available. The dehydroalanine derivatives methyl 2-(1,3-dioxoisoindolin-2-yl)acrylate[1], methyl 2-(bis(*tert*-butoxycarbonyl)amino)acrylate[2], methyl 2-(((benzyloxy)carbonyl)(*tert*-butoxycarbonyl)amino)acrylate[2], methyl (2-(1,3-dioxoisoindolin-2-yl)acryloyl)-L-phenylalaninate[3], and 1-benzyl 2-methyl 4,5-dihydro-1*H*-pyrrole-1,2-dicarboxylate[4] were prepared following literary procedures. Alternative preparations are indicated in the starting material preparation section. Product isolation was performed using Merck 70–200 mesh silica gel. Thin-layer chromatography (TLC) was performed using 0.25 mm Aldrich silica gel 60-F plates combined with $KMnO_4$-staining for compound visualization.

NMR spectroscopy. Nuclear magnetic resonance (NMR) spectra were recorded using a Bruker Advance III HD 600 (600 MHz for ^{1}H NMR and 151 MHz for ^{13}C NMR) or Bruker Avance 400 spectrometer (400 MHz for ^{1}H NMR and 101 MHz for ^{13}C NMR). Deuterated chloroform was used as the solvent and trichloroethylene was used as the external standard ($CDCl_3$: 7.26 ppm for ^{1}H NMR and 77.16 ppm for ^{13}C NMR; TCE: 6.46 ppm for ^{1}H NMR). The chemical shift (δ) of the spectra is reported in parts per million (ppm) and the coupling constant (*J*) is given in hertz downfield from tetramethylsilane (TMS) as the internal reference compound. The multiplicities of the signals in the spectra are abbreviated accordingly: s (singlet), d (doublet), t (triplet), q (quartet), m (multiplet), dd (doublet of doublets), ddd (doublet of doublet of doublets), dt (doublet of triplets), td (triplet of doublets). NMR data were processed using MestReNova 14.2.0-26256.

Mass spectrometry. High-resolution mass spectrometry (HRMS) spectra were recorded on a Bruker High Resolution Mass Spectrometer in fast atom bombardment (FAB^+) ionization mode with CH_3CN as solvent or using a Q-TOF microTM mass spectrometer. HRMS data were processed using MestReNova 14.2.0-26256.

Diastereomeric ratio determination. The diastereomeric ratios were determined by integration of the concerned peaks in the ^{1}H NMR spectrum of the crude reaction mixtures.

2. Reactor design

2.1. Reaction setup

The reaction mixture was irradiated by a Kessil PR160L 390 nm lamp at a distance of 13 cm, stirring the solution at 600 rpm. The reactions were performed in a 20 mL vial with a septum including cap and a 25 mm diameter. A LLG-magnetic stirrer RCT standard 2.0 stirring plate was used in combination with a 20 mm PTFE stirring bar to stir the reaction mixture. The reaction setup is demonstrated with the lamp turned off (a) and on (b) (Figure S1).

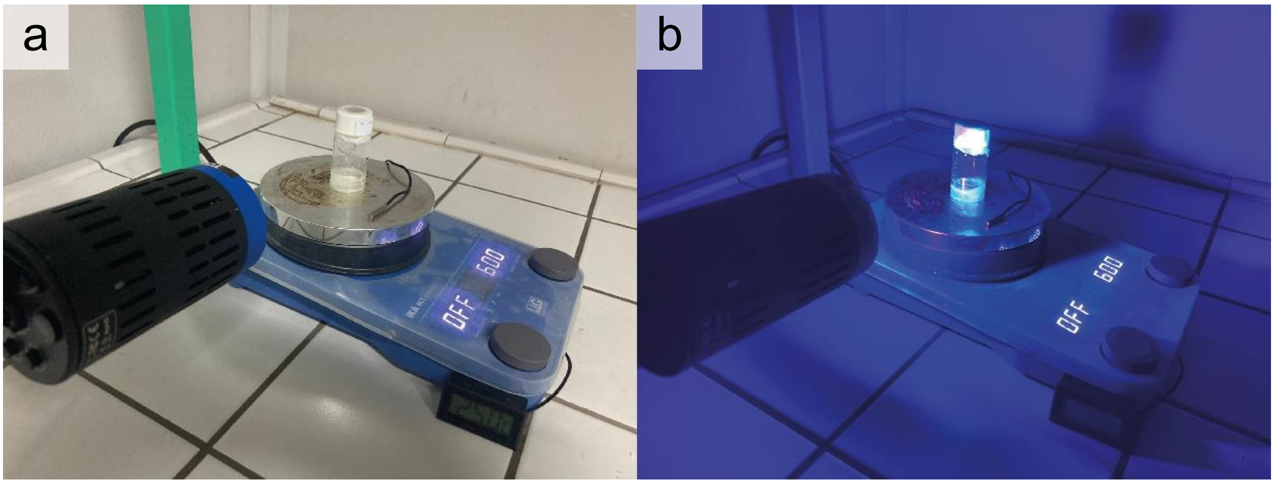

Figure S1. Overview of the reaction setup with the lamp turned off (a) and on (b).

2.2. Photocube reactor

A thalesnano photocube was used to conduct the reactions, using a 20 mL vial with a septum including cap and a 25 mm diameter (Figure S2). Light at a wavelength of 395 nm was used to irradiate the mixture, stirring the reaction at 405 rpm with a 20 mm PTFE stirring bar.

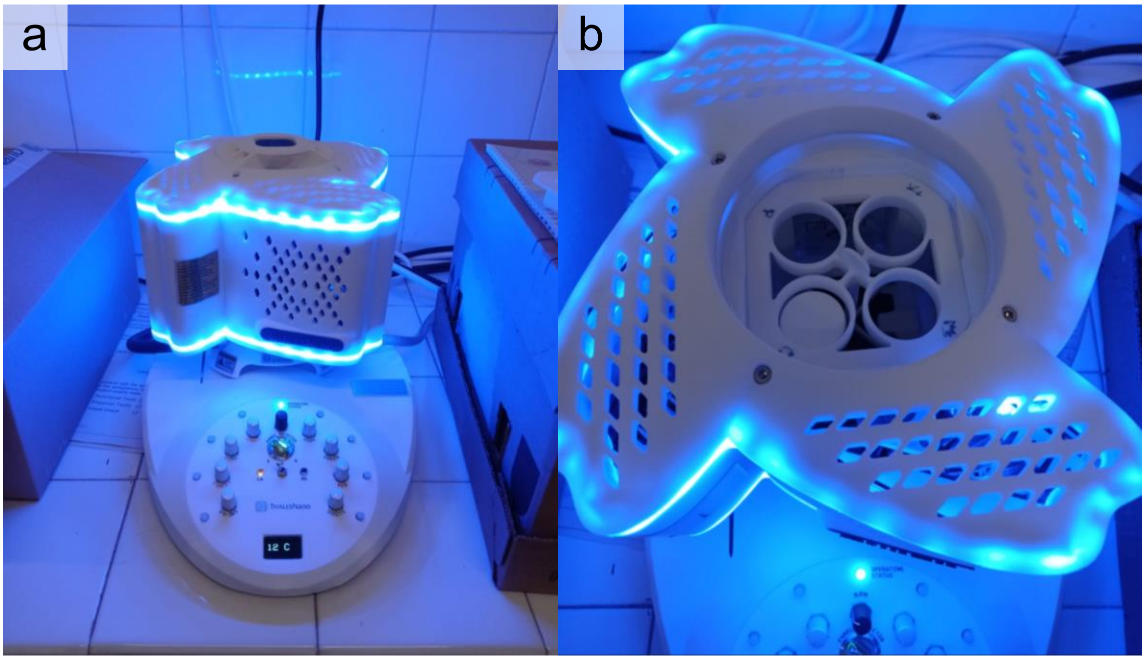

Figure S2. Overview of the photocube setup (a, from the side; b, from the top).

3. Reaction optimization

In order to perform the optimization studies, the reactor and the lamps were evaluated first. A large vial and high stirring were fundamental to have homogeneous dispersion of the reaction mixture. For this reason, the reaction was performed in a 20 mL vial with vigorous stirring (600 rpm). Besides, the result was determined by the intensity of the light source. Using a 390 nm Kessil lamp (100% intensity), a distance of 13.0 cm from the lamp proved to give optimal results. A similar result was found using the thalesnano photocube (395 nm, 405 rpm).

3.1. Solvent screening

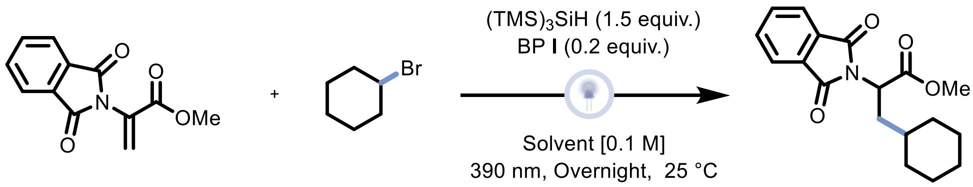

1 1.0 equiv. **2** 2.5 equiv. **3**

Entry	Solvent	Deviation	Conversion **1** (%)	Yield **3** (%)
1	CH_3CN	-	85	51
2	CH_3CN (No PC)	-	90	38
3	Acetone	-	100	69
4	EtOAc	-	100	55
5	MeOH	-	100	65
6	DMF	-	100	28
7	*t*-BuOH	-	100	65
8	EtOH	-	100	53
9	H_2O	-	100	58
10	H_2O:MeOH (7:3)	-	100	46
11	H_2O:EtOH (7:3)	-	100	46
12	H_2O:*t*-BuOH (7:3)	-	100	43
13	0.1 M PBS	1.1 equiv. $(TMS)_3SiH$	100	44
14	0.2 M PBS	1.1 equiv. $(TMS)_3SiH$	100	60
15	0.4 M PBS	1.1 equiv. $(TMS)_3SiH$	100	60
16	0.2 M PBS:MeOH (7:3)	-	100	18
17	0.2 M PBS:EtOH (7:3)	-	100	39
18	0.2 M PBS:*t*-BuOH (7:3)	-	100	47
19	0.2 M PBS:MeOH (9:1)	1.1 equiv. $(TMS)_3SiH$	100	38
20	0.2 M PBS:*t*-BuOH (9:1)	1.1 equiv. $(TMS)_3SiH$	100	23
21	0.2 M PBS	1.1 equiv. $(TMS)_3SiH$, 3 hours	100	67

Table S1. Solvent screening.

3.2. Solvent effects on deprotected N-Dha

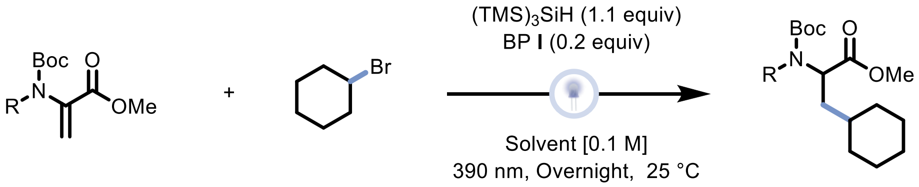

Dha 1.0 equiv. **2** 2.5 equiv. **25/26**

Entry	R	Solvent	Conversion **Dha** (%)	Yield **25/26** (%)
1	Boc	Acetone	Traces	None
2	Boc	MeOH	Traces	None
3	Boc	*t*-BuOH	Traces	22
4	Boc	H_2O	100	80
5	Boc	0.4 M PBS solution	100	53
6	Cbz	*t*-BuOH	100	41
7	Cbz	H_2O	100	53
8	Cbz	0.4 M PBS solution	100	60

Table S2. Solvent effects on deprotected N-Dha.

3.3. Base screening

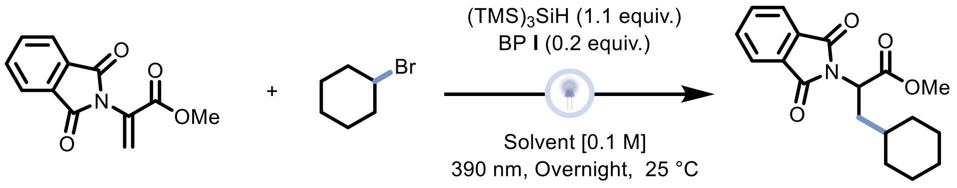

1 1.0 equiv. **2** 2.5 equiv. **3**

Entry	Solvent	Base	Conversion **1** (%)	Yield **3** (%)
1	0.2 M PBS solution	-	100	60
2	H_2O	K_3PO_4	100	31
3	H_2O	$NaHCO_3$	100	51
4	H_2O	Na_2CO_2	100	23
5	H_2O	Ce_2CO_3	100	19
6	H_2O	K_2CO_3	100	22

Table S3. Base screening.

3.4. Photocatalyst screening

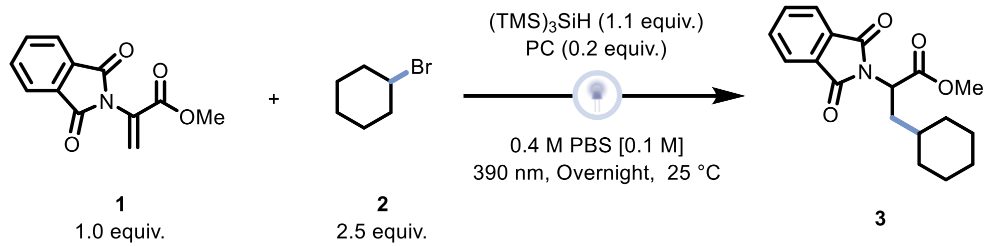

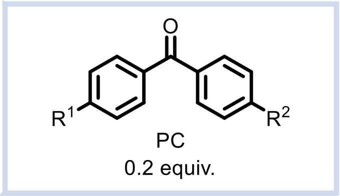

Entry	R^1	R^2	Conversion **1** (%)	Yield **3** (%)
1 (BP **I**)	$-CF_3$	-OMe	100	60
2	-H	-H	100	52
3	- (no PC)	- (no PC)	100	38

Table S4. Photocatalyst screening.

4. General procedures

4.1. Giese-type reaction

General procedure A

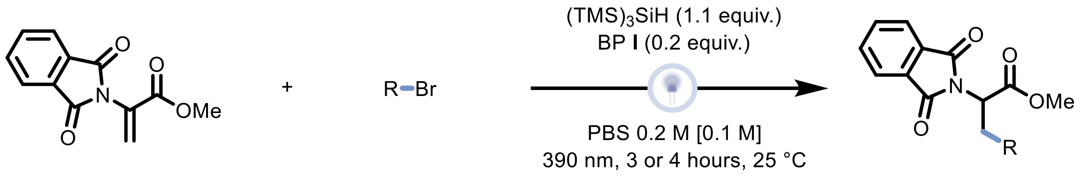

1 1.0 equiv. | **Alkyl bromides** 2.5 equiv. | **3-22**

Dha derivative **1** (116.0 mg, 0.50 mmol, 1.0 equiv) and BP **I** (28.0 mg, 0.10 mmol, 0.2 equiv) were added to an oven-dried vial, after which the vial was put under an argon atmosphere. The 0.2 M PBS solution (2.5 mL, 0.1 M), alkyl bromide (1.25 mmol, 2.5 equiv) and $(TMS)_3SiH$ (170 µL, 0.55 mmol, 1.1 equiv) were added to the vial. The mixture was bubbled with argon gas at 0 °C. The mixture was irradiated at a distance of 13 cm from a 390 nm PR160L Kessil lamp, while the reaction was stirred at 600 rpm and at room temperature (3 h for secondary bromides, 4 h for primary and tertiary alkyl bromides). After the reaction, deionized water (5.0 mL) was added into the vial, after which the reaction mixture was extracted three times with ethyl acetate (5.0 mL). The mixture was dried using sodium sulfate and concentrated under reduced pressure, resulting in compounds **3**–**22**.

General procedure B

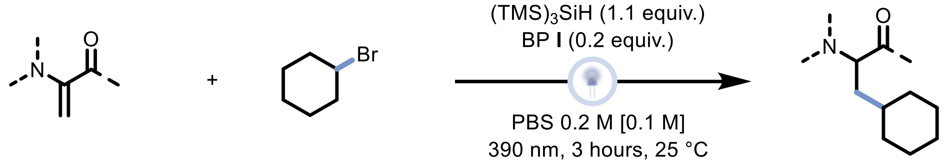

Dha derivatives 1.0 equiv. | **2** 2.5 equiv. | **23-27**

The Dha derivative (0.50 mmol, 1.0 equiv) and BP **I** (28.0 mg, 0.10 mmol, 0.2 equiv) were added to an oven-dried vial, after which the vial was put under an argon atmosphere. The 0.2 M PBS solution (2.5 mL, 0.1 M), bromocyclohexane (**2**, 154 µL, 1.25 mmol, 2.5 equiv) and $(TMS)_3SiH$ (170 µL, 0.55 mmol, 1.1 equiv) were added to the vial. The mixture was bubbled with argon gas at 0 °C. The mixture was irradiated at a distance of 13 cm from a 390 nm PR160L Kessil lamp, while the reaction was stirred at 600 rpm for 3 h at room temperature. After the reaction, deionized water (5.0 mL) was added into the vial, after which the reaction mixture was extracted three times with ethyl acetate (5.0 mL). The mixture was dried using sodium sulfate and concentrated under reduced pressure, resulting in compounds **23**–**27**.

General procedure for the scaled-up reaction

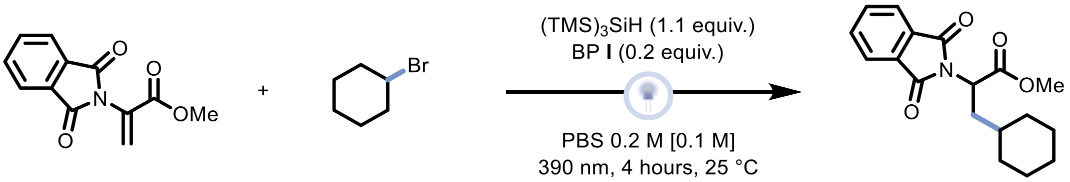

1 1.0 equiv. **2** 2.5 equiv. **3**

Dha derivative **1** (500.0 mg, 2.16 mmol, 1.0 equiv) and BP **I** (121.0 mg, 0.43 mmol, 0.2 equiv) were added to an oven-dried vial, after which the vial was put under an argon atmosphere. The 0.2 M PBS solution (10.8 mL, 0.1 M), bromocyclohexane (**2**, 668 µL, 5.41 mmol, 2.5 equiv) and $(TMS)_3SiH$ (734 µL, 2.38 mmol, 1.1 equiv) were added to the vial. The mixture was bubbled with argon gas at 0 °C. The mixture was irradiated at a distance of 13 cm from a 390 nm PR160L Kessil lamp, while the reaction was stirred at 600 rpm for 4 h at room temperature. After the reaction, deionized water (22.0 mL) was added into the vial, after which the reaction mixture was extracted three times with ethyl acetate (22.0 mL). The mixture was dried using sodium sulfate and concentrated under reduced pressure, resulting in compound **3** (456.0 mg, 67% yield).

4.2. Starting material preparation

Procedure for the synthesis of 2-(1,3-dioxoisoindolin-2-yl)-*N*,*N*-diethylacrylamide

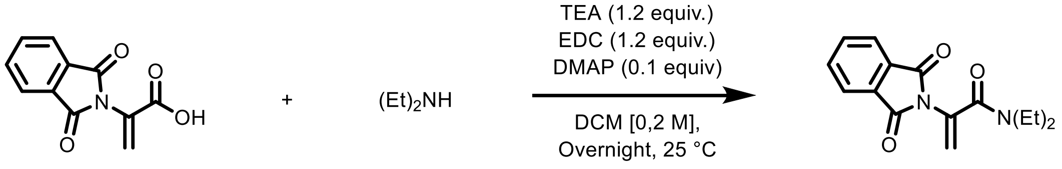

In an oven-dried round-bottomed flask, 2-(1,3-dioxoisoindolin-2-yl)acrylic acid (450 mg, 2.07 mmol, 1.0 equiv), triethylamine (347 µL, 2.49 mmol, 1.2 equiv), 3-(((ethylimino)methylene)amino)-*N*,*N*-dimethylpropan-1-amine hydrochloride (477 mg, 2.49 mmol, 1.2 equiv) and 4-dimethylaminopyridine (25.3 mg, 0.21 mmol, 0.1 equiv) were dissolved in dichloromethane (10.4 mL, 0.2 M).[5] After having stirred the reaction for 5 min, diethyl amine (214 µL, 2.07 mmol, 1.0 equiv) was added dropwise. The reaction was stirred overnight at room temperature. The reaction mixture was diluted with dichloromethane and quenched with deionized water. The mixture was washed with saturated sodium bicarbonate solution, saturated ammonium chloride solution, deionized water, and brine. The organic layer was dried with sodium sulfate and concentrated under reduced pressure. The crude mixture was purified by flash column chromatography (100% petroleum ether to petroleum ether 50:50 EtOAc) to afford 2-(1,3-dioxoisoindolin-2-yl)-*N*,*N*-diethylacrylamide (177.7 mg, 32% yield) as a pale yellow oil. ^{1}H NMR (600 MHz, $CDCl_3$) δ 7.87 – 7.79 (m, 2H), 7.75 – 7.69 (m, 2H), 5.85 – 5.78 (m, 1H), 5.52 – 5.42 (m, 1H), 3.63 – 3.37 (m, 4H), 1.21 – 1.15 (m, 6H). ^{13}C NMR (151 MHz, $CDCl_3$) δ 166.38, 165.78, 134.56, 131.96, 131.68, 123.78, 112.92, 43.32, 39.40, 13.71, 12.09. HRMS (ESI) *m/z* calcd for $C_{15}H_{17}N_2O_3$ $[M+H]^+$: 273.1239, found 273.1266.

Preparation of 0.2 M PBS solution

One PBS tablet is dissolved in 10 mL of deionized water, yielding a 0.2 M phosphate concentration, 0.054 M potassium chloride concentration and 2.74 M sodium chloride concentration with a pH of 7.4 at room temperature.[6] Upon preparing 10 mL of 0.2 M PBS, 1 tablet is crushed and transferred to a 10 mL volumetric flask, after which deionized water (10 mL) is added.

5. Characterization of compounds

Methyl 3-cyclohexyl-2-(1,3-dioxoisoindolin-2-yl)propanoate (3)

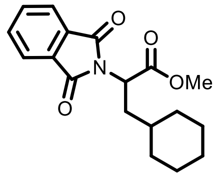

Prepared according to general procedure A, using bromocyclohexane (154 μL, 1.25 mmol, 2.5 equiv). The crude mixture was purified by flash column chromatography (100% petroleum ether to petroleum ether 90:10 EtOAc) to afford product **3** (88.6 mg, 56% yield) as a pale yellow oil. ^{1}H NMR (400 MHz, $CDCl_3$) δ 7.87 (dd, *J* = 5.5, 3.0 Hz, 2H), 7.74 (dd, *J* = 5.4, 3.3 Hz, 2H), 4.97 (dd, *J* = 11.4, 4.4 Hz, 1H), 3.71 (s, 3H), 2.31 – 2.21 (m, 1H), 2.08 – 1.97 (m, 1H), 1.92 – 1.82 (m, 1H), 1.72 – 1.55 (m, 4H), 1.21 – 1.07 (m, 4H), 1.04 – 0.96 (m, 1H), 0.96 – 0.87 (m, 1H). ^{13}C NMR (101 MHz, $CDCl_3$) δ 169.53, 166.88, 133.28, 131.00, 122.68, 51.83, 49.08, 35.06, 33.46, 32.82, 30.83, 25.51, 25.25, 25.00. The spectroscopic data are consistent with the data reported in literature.[7] HRMS (ESI) m/z calcd for $C_{18}H_{22}NO_4$ $[M+H]^+$: 316.1543; found 316.1548.

Methyl 2-(1,3-dioxoisoindolin-2-yl)hexanoate (4)

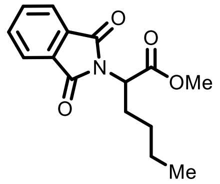

Prepared according to general procedure A, using 1-bromopropane (114 μL, 1.25 mmol, 2.5 equiv). The crude mixture was purified by flash column chromatography (100% cyclohexane to cyclohexane 90:10 EtOAc) to afford product **4** (80.2 mg, 58% yield) as a pale yellow oil. ^{1}H NMR (400 MHz, $CDCl_3$) δ 7.86 (dd, *J* = 5.5, 3.1 Hz, 2H), 7.74 (dd, *J* = 5.5, 3.6 Hz, 2H), 4.83 (dd, *J* = 10.4, 5.4 Hz, 1H), 3.72 (s, 3H), 2.33 – 2.16 (m, 2H), 1.42 – 1.19 (m, 4H), 0.85 (t, *J* = 7.0 Hz, 3H). ^{13}C NMR (101 MHz, $CDCl_3$) δ 170.09, 167.82, 134.28, 131.95, 123.65, 52.78, 52.28, 28.54, 28.49, 22.14, 13.95. HRMS (ESI) m/z calcd for $C_{15}H_{18}NO_4$ $[M+H]^+$: 276.1230; found 276.1228. The spectroscopic data are consistent with the data reported in literature.[8]

Methyl 2-(1,3-dioxoisoindolin-2-yl)undecanoate (5)

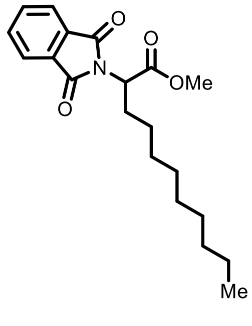

Prepared according to general procedure A, using 1-bromooctane (218 μL, 1.25 mmol, 2.5 equiv). The crude mixture was purified by flash column chromatography (100% cyclohexane to cyclohexane 90:10 EtOAc) to afford product **5** (86.7 mg, 50% yield) as a pale yellow oil. ^{1}H NMR (400 MHz, $CDCl_3$) δ 7.86 (dd, *J* = 5.4, 3.2 Hz, 2H), 7.74 (qt, *J* = 5.2, 3.1 Hz, 2H), 4.84 (dd, *J* = 10.6, 5.2 Hz, 1H), 3.72 (s, 3H), 2.33 – 2.17 (m, 2H), 1.32 – 1.19 (m, 14H), 0.84 (d, *J* = 6.8 Hz, 3H). ^{13}C NMR (101 MHz, $CDCl_3$) δ 170.11, 167.83, 134.28, 131.96, 123.65, 52.79, 52.32, 31.94, 29.56, 29.45, 29.33, 29.01, 28.76, 26.43, 22.75, 14.19. HRMS (ESI) m/z calcd for $C_{20}H_{28}NO_4$ $[M+H]^+$: 346.2013; found 346.2019.

Methyl 2-(1,3-dioxoisoindolin-2-yl)-6-phenylhexanoate (6)

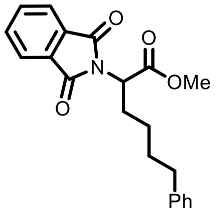

Prepared according to general procedure A, using (3-bromopropyl)benzene (191 µL, 1.25 mmol, 2.5 equiv). The crude mixture was purified by flash column chromatography (100% cyclohexane to cyclohexane 90:10 EtOAc) to afford product **6** (106.2 mg, 60% yield) as a pale yellow oil. ^{1}H NMR (400 MHz, $CDCl_3$) δ 7.87 (dd, *J* = 7.1, 3.5 Hz, 2H), 7.75 (dd, *J* = 5.5, 3.5 Hz, 2H), 7.24 – 7.18 (m, 2H), 7.15 – 7.09 (m, 3H), 4.86 (dd, *J* = 10.5, 5.2 Hz, 1H), 3.73 (s, 3H), 2.64 – 2.51 (m, 2H), 2.37 – 2.21 (m, 2H), 1.76 – 1.56 (m, 2H), 1.45 – 1.29 (m, 2H). ^{13}C NMR (101 MHz, $CDCl_3$) δ 169.99, 167.79, 142.24, 134.28, 131.90, 128.43, 128.33, 125.76, 123.65, 52.81, 52.14, 35.60, 30.70, 28.56, 25.93. HRMS (ESI) m/z calcd for $C_{21}H_{22}NO_4$ $[M+H]^+$: 352.1543; found 352.1549.

Methyl 2-(1,3-dioxoisoindolin-2-yl)-4-(4,4,5,5-tetramethyl-1,3,2-dioxaborolan-2-yl)butanoate (7)

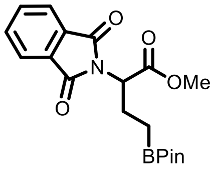

Prepared according to general procedure A, using bromomethylboronic acid pinacol ester (219 µL, 1.25 mmol, 2.5 equiv). The crude mixture was purified by flash column chromatography (100% petroleum ether to petroleum ether 85:15 EtOAc) to afford product **7** (63.9 mg, 36% yield) as a pale yellow oil. ^{1}H NMR (600 MHz, $CDCl_3$) δ 7.85 (dd, *J* = 5.3, 3.2 Hz, 2H), 7.72 (dd, *J* = 5.6, 3.3 Hz, 2H), 4.84 (dd, *J* = 10.9, 4.9 Hz, 1H), 3.72 (s, 3H), 2.41 – 2.26 (m, 2H), 1.19 (d, *J* = 9.7 Hz, 12H), 0.77 (dd, *J* = 8.8, 7.2 Hz, 2H). ^{13}C NMR (151 MHz, $CDCl_3$) δ 170.02, 167.86, 134.19, 132.09, 123.59, 83.40, 54.01, 52.72, 24.97, 24.84, 23.42. HRMS (ESI) *m/z* calcd for $C_{19}H_{25}BNO_6$ $[M+H]^+$: 374,1769 found 374,1784.

Dimethyl 2-(1,3-dioxoisoindolin-2-yl)pentanedioate (8)

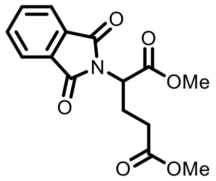

Prepared according to general procedure A, using methyl 2-bromoacetate (119 µL, 1.25 mmol, 2.5 equiv). The crude mixture was purified by flash column chromatography (100% hexane to hexane 70:30 EtOAc) to afford product **8** (71.0 mg, 46% yield) as a pale yellow oil. ^{1}H NMR (600 MHz, $CDCl_3$) δ 7.88 – 7.82 (dd, *J* = 5.5, 3.0 Hz, 2H), 7.76 – 7.70 (dd, *J* = 5.5, 3.0 Hz, 2H), 4.91 (dd, *J* = 10.4, 5.0 Hz, 1H), 3.72 (s, 3H), 3.59 (s, 3H), 2.61 (dtd, *J* = 14.4, 7.8, 5.0 Hz, 1H), 2.51 – 2.43 (m, 1H), 2.41 – 2.34 (m, 2H). ^{13}C NMR (151 MHz, $CDCl_3$) δ 172.69, 169.33, 167.60, 134.40, 131.81, 123.70, 52.91, 51.82, 51.24, 30.71, 24.36. The spectroscopic data are consistent with the data reported in literature.[9]

Methyl 4-cyano-2-(1,3-dioxoisoindolin-2-yl)butanoate (9)

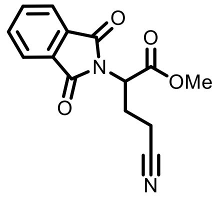

Prepared according to general procedure A, using 2-bromoacetonitrile (87 µL, 1.25 mmol, 2.5 equiv). The crude mixture was purified by flash column chromatography (100% petroleum ether to petroleum ether 70:30 EtOAc) to afford product **9** (63.8 mg, 47% yield) as a pale yellow oil. ^{1}H NMR (600 MHz, $CDCl_3$) δ 7.88 (dd, *J* = 5.5, 3.0 Hz, 2H), 7.76 (dd, *J* = 5.5 Hz, 3.0 Hz, 2H), 4.93 (dd, *J* = 9.5, 5.2 Hz, 1H), 3.74 (s, 3H), 2.66 – 2.59 (m, 1H), 2.54 (m, 1H), 2.50 – 2.45 (m, 2H). ^{13}C NMR (151 MHz, $CDCl_3$) δ 168.46, 167.49, 134.57, 131.62, 123.85, 118.34, 53.13, 50.67, 25.23, 14.69. HRMS (ESI) *m/z* calcd for $C_{14}H_{13}N_2O_4$ $[M+H]^+$: 273.0875, found 273.0870.

***tert*-Butyl 3-(3-(1,3-dioxoisoindolin-2-yl)-4-methoxy-4-oxobutyl)azetidine-1-carboxylate (10)**

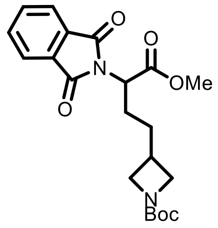

Prepared according to general procedure A, using *tert*-butyl 3-(bromomethyl)azetidine-1-carboxylate (314 mg, 1.25 mmol, 2.5 equiv). The crude mixture was purified by flash column chromatography (100% pentane to pentane 70:30 EtOAc) to afford product **10** (108.6 mg, 53% yield) as a pale yellow oil. ^{1}H NMR (600 MHz, $CDCl_3$) δ 7.83 (dd, *J* = 5.1, 2.5 Hz, 2H), 7.73 (dd, *J* = 5.5, 3.0 Hz, 2H), 4.78 (dd, *J* = 9.9, 5.7 Hz, 1H), 3.93 (dt, *J* = 11.1, 8.4 Hz, 2H), 3.68 (s, 3H), 3.49 – 3.41 (m, 2H), 2.46 (dtd, *J* = 13.5, 7.5, 3.6 Hz, 1H), 2.19 – 2.08 (m, 2H), 1.60 (ddt, *J* = 13.6, 10.3, 6.5 Hz, 1H), 1.50 (ddt, *J* = 14.1, 7.6, 2.6 Hz, 1H), 1.36 (s, 9H). ^{13}C NMR (151 MHz, $CDCl_3$) δ 169.53, 167.65, 156.34, 134.39, 131.72, 123.69, 79.27, 52.83, 51.87, 31.13, 28.40, 28.28, 26.35. HRMS (ESI) *m/z* calcd for $C_{21}H_{27}N_2O_6$ $[M+H]^+$: 403.1869, found 403.1861.

Methyl 2-(1,3-dioxoisoindolin-2-yl)-4-methylpentanoate (11)

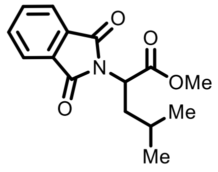

Prepared according to general procedure A, using 2-bromopropane (118 µL, 1.25 mmol, 2.5 equiv). The crude mixture was purified by flash column chromatography (100% cyclohexane to cyclohexane 90:10 EtOAc) to afford product **11** (90.1 mg, 62% yield) as a pale yellow oil. ^{1}H NMR (400 MHz, $CDCl_3$) δ 7.87 (dd, *J* = 5.6, 3.1 Hz, 2H), 7.74 (dd, *J* = 5.4, 3.0 Hz, 2H), 4.95 (dd, *J* = 11.6, 4.4 Hz, 1H), 3.73 (s, 3H), 2.34 (ddd, *J* = 14.3, 11.5, 4.1 Hz, 1H), 1.97 (ddd, *J* = 14.3, 10.2, 4.3 Hz, 1H), 1.54 – 1.42 (m, 1H), 0.94 (dd, *J* = 10.6, 6.6 Hz, 6H). ^{13}C NMR (101 MHz, $CDCl_3$) δ 170.40, 167.87, 134.29, 131.98, 123.65, 52.84, 50.75, 37.41, 25.19, 23.29, 21.15. The spectroscopic data are consistent with the data reported in literature.[10] HRMS (ESI) m/z calcd for $C_{15}H_{18}NO_4$ $[M+H]^+$: 276.1230; found 276.1223. The spectroscopic data are consistent with the data reported in literature.[11]

Methyl 2-(1,3-dioxoisoindolin-2-yl)-4-methylhexanoate (12)

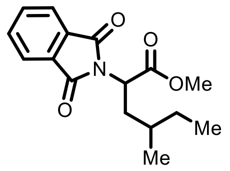

Prepared according to general procedure A, using 2-bromobutane (136 µL, 1.25 mmol, 2.5 equiv). The crude mixture was purified by flash column chromatography (100% cyclohexane to cyclohexane 90:10 EtOAc) to afford product **12** (76.1 mg, 50% yield) as a pale yellow oil (diastereoisomeric ratio = 6:1). ^{1}H NMR (400 MHz, $CDCl_3$) δ 7.87 (dd, *J* = 5.3, 3.4 Hz, 2H), 7.75 (dd, *J* = 5.5, 3.2 Hz, 2H), 4.96 (dd, *J* = 9.4, 3.4 Hz, 1H minor), 4.81 (dd, *J* = 7.7, 7.0 Hz, 1H major), 3.73 (s, 3H), 2.29 – 2.20 (m, 2H), 1.59 – 1.52 (m, 1H), 1.27 – 1.19 (m, 1H), 1.16 – 1.05 (m, 1H), 0.86 (t, *J* = 6.8 Hz, 6H). ^{13}C NMR (101 MHz, $CDCl_3$) δ 170.08, 167.83, 134.29, 131.94, 123.67, 52.80, 52.59, 35.48, 27.71, 26.78, 22.77, 22.32. HRMS (ESI) m/z calcd for $C_{16}H_{20}NO_4$ $[M+H]^+$: 290.1387; found 290.1379.

Methyl 2-(1,3-dioxoisoindolin-2-yl)-4-methyldecanoate (13)

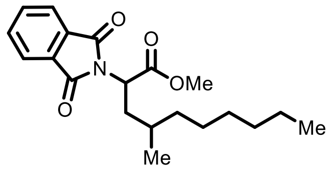

Prepared according to general procedure A, using 2-bromooctane (220 µL, 1.25 mmol, 2.5 equiv). The crude mixture was purified by flash column chromatography (100% cyclohexane to cyclohexane 90:10 EtOAc) to afford product **13** (105.0 mg, 58% yield) as a pale yellow oil (diastereoisomeric ratio = 2:1). ^{1}H NMR (400 MHz, $CDCl_3$) δ 7.86 (dd, *J* = 5.4, 3.1 Hz, 2H), 7.73 (dd, *J* = 5.5, 3.5 Hz, 2H), 5.00 – 4.92 (m, 1H), 3.72 (s, 3H), 2.45 (ddd, *J* = 14.6, 11.9, 2.8 Hz, 1H minor), 2.18 – 2.12 (m, 1H), 1.88 (ddd, *J* = 14.4, 10.3, 4.1 Hz, 1H major), 1.48 – 1.37 (m, 1H), 1.31 – 1.11 (m, 10H), 0.93 – 0.79 (m, 6H). ^{13}C NMR (101 MHz, $CDCl_3$) δ 170.50, 170.43, 167.88, 167.81, 134.27, 131.99, 123.64, 52.83, 52.82, 50.62, 50.54, 37.52, 36.00, 35.47, 35.21, 31.91, 31.89, 30.00, 29.85, 29.59, 29.57, 26.88, 26.48, 22.75, 22.71, 20.01, 18.73, 14.18, 14.16. HRMS (ESI) m/z calcd for $C_{20}H_{28}NO_4$ $[M+H]^+$: 346.2013; found 346.2018.

Methyl 2-(1,3-dioxoisoindolin-2-yl)-3-(oxetan-3-yl)propanoate (14)

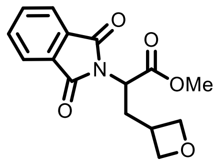

Prepared according to general procedure A, using 3-bromooxetane (104 µL, 1.25 mmol, 2.5 equiv). The crude mixture was purified by flash column chromatography (100% petroleum ether to petroleum ether 70:30 EtOAc) to afford product **14** (74.2 mg, 51% yield) as a pale yellow oil. ^{1}H NMR (600 MHz, $CDCl_3$) δ 7.84 (dd, *J* = 7.5, 3.7 Hz, 2H), 7.74 (dd, *J* = 5.5, 3.0 Hz, 2H), 4.76 – 4.68 (m, 2H), 4.62 (dd, *J* = 7.9, 5.9 Hz, 1H), 4.44 (t, *J* = 6.4 Hz, 1H), 4.31 (t, *J* = 6.2 Hz, 1H), 3.72 (s, 3H), 2.98 (tdd, *J* = 14.7, 8.4, 6.8 Hz, 1H), 2.63 (ddd, *J* = 13.9, 8.9, 4.9 Hz, 1H), 2.53 (ddd, *J* = 14.3, 10.6, 6.7 Hz, 1H). ^{13}C NMR (151 MHz, $CDCl_3$) δ 169.27, 167.48, 134.49, 131.66, 123.75, 76.89, 76.69, 52.95, 50.09, 32.66. HRMS (ESI) *m/z* calcd for $C_{15}H_{16}NO_5$ $[M+H]^+$: 290.1028, found 290.1014.

Methyl 2-(1,3-dioxoisoindolin-2-yl)-3-(oxetan-3-yl)propanoate (15)

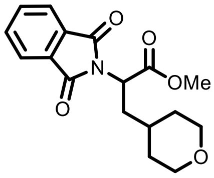

Prepared according to general procedure A, using 4-bromotetrahydropyran (141 µL, 1.25 mmol, 2.5 equiv). The crude mixture was purified by flash column chromatography (100% petroleum ether to petroleum ether 80:20 EtOAc) to afford product **15** (105.7 mg, 67% yield) as a pale yellow oil. ^{1}H NMR (600 MHz, $CDCl_3$) δ 7.88 (dd, *J* = 5.4, 3.1 Hz, 2H), 7.76 (dd, *J* = 5.4, 3.4 Hz, 2H), 4.97 (dd, *J* = 11.4, 4.4 Hz, 1H), 3.97 – 3.87 (m, 2H), 3.73 (s, 3H), 3.28 (tdd, *J* = 11.7, 7.5, 2.4 Hz, 2H), 2.33 (ddd, *J* = 15.1, 11.4, 4.0 Hz, 1H), 2.10 (ddd, *J* = 14.2, 9.5, 4.4 Hz, 1H), 1.78 (d, *J* = 2.1 Hz, 1H), 1.52 (d, *J* = 2.6 Hz, 1H), 1.44 – 1.24 (m, 3H). ^{13}C NMR (151 MHz, $CDCl_3$) δ 170.15, 167.87, 134.44, 131.93, 123.79, 67.92, 67.84, 52.97, 49.52, 35.72, 33.33, 32.00, 31.88. HRMS (ESI) *m/z* calcd for $C_{17}H_{20}NO_5$ $[M+H]^+$: 318,1336, found 318,1368.

***tert*-Butyl 3-(2-(1,3-dioxoisoindolin-2-yl)-3-methoxy-3-oxopropyl)azetidine-1-carboxylate (16)**

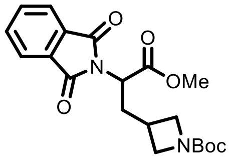

Prepared according to general procedure A, using *tert*-butyl 3-bromoazetidine-1-carboxylate (205 µL, 1.25 mmol, 2.5 equiv). The crude mixture was purified by flash column chromatography (100% petroleum ether to petroleum ether 75:25 EtOAc) to afford product **16** (108.5 mg, 56% yield) as a pale yellow oil. ^{1}H NMR (600 MHz, $CDCl_3$) δ 7.86 (dd, *J* = 7.0, 3.5 Hz, 2H), 7.75 (dd, *J* = 6.7, 3.8 Hz, 2H), 4.76 (t, 1H), 4.00 – 3.94 (m, 1H), 3.92 – 3.87 (m, 1H), 3.73 (s, 3H), 3.64 (dd, *J* = 9.3, 4.3 Hz, 1H), 3.51 (dd, 1H), 2.53 – 2.45 (m, 3H), 1.39 (s, 9H). ^{13}C NMR (151 MHz, $CDCl_3$) δ 169.30, 167.57, 156.30, 134.54, 131.73, 123.83, 79.52, 54.26, 53.95, 53.03, 50.25, 33.34, 28.48, 26.43. HRMS (ESI) *m/z* calcd for $C_{20}H_{25}N_2O_6$ $[M+H]^+$: 389.1713, found 389.1704.

***tert*-Butyl 4-(2-(1,3-dioxoisoindolin-2-yl)-3-methoxy-3-oxopropyl)piperidine-1-carboxylate (17)**

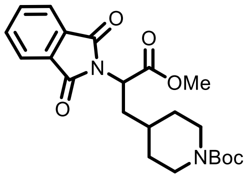

Prepared according to general procedure A, using *tert*-butyl 4-bromopiperidine-1-carboxylate (330 mg, 1.25 mmol, 2.5 equiv). The crude mixture was purified by flash column chromatography (100% petroleum ether to petroleum ether 80:20 EtOAc) to afford product **17** (138.8 mg, 67% yield) as a pale yellow oil. ^{1}H NMR (600 MHz, $CDCl_3$) δ 7.87 (dd, *J* = 5.4, 3.1 Hz, 2H), 7.75 (dd, *J* = 5.4, 3.1 Hz, 2H), 4.95 (dd, *J* = 11.3, 4.4 Hz, 1H), 4.03 (dd, *J* = 27.3, 11.8 Hz, 2H), 3.71 (s, 3H), 2.58 (q, *J* = 10.8 Hz, 2H), 2.31 (ddd, *J* = 14.4, 11.3, 4.3 Hz, 1H), 2.07 (ddd, *J* = 14.4, 9.9, 4.4 Hz, 1H), 1.86 – 1.77 (m, 1H), 1.60 – 1.54 (m, 1H), 1.42 (s, 9H), 1.34 – 1.28 (m, 1H), 1.21 – 1.06 (m, 2H). ^{13}C NMR (151 MHz, $CDCl_3$) δ 170.08, 167.83, 154.88, 134.43, 131.89, 123.76, 79.43, 52.94, 49.72, 35.35, 33.00, 32.49, 30.89, 28.55. HRMS (ESI) *m/z* calcd for $C_{22}H_{29}N_2O_6$ $[M+H]^+$: 417,2026, found 417,2030.

Methyl 3-cyclopentyl-2-(1,3-dioxoisoindolin-2-yl)propanoate (18)

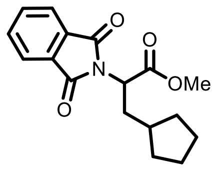

Prepared according to general procedure A, using bromocyclopentane (127 µL, 1.25 mmol, 2.5 equiv). The crude mixture was purified by flash column chromatography (100% cyclohexane to cyclohexane 90:10 EtOAc) to afford product **18** (95.3 mg, 60% yield) as a pale yellow oil. ^{1}H NMR (400 MHz, $CDCl_3$) δ 7.86 (dd, *J* = 5.4, 3.1 Hz, 2H), 7.74 (dd, *J* = 5.5, 3.0 Hz, 2H), 4.89 (dd, *J* = 11.5, 4.2 Hz, 1H), 3.72 (s, 3H), 2.42 (ddd, *J* = 14.0, 11.5, 4.7 Hz, 1H), 2.11 (ddd, *J* = 14.0, 9.3, 4.3 Hz, 1H), 1.86 – 1.76 (m, 1H), 1.74 – 1.64 (m, 2H), 1.62 – 1.52 (m, 2H), 1.51 – 1.39 (m, 2H), 1.22 – 1.05 (m, 2H). ^{13}C NMR (101 MHz, $CDCl_3$) δ 170.24, 167.85, 134.28, 132.00, 123.66, 52.81, 51.92, 37.13, 34.69, 32.87, 32.06, 25.27, 25.11. HRMS (ESI) m/z calcd for $C_{17}H_{20}NO_4$ $[M+H]^+$: 302.1387; found 302.1391.

Methyl 2-(1,3-dioxoisoindolin-2-yl)-4,4-dimethylhexanoate (19)

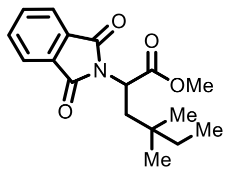

Prepared according to general procedure A, using 2-bromo-2-methylbutane (160 µL, 1.25 mmol, 2.5 equiv). The crude mixture was purified by flash column chromatography (100% pentane to pentane 90:10 EtOAc) to afford product **19** (102.4 mg, 68% yield) as a colorless oil. ^{1}H NMR (600 MHz, $CDCl_3$) δ 7.85 (dd, *J* = 5.5, 3.1 Hz, 2H), 7.72 (dd, *J* = 5.4, 3.0 Hz, 2H), 4.93 (dd, *J* = 8.9, 3.6 Hz, 1H), 3.69 (s, 3H), 2.29 – 2.20 (m, 2H), 1.32 – 1.20 (m, 2H), 0.85 (s, 3H), 0.83 (s, 3H), 0.78 (t, *J* = 7.5 Hz, 3H). ^{13}C NMR (151 MHz, $CDCl_3$) δ 170.73, 167.86, 134.28, 132.00, 123.63, 52.98, 49.32, 39.02, 34.31, 32.99, 26.47, 26.27, 8.35. HRMS (ESI) *m/z* calcd for $C_{17}H_{22}NO_4$ $[M+H]^+$: 304.1549, found 304.1539.

Methyl 2-(1,3-dioxoisoindolin-2-yl)-4,4-dimethyl-6-phenylhexanoate (20)

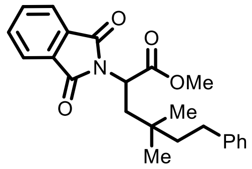

Prepared according to general procedure A, using (3-bromo-3-methylbutyl)benzene (285 mg, 1.25 mmol, 2.5 equiv). The crude mixture was purified by flash column chromatography (100% cyclohexane to cyclohexane 90:10 EtOAc) to afford product **20** (105.2 mg, 53% yield) as a colorless oil. ^{1}H NMR (400 MHz, $CDCl_3$) δ 7.87 (dd, *J* = 5.5, 3.1 Hz, 2H), 7.74 (dd, *J* = 5.4, 3.2 Hz, 2H), 7.23 – 7.17 (m, 2H), 7.15 – 7.05 (m, 3H), 5.00 (dd, *J* = 8.9, 3.5 Hz, 1H), 3.72 (s, 3H), 2.66 – 2.45 (m, 2H), 2.43 – 2.31 (m, 2H), 1.62 – 1.49 (m, 2H), 0.99 (s, 3H), 0.97 (s, 3H). ^{13}C NMR (101 MHz, $CDCl_3$) δ 170.65, 167.90, 142.86, 134.34, 132.01, 128.40, 128.33, 125.69, 123.70, 53.09, 49.31, 44.22, 39.15, 33.19, 30.62, 27.15, 26.89. HRMS (ESI) m/z calcd for $C_{23}H_{26}NO_4$ $[M+H]^+$: 380.1856; found 380.1864.

Methyl 2-(1,3-dioxoisoindolin-2-yl)-3-(1-methylcyclohexyl)propanoate (21)

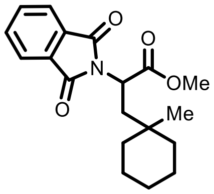

Prepared according to general procedure A, using 2-bromo-2-methylbutane (170 µL, 1.25 mmol, 2.5 equiv). The crude mixture was purified by flash column chromatography (100% petroleum ether to petroleum ether 90:10 EtOAc) to afford product **21** (100.4 mg, 61% yield) as a pale yellow oil. ^{1}H NMR (600 MHz, $CDCl_3$) δ 7.84 (dd, *J* = 5.5, 3.1 Hz, 2H), 7.72 (dd, *J* = 5.5, 3.0 Hz, 2H), 4.95 (dd, *J* = 9.0, 3.3 Hz, 1H), 3.68 (s, 3H), 2.32 – 2.22 (m, 2H), 1.48 – 1.43 (m, 1H), 1.42 – 1.36 (m, 2H), 1.35 – 1.29 (m, 4H), 1.26 – 1.22 (m, 1H), 1.20 – 1.17 (m, 2H), 0.87 (s, 3H). ^{13}C NMR (151 MHz, $CDCl_3$) δ 170.79, 167.82, 134.24, 131.97, 123.59, 52.95, 48.84, 37.70, 37.67, 32.86, 26.28, 21.93, 21.88. HRMS (ESI) *m/z* calcd for $C_{19}H_{24}NO_4$ $[M+H]^+$: 330.1705, found 330.1697.

Methyl 3-(adamantan-1-yl)-2-(1,3-dioxoisoindolin-2-yl)propanoate (22)

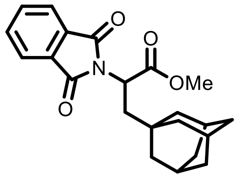

Prepared according to general procedure A, using 1-bromoadamantane (269 mg, 1.25 mmol, 2.5 equiv). The crude mixture was purified by flash column chromatography (100% petroleum ether to petroleum ether 90:10 EtOAc) to afford product **22** (107.2 mg, 58% yield) as a pale yellow oil. ^{1}H NMR (600 MHz, $CDCl_3$) δ 7.86 (dd, *J* = 5.6, 3.1 Hz, 2H), 7.73 (dd, *J* = 5.4, 3.2 Hz, 2H), 5.00 (dd, *J* = 9.4, 2.9 Hz, 1H), 3.69 (s, 3H), 2.22 – 2.06 (m, 2H), 1.94 – 1.86 (m, 3H), 1.67 – 1.45 (m, 12H). ^{13}C NMR (151 MHz, $CDCl_3$) δ 170.90, 167.84, 134.26, 132.06, 123.66, 52.99, 48.07, 42.35, 42.00, 36.92, 32.44, 28.57. HRMS (ESI) *m/z* calcd for $C_{22}H_{26}NO_4$ $[M+H]^+$: 368.1862, found 368.1846.

3-cyclohexyl-2-(1,3-dioxoisoindolin-2-yl)-*N,N*-diethylpropanamide (23)

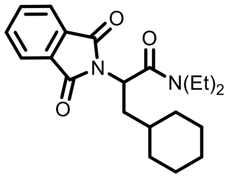

Prepared according to general procedure B, using 2-(1,3-dioxoisoindolin-2-yl)-*N,N*-diethylacrylamide (102.5 mg, 0.38 mmol, 1.0 equiv). The crude mixture was purified by flash column chromatography (100% petroleum ether to petroleum ether 80:20 EtOAc) to afford product **23** (51.9 mg, 39% yield) as a colorless oil. ^{1}H NMR (600 MHz, $CDCl_3$) δ 7.84 (dd, *J* = 5.4, 3.0 Hz, 2H), 7.71 (dd, *J* = 5.5, 3.2 Hz, 2H), 5.17 (dd, *J* = 11.7, 4.1 Hz, 1H), 3.50 – 3.34 (m, 2H), 3.33 – 3.20 (m, 2H), 2.76 – 2.69 (m, 1H), 1.87 (d, 1H), 1.72 – 1.65 (m, 3H), 1.29 – 1.18 (m, 6H), 1.18 – 1.09 (m, 6H), 1.03 – 0.96 (m, 1H), 0.94 – 0.86 (m, 1H). ^{13}C NMR (151 MHz, $CDCl_3$) δ 169.07, 168.63, 134.13, 132.08, 123.51, 50.18, 41.85, 40.71, 36.00, 34.77, 33.85, 32.09, 26.57, 26.28, 26.09, 14.30, 12.98. HRMS (ESI) *m/z* calcd for $C_{21}H_{29}N_2O_3$ $[M+H]^+$: 357.2178, found 357.2216.

Methyl (3-cyclohexyl-2-(1,3-dioxoisoindolin-2-yl)propanoyl)-L-phenylalaninate (24)

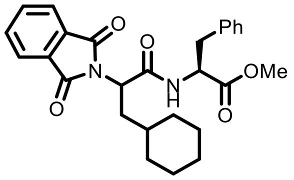

Prepared according to general procedure B, using methyl (2-(1,3-dioxoisoindolin-2-yl)acryloyl)-L-phenylalaninate (121.4 mg, 0.32 mmol, 1.0 equiv). The crude mixture was purified by flash column chromatography (100% petroleum ether to petroleum ether 70:30 EtOAc) to afford product **24** (92.1 mg, 62% yield) as a pale yellow oil (diastereoisomeric ratio = 1.1:0.9). ^{1}H NMR (600 MHz, $CDCl_3$) δ 7.88 – 7.83 (m, 2H), 7.78 – 7.73 (m, 2H), 7.15 – 6.99 (m, 5H), 6.63 (d, *J* = 7.5 Hz, 1H major), 6.51 (d, *J* = 7.5 Hz, 1H minor), 4.93 – 4.80 (m, 2H), 3.73 (s, 3H major), 3.70 (s, 3H minor), 3.21 – 3.11 (m, 1H), 3.10 – 3.03 (m, 1H), 2.29 (ddd, *J* = 14.1, 11.1, 4.6 Hz, 1H major), 2.21 (ddd, *J* = 15.2, 10.7, 4.8 Hz, 1H minor), 1.90 – 1.74 (m, 2H), 1.73 – 1.54 (m, 4H), 1.17 – 1.04 (m, 4H), 0.99 – 0.82 (m, 2H). ^{13}C NMR (151 MHz, $CDCl_3$) δ 171.85, 171.80, 169.14, 169.00, 168.26, 168.14, 135.79, 135.75, 134.42, 134.38, 131.82, 131.79, 129.41, 129.33, 128.60, 128.52, 127.19, 127.14, 123.79, 123.75, 53.45, 52.78, 52.53, 52.51, 52.43, 37.82, 37.67, 36.23, 36.05, 34.70, 34.62, 33.66, 33.61, 32.18, 32.09, 26.45, 26.20, 26.01. HRMS (ESI) *m/z* calcd for $C_{27}H_{31}N_2O_5$ $[M+H]^+$: 463.2233, found 463.2235.

Methyl 2-(bis(*tert*-butoxycarbonyl)amino)-3-cyclohexylpropanoate (25)

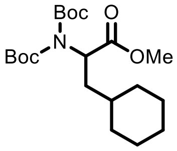

Prepared according to general procedure B, using methyl 2-(bis(*tert*-butoxycarbonyl)amino)acrylate (151 mg, 0.50 mmol, 1.0 equiv). The crude mixture was purified by flash column chromatography (100% cyclohexane to cyclohexane 90:10 EtOAc) to afford product **25** (61.7 mg, 32% yield) as a pale yellow oil. ^{1}H NMR (400 MHz, $CDCl_3$) δ 4.95 (dd, *J* = 9.6, 4.9 Hz, 1H), 3.69 (s, 3H), 2.00 – 1.90 (m, 1H), 1.85 – 1.73 (m, 2H), 1.71 – 1.58 (m, 3H), 1.49 (s, 18H), 1.31 – 1.09 (m, 5H), 1.03 – 0.93 (m, 1H), 0.93 – 0.81 (m, 1H). ^{13}C NMR (101 MHz, $CDCl_3$) δ 171.03, 151.21, 82.10, 55.09, 51.25, 36.80, 33.58, 33.07, 31.78, 27.12, 25.62, 25.50, 25.25. HRMS (ESI) m/z calcd for $C_{20}H_{36}NO_6$ $[M+H]^+$: 386.2537; found 386.2529.

Methyl 2-(((benzyloxy)carbonyl)(*tert*-butoxycarbonyl)amino)-3-cyclohexylpropanoate (26)

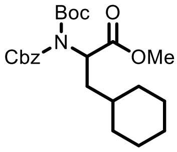

Prepared according to general procedure B, using methyl 2-(((benzyloxy)carbonyl)(*tert*-butoxycarbonyl)amino)acrylate (168 mg, 0.50 mmol, 1.0 equiv). The crude mixture was purified by flash column chromatography (100% cyclohexane to cyclohexane 90:10 EtOAc) to afford product **26** (180.1 mg, 86% yield) as a pale yellow oil. ^{1}H NMR (400 MHz, $CDCl_3$) δ 7.42 – 7.30 (m, 5H), 5.27 – 5.23 (m, 2H), 5.02 (dd, *J* = 9.4, 4.9 Hz, 1H), 3.64 (s, 3H), 1.96 (ddd, *J* = 14.1, 9.0, 4.9 Hz, 1H), 1.82 – 1.73 (m, 2H), 1.68 – 1.60 (m, 3H), 1.46 (s, 9H), 1.27 – 1.09 (m, 5H), 0.98 – 0.82 (m, 2H). ^{13}C NMR (101 MHz, $CDCl_3$) δ 170.64, 152.93, 150.54, 134.41, 127.64, 127.50, 127.42, 82.73, 67.86, 55.43, 51.31, 36.62, 33.52, 32.95, 31.68, 27.00, 25.56, 25.39, 25.15. HRMS (ESI) m/z calcd for $C_{23}H_{34}NO_6$ $[M+H]^+$: 420.2381; found 420.2388.

1-Benzyl 2-methyl 3-cyclohexylpyrrolidine-1,2-dicarboxylate (27)

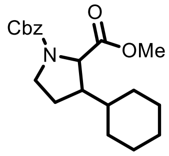

Prepared according to general procedure B, using 1-benzyl 2-methyl 4,5-dihydro-1*H*-pyrrole-1,2-dicarboxylate (131 mg, 0.50 mmol, 1.0 equiv). The crude mixture was purified by flash column chromatography (100% cyclohexane to cyclohexane 90:10 EtOAc) to afford product **27** (60.5 mg, 35% yield) as a pale yellow oil (diastereoisomeric ratio = 1:1). ^{1}H NMR (400 MHz, $CDCl_3$) δ 7.37 – 7.28 (m, 5H), 5.20 – 5.00 (m, 2H), 4.50 (d, *J* = 7.6 Hz, 1H diastereomer A), 4.44 (d, *J* = 7.7 Hz, 1H diastereomer B), 3.79 – 3.74 (m, 1H), 3.73 (s, 3H diastereomer A), 3.58 (s, 3H diastereomer B), 3.35 (tt, *J* = 10.4, 7.0 Hz, 1H), 2.13 – 2.01 (m, 2H), 2.01 – 1.92 (m, 1H), 1.89 – 1.77 (m, 1H), 1.75 – 1.63 (m, 4H), 1.24 – 1.12 (m, 3H), 1.04 – 0.89 (m, 3H). ^{13}C NMR (101 MHz, $CDCl_3$) δ 172.72, 172.47, 155.04, 154.39, 136.89, 136.80, 128.59, 128.54, 128.08, 128.04, 128.02, 127.86, 67.11, 67.02, 61.94, 61.77, 51.89, 51.72, 50.03, 49.16, 46.49, 46.07, 38.76, 38.70, 32.84, 32.80, 31.60, 31.58, 27.76, 26.94, 26.45, 26.43, 26.20, 26.18, 26.09, 26.02. HRMS (ESI) m/z calcd for $C_{20}H_{28}NO_4$ $[M+H]^+$: 346.2013; found 346.2019.

6. NMR spectra

Methyl 2-(bis(*tert*-butoxycarbonyl)amino)acrylate, ^{1}H NMR (600 MHz, $CDCl_3$)

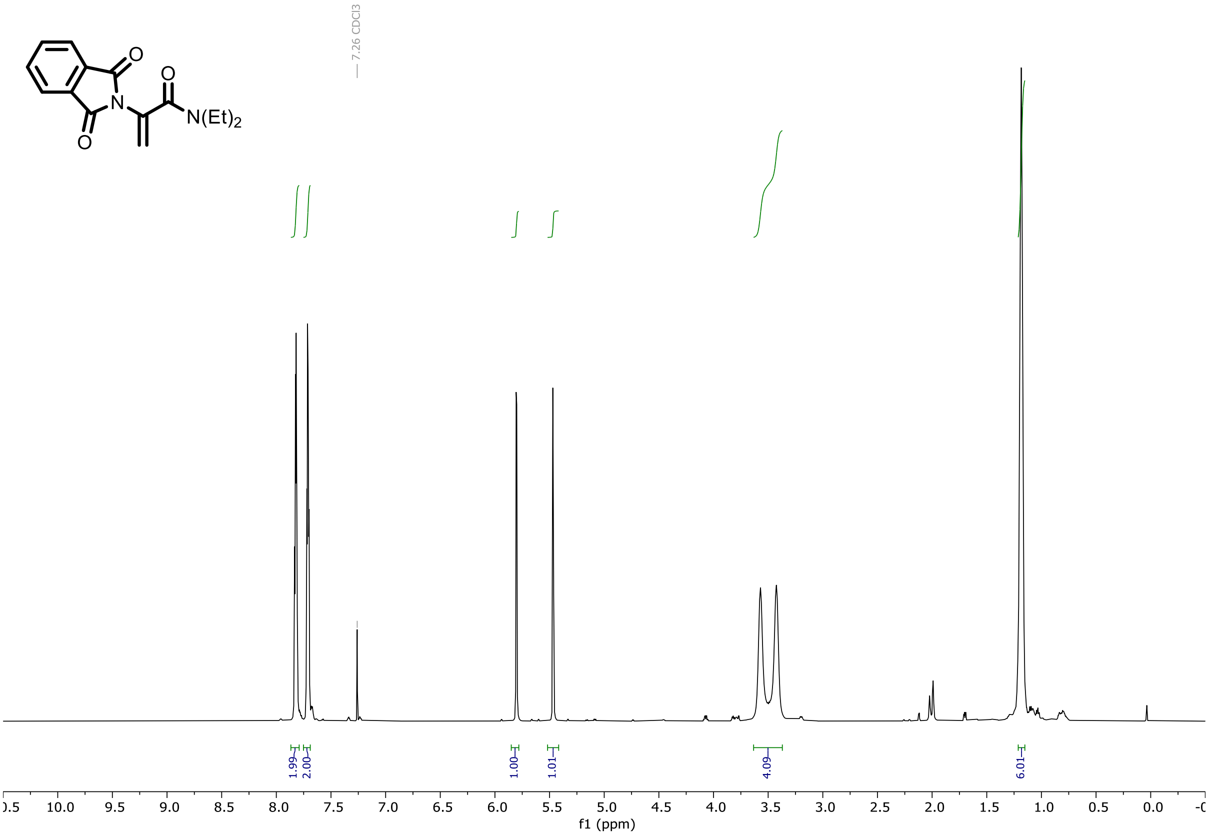

Methyl 2-(bis(*tert*-butoxycarbonyl)amino)acrylate, ^{13}C NMR (151 MHz, $CDCl_3$)

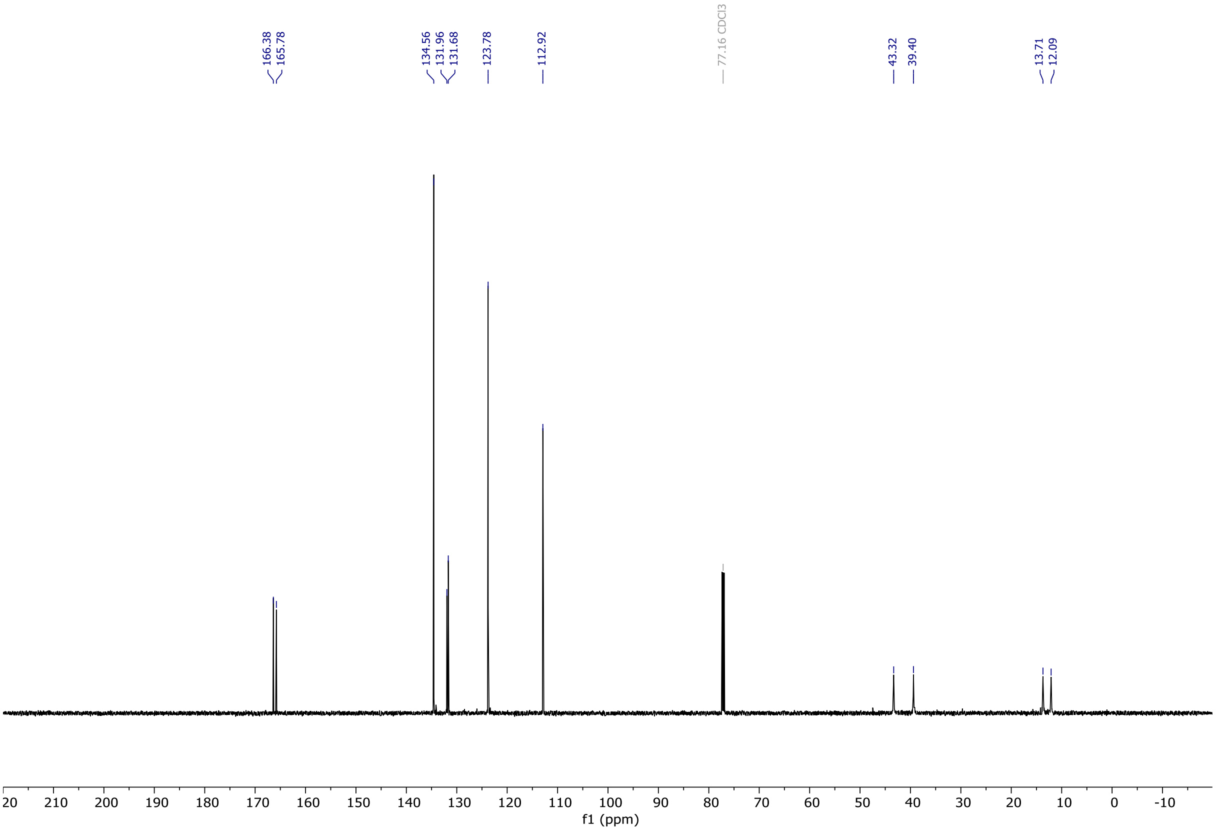

3, ^{1}H NMR (400 MHz, $CDCl_3$)

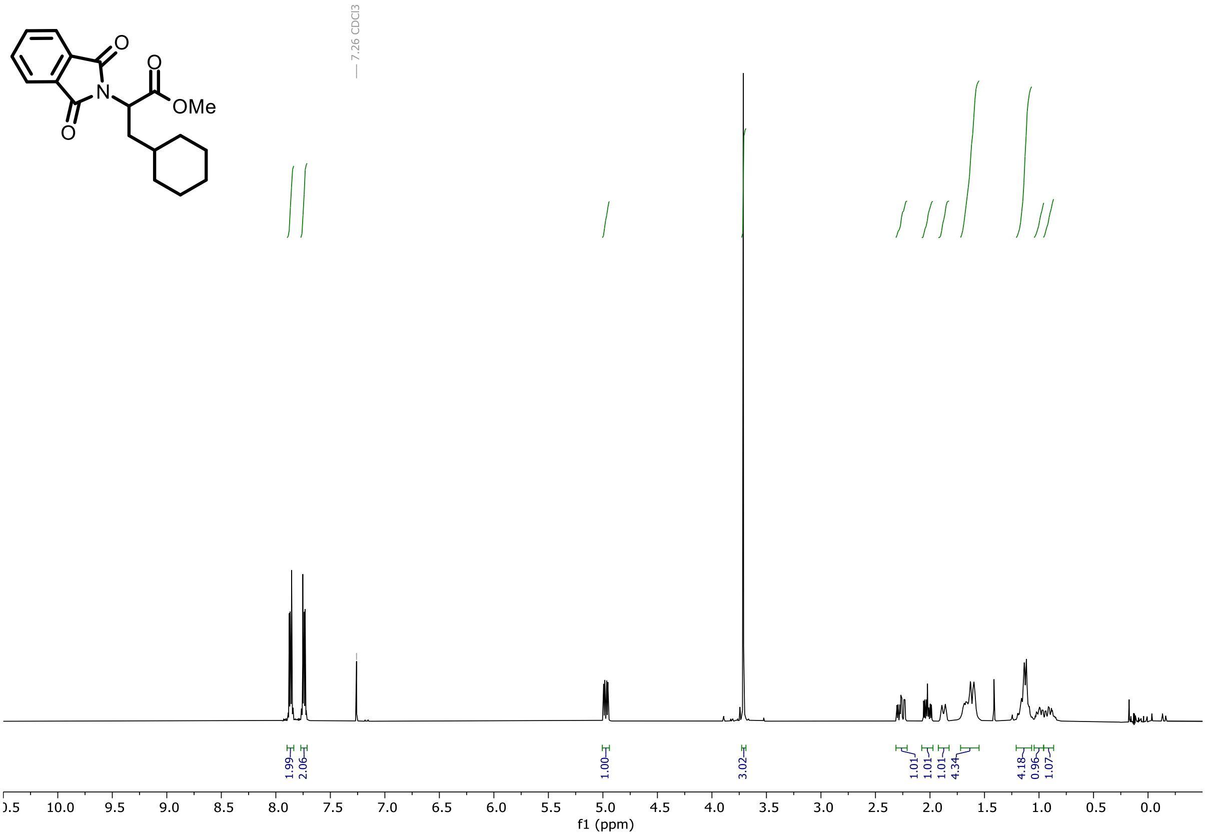

3, ^{13}C NMR (101 MHz, $CDCl_3$)

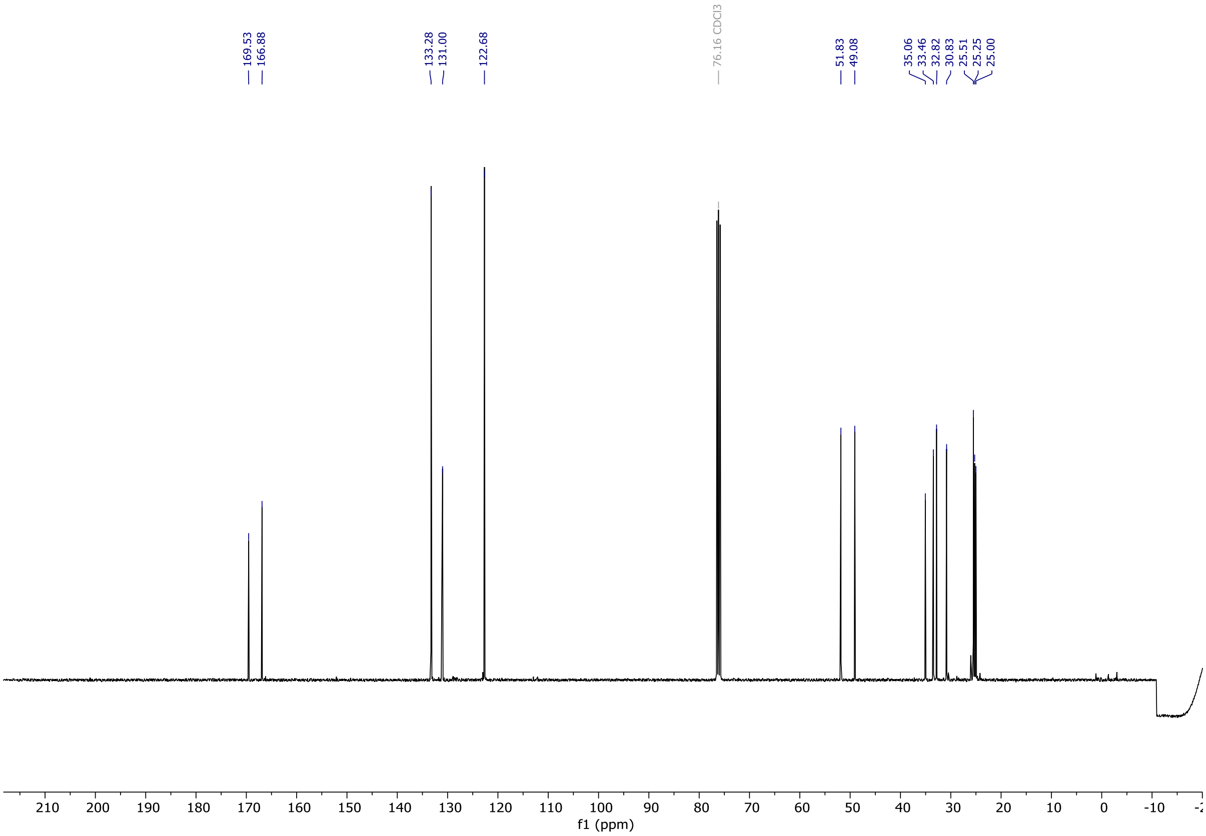

4, ^{1}H NMR (400 MHz, $CDCl_3$)

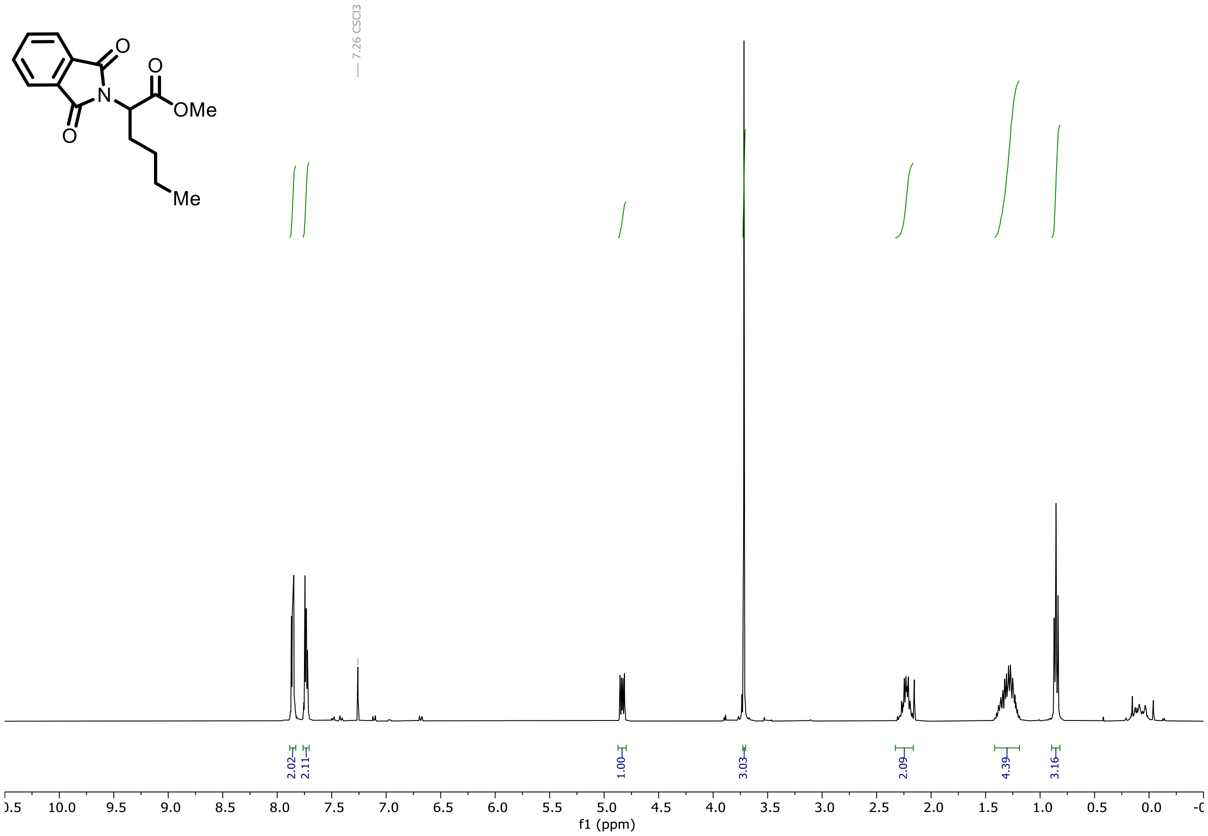

4, ^{13}C NMR (101 MHz, $CDCl_3$)

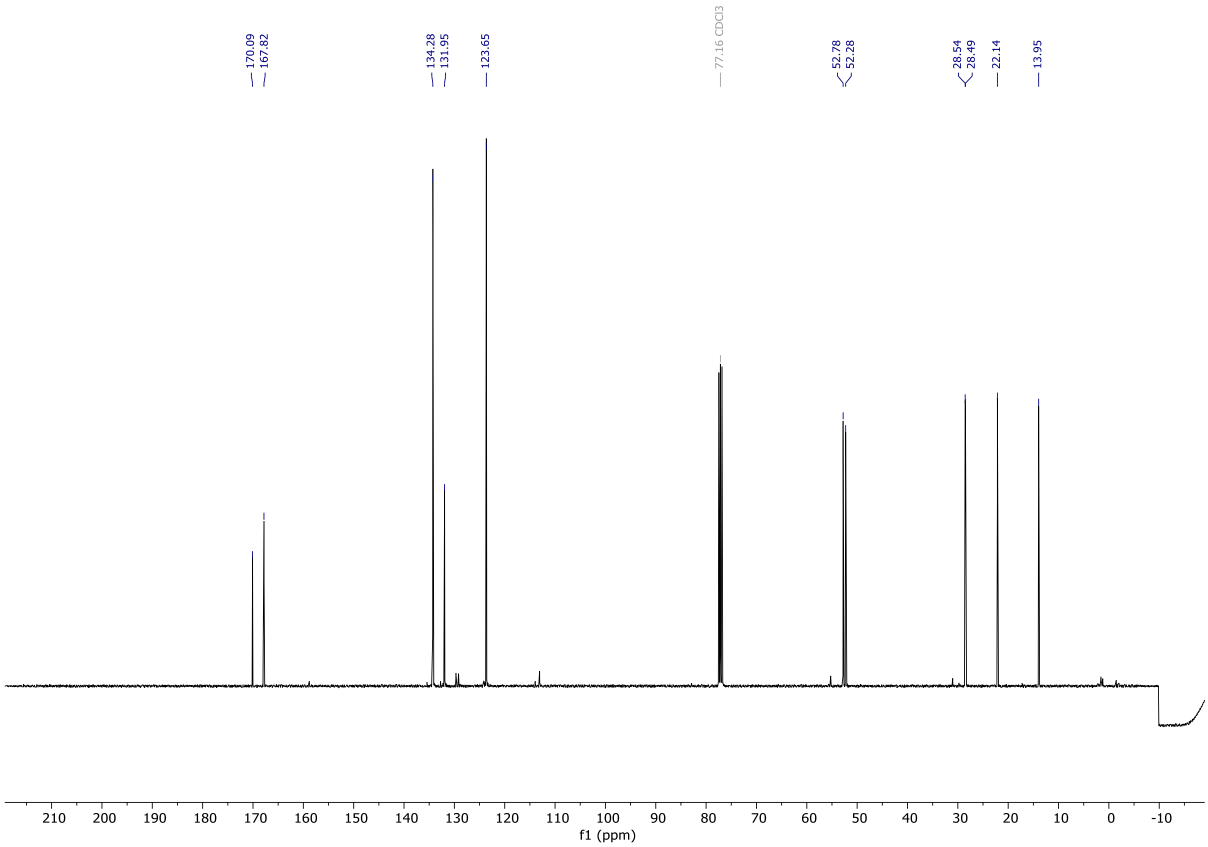

5, ^{1}H NMR (400 MHz, $CDCl_3$)

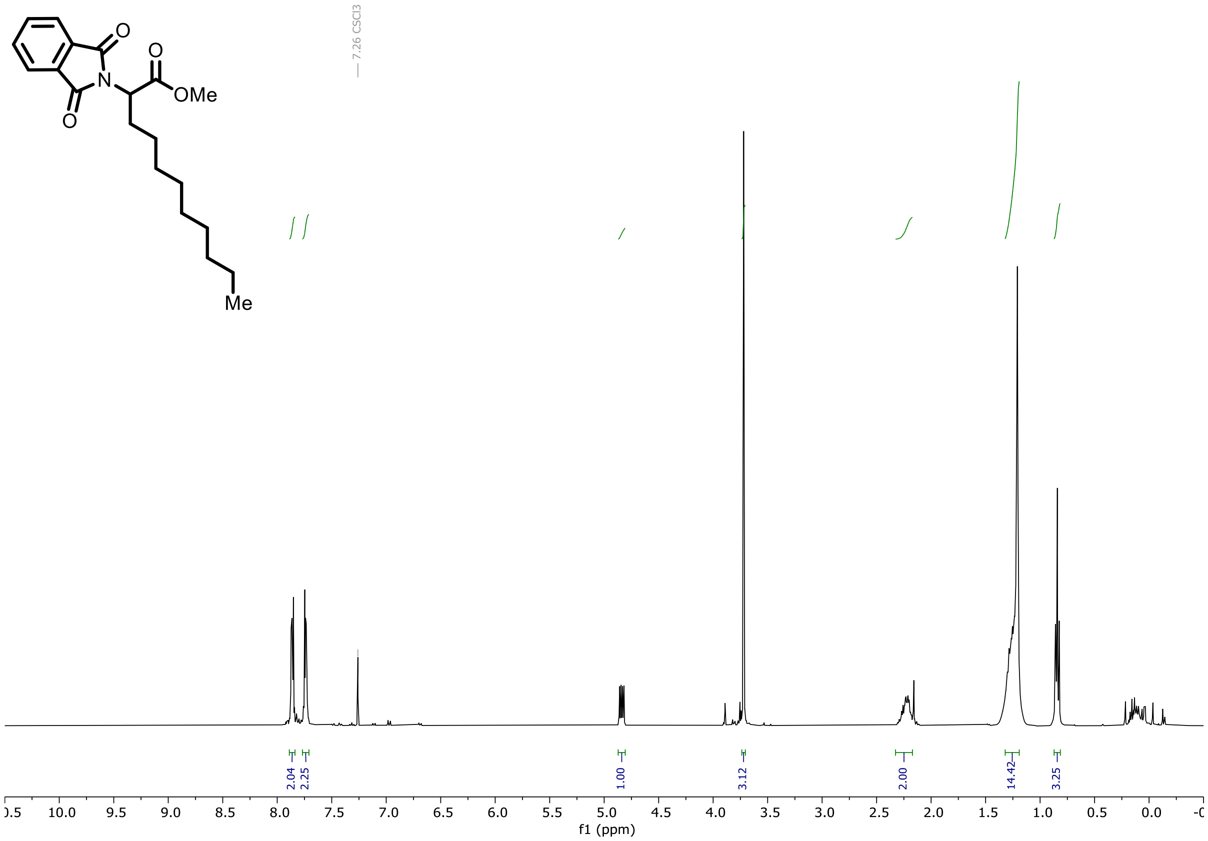

5, ^{13}C NMR (101 MHz, $CDCl_3$)

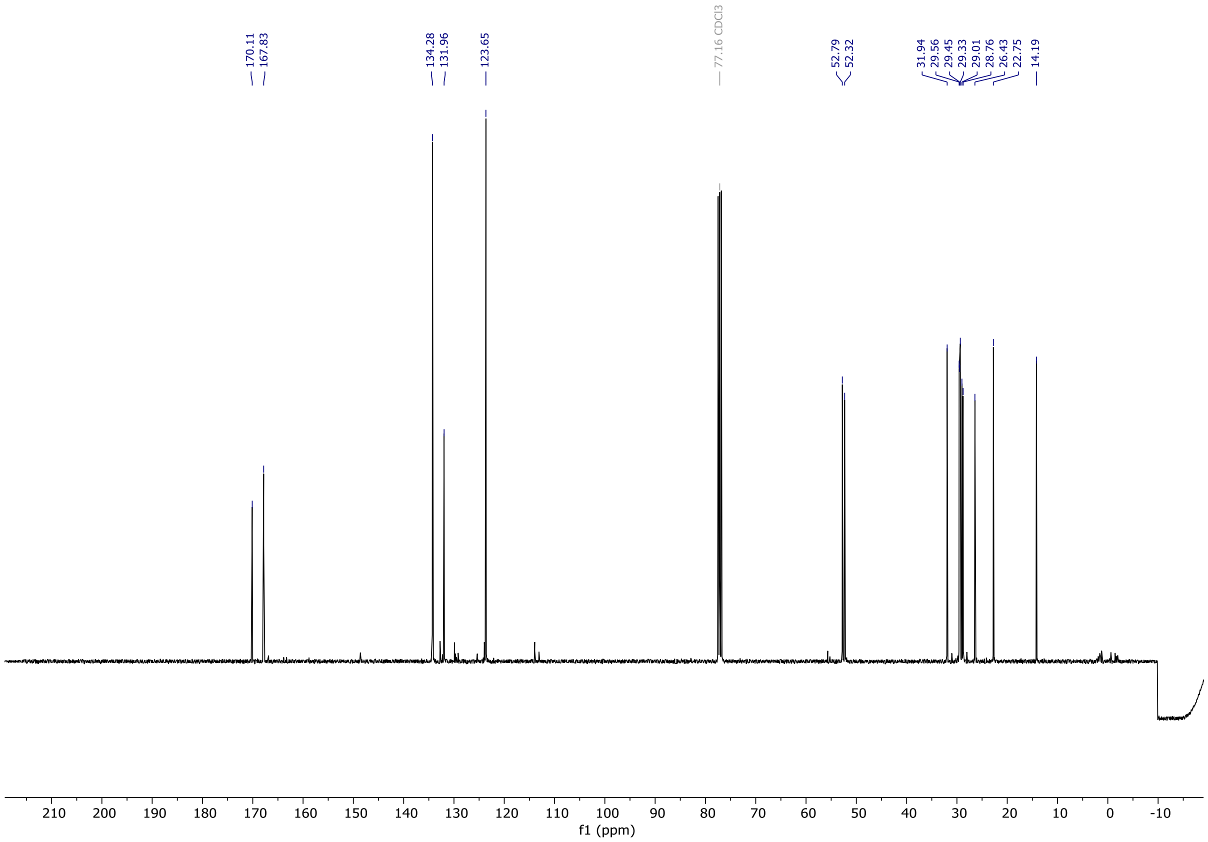

6, ^{1}H NMR (400 MHz, $CDCl_3$)

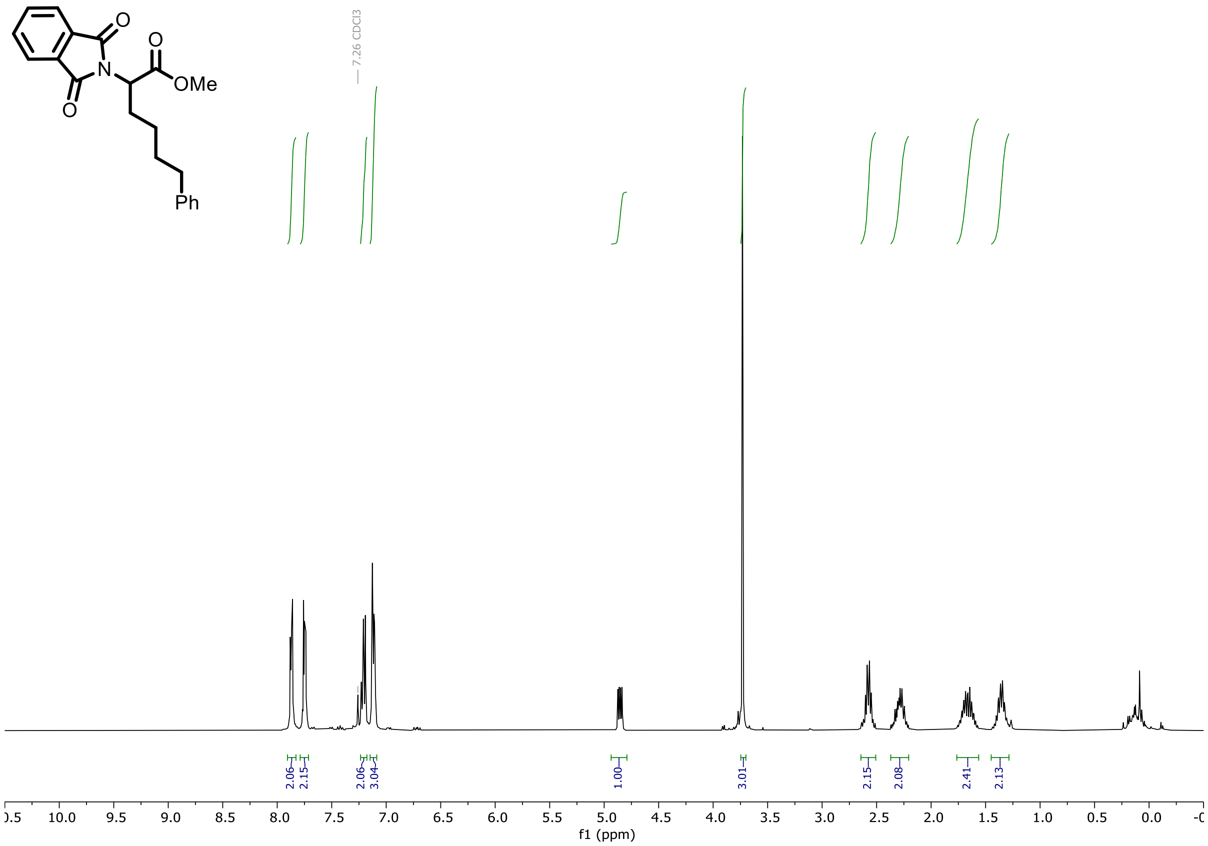

6, ^{13}C NMR (101 MHz, $CDCl_3$)

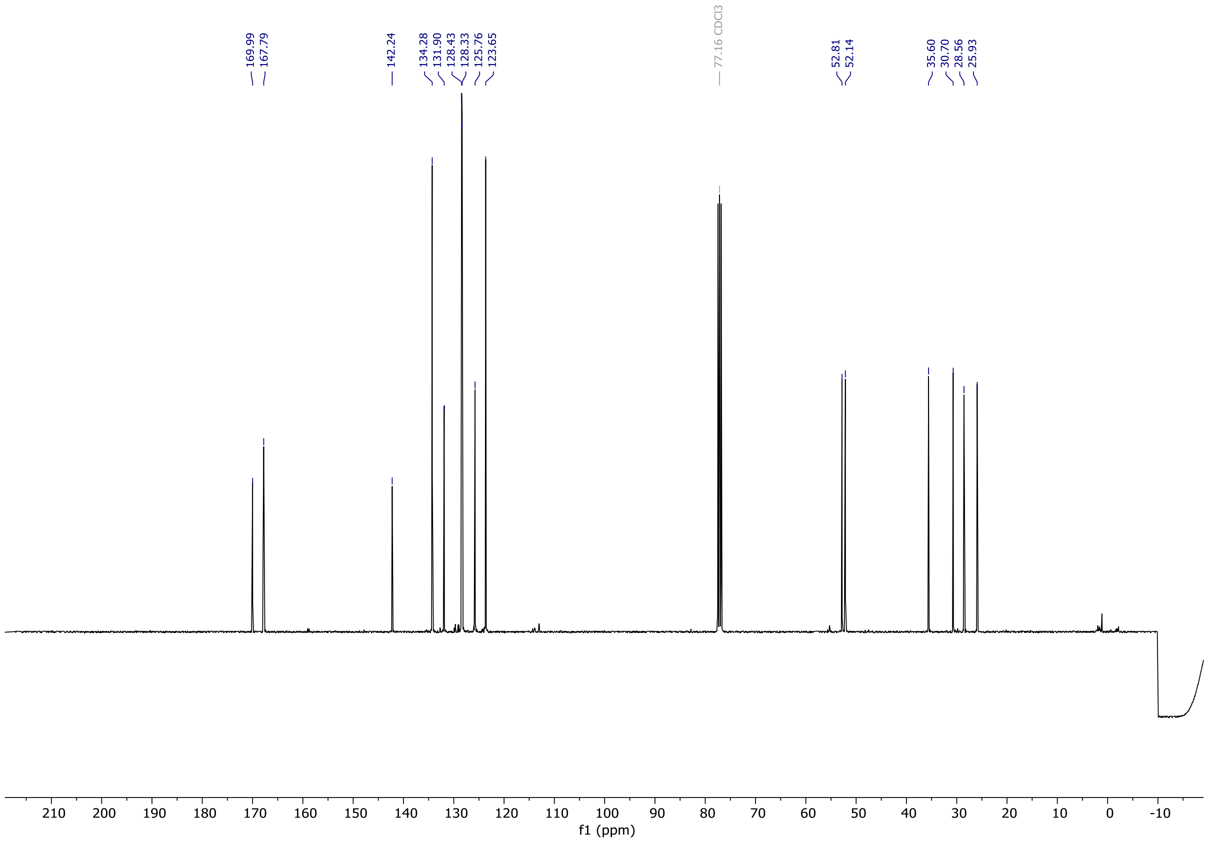

7, ^{1}H NMR (600 MHz, CDCl$_3$)

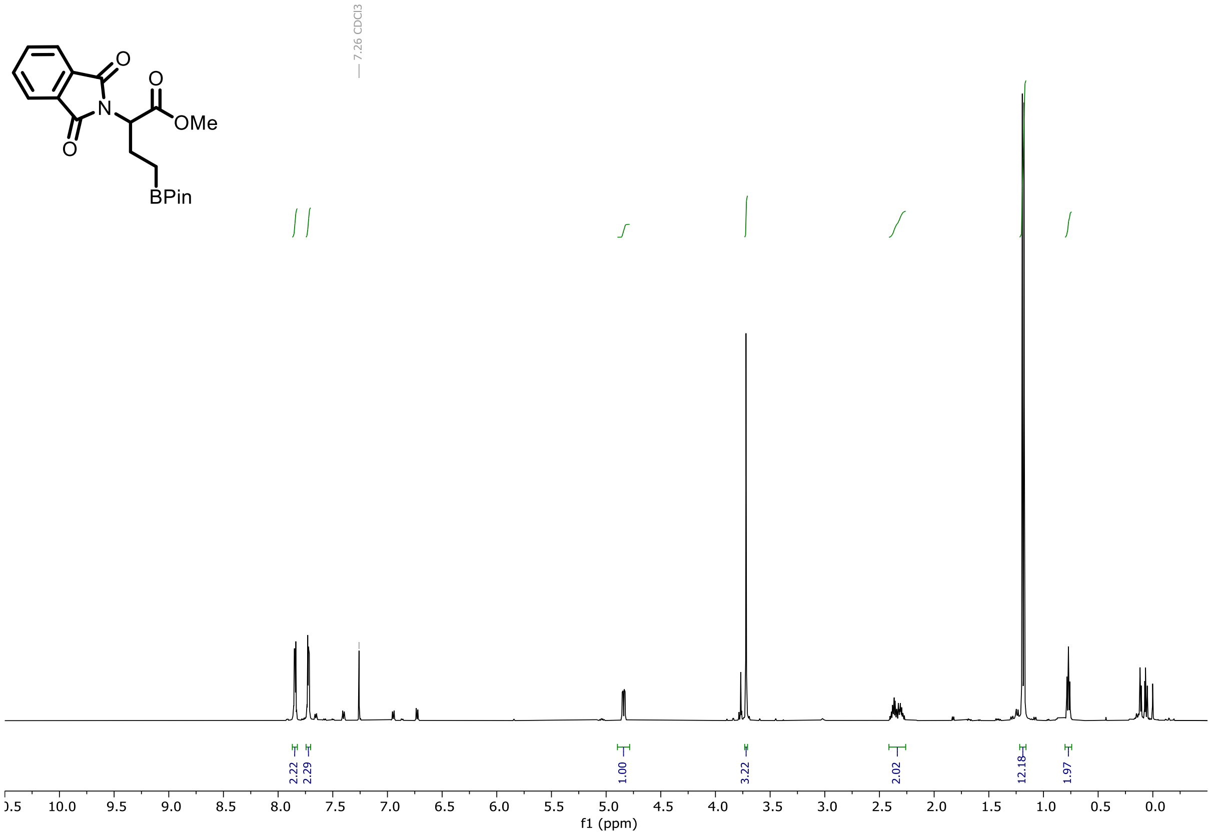

7, ^{13}C NMR (151 MHz, CDCl$_3$)

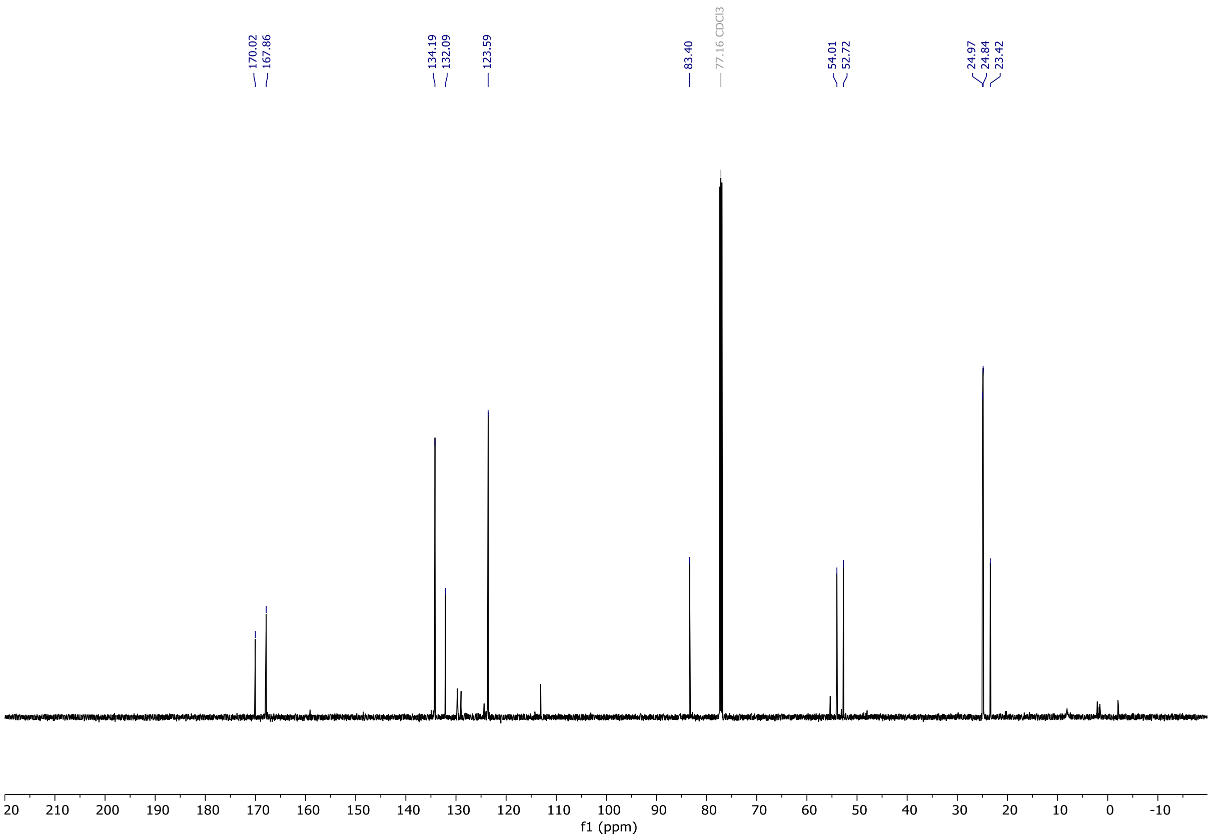

8, ^{1}H NMR (600 MHz, $CDCl_3$)

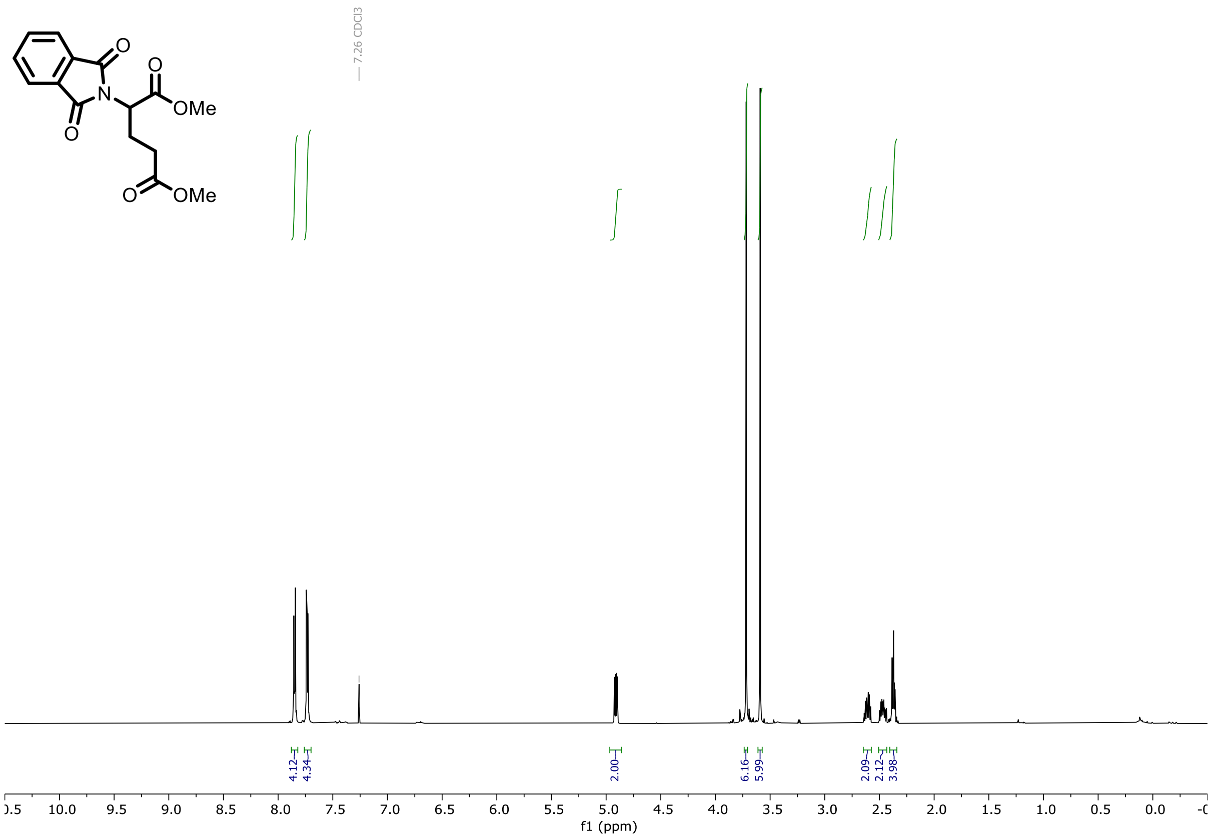

8, ^{13}C NMR (151 MHz, $CDCl_3$)

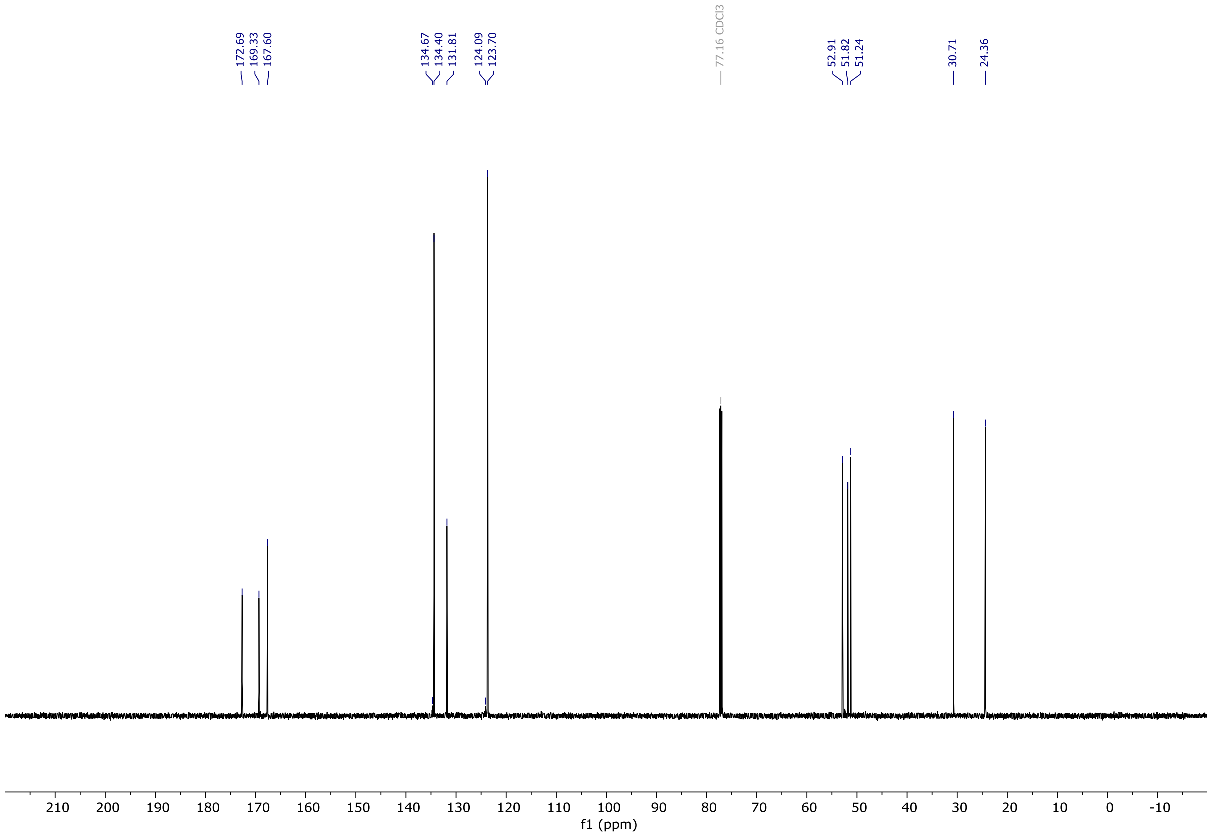

9, ^{1}H NMR (600 MHz, $CDCl_3$)

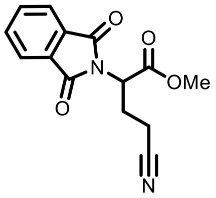

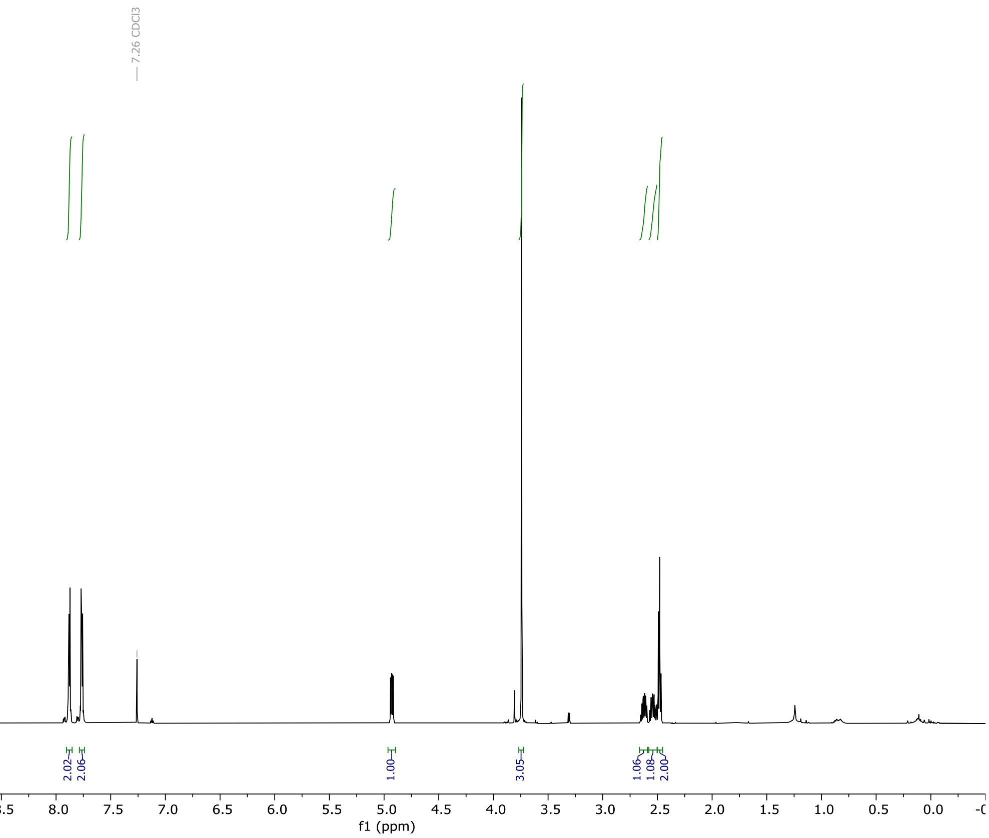

9, ^{13}C NMR (151 MHz, $CDCl_3$)

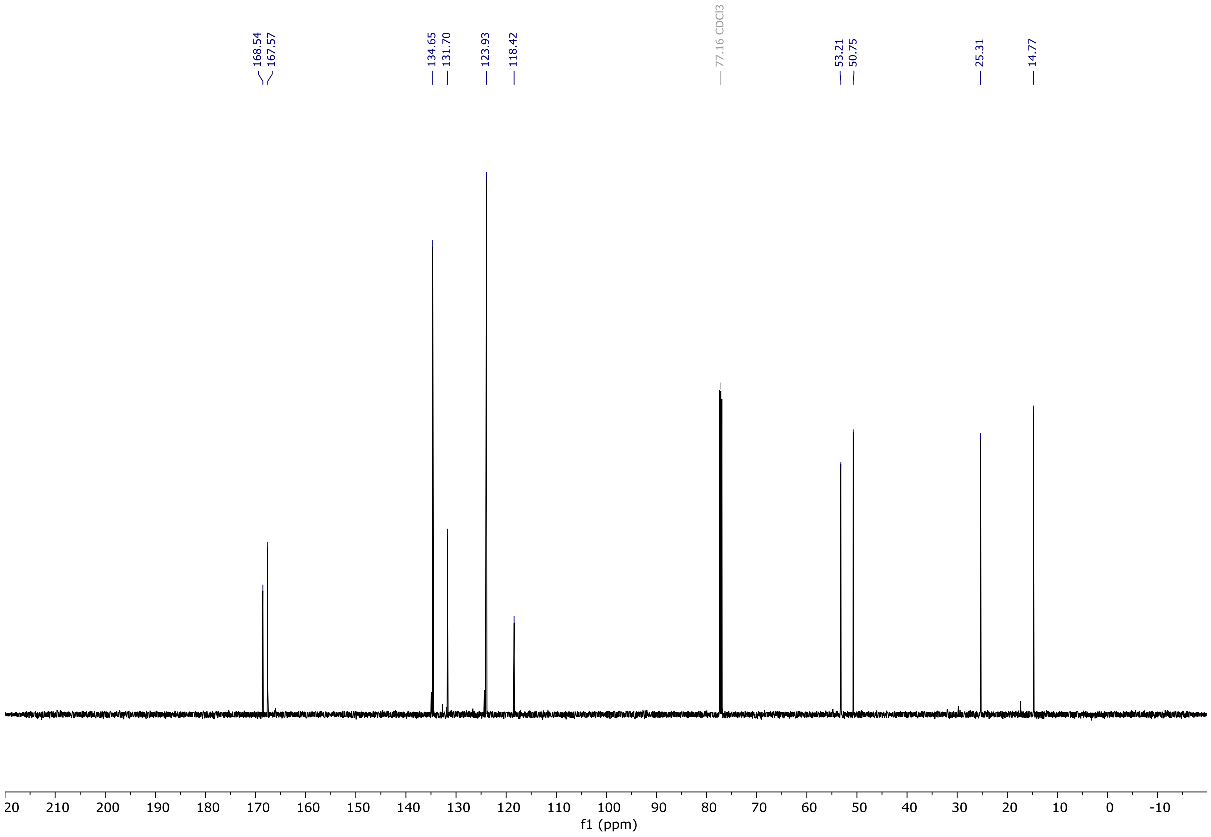

10, ^{1}H NMR (600 MHz, $CDCl_3$)

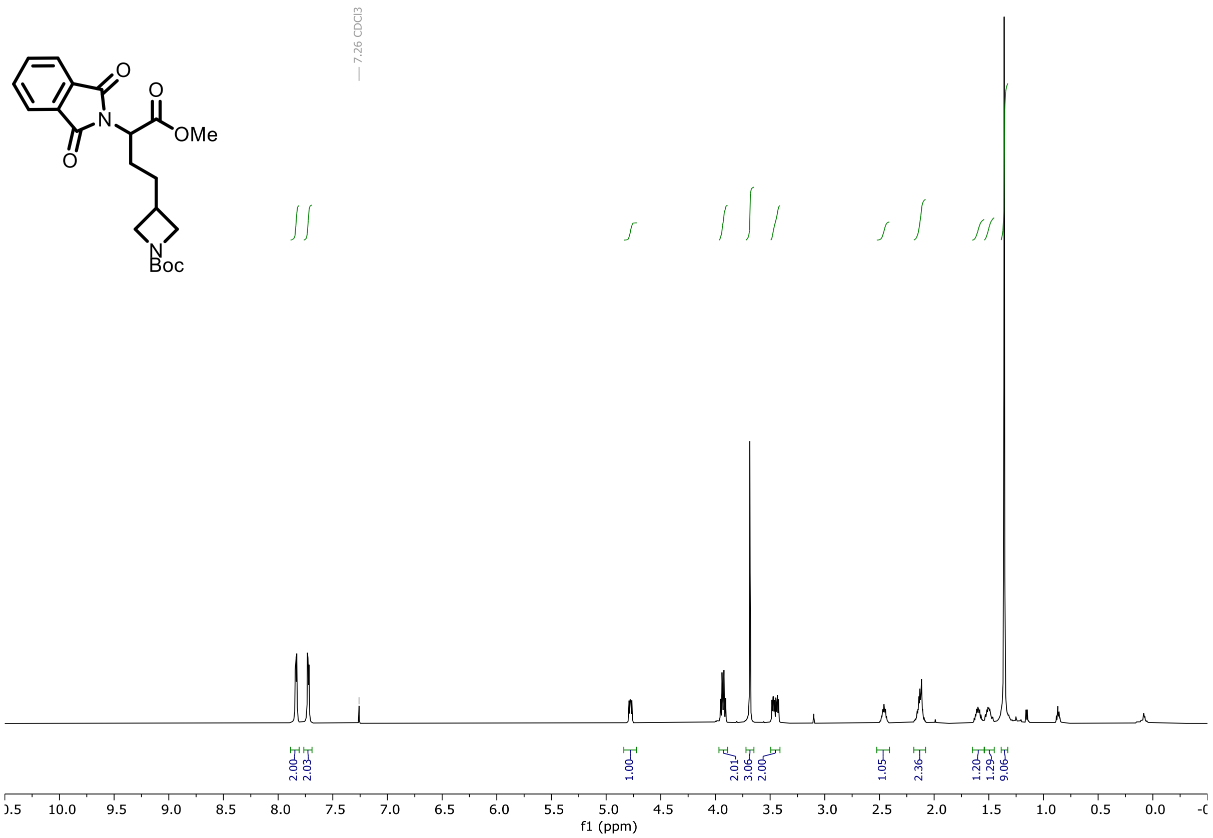

10, ^{13}C NMR (151 MHz, $CDCl_3$)

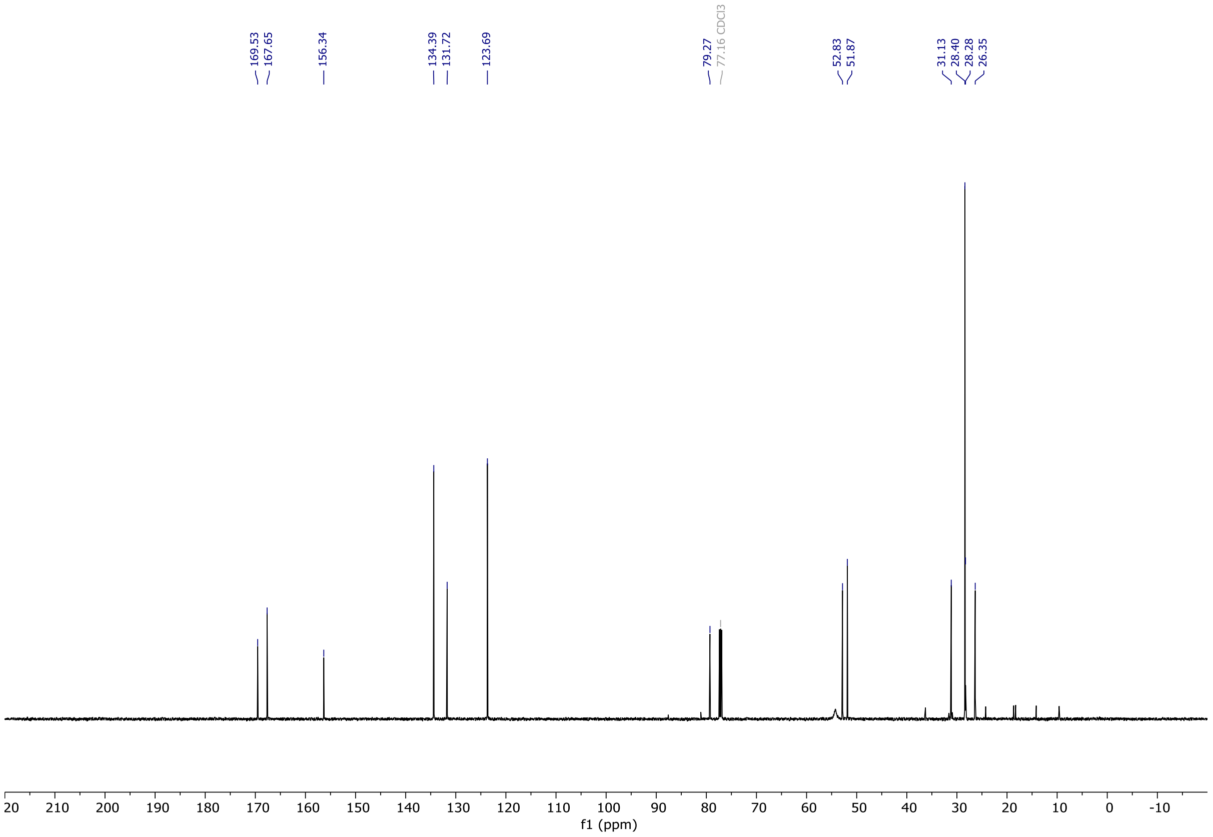

11, ^{1}H NMR (400 MHz, $CDCl_3$)

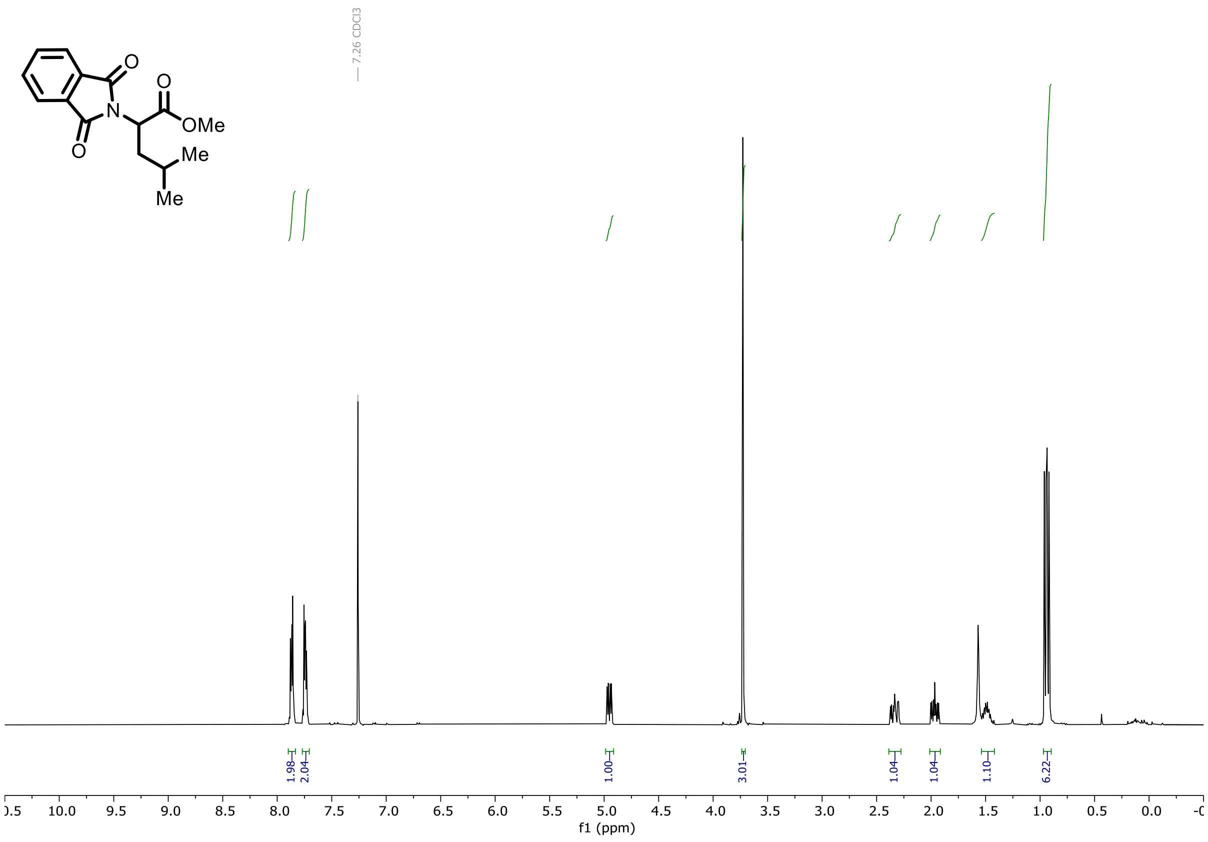

11, ^{13}C NMR (101 MHz, $CDCl_3$)

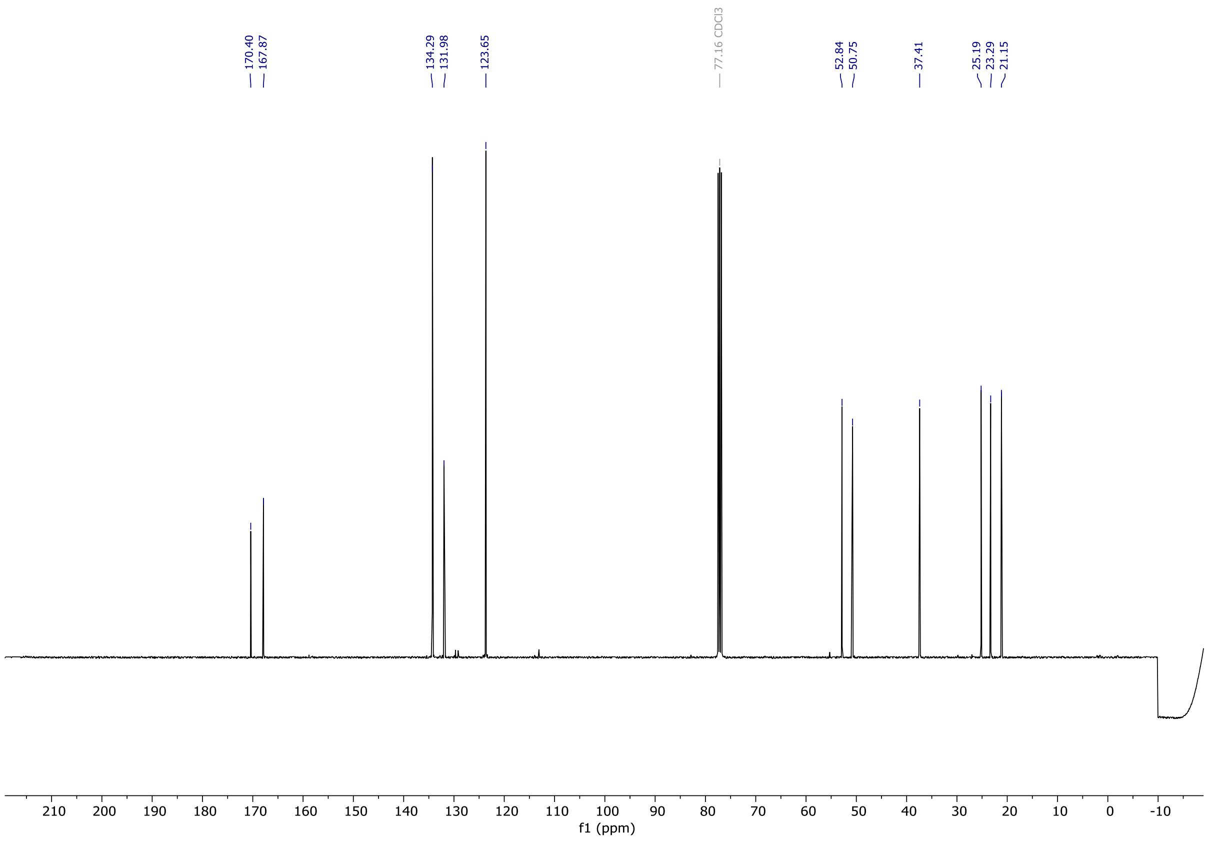

12, ^{1}H NMR (400 MHz, $CDCl_3$)

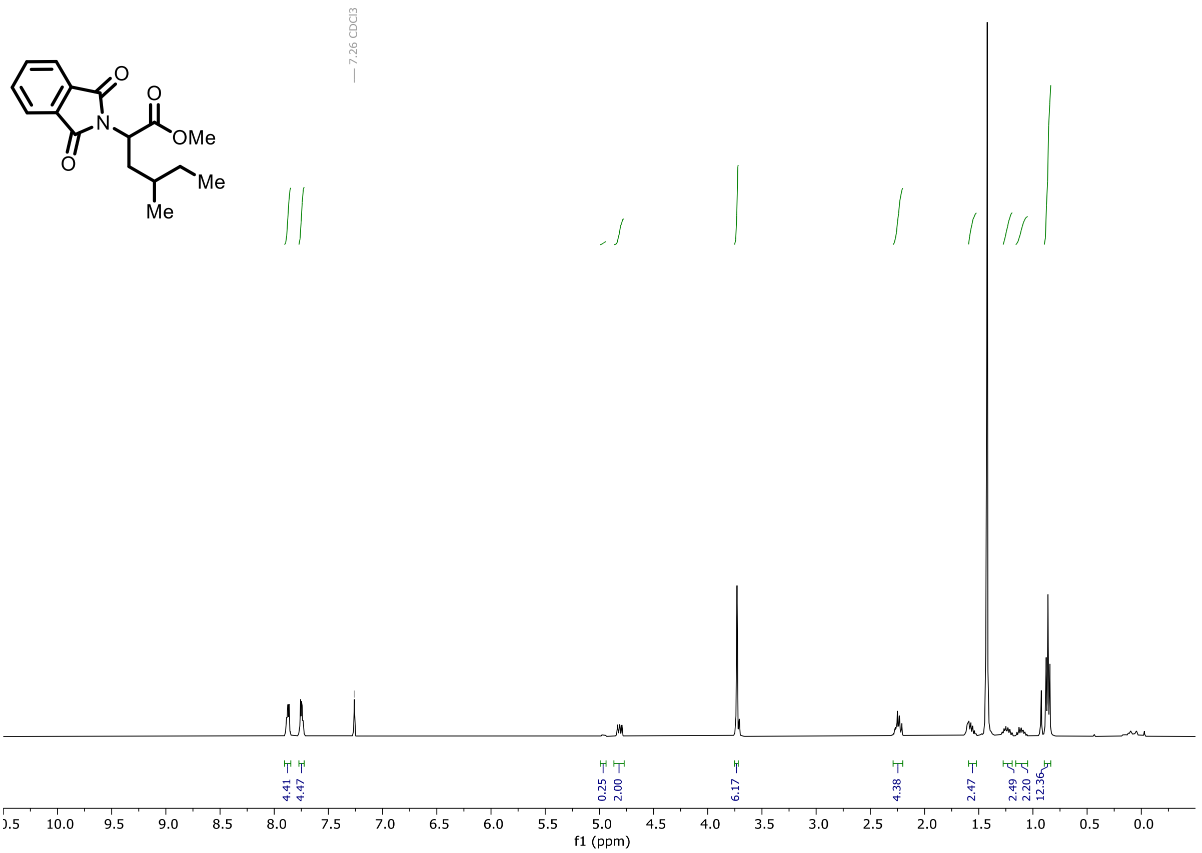

12, ^{13}C NMR (101 MHz, $CDCl_3$)

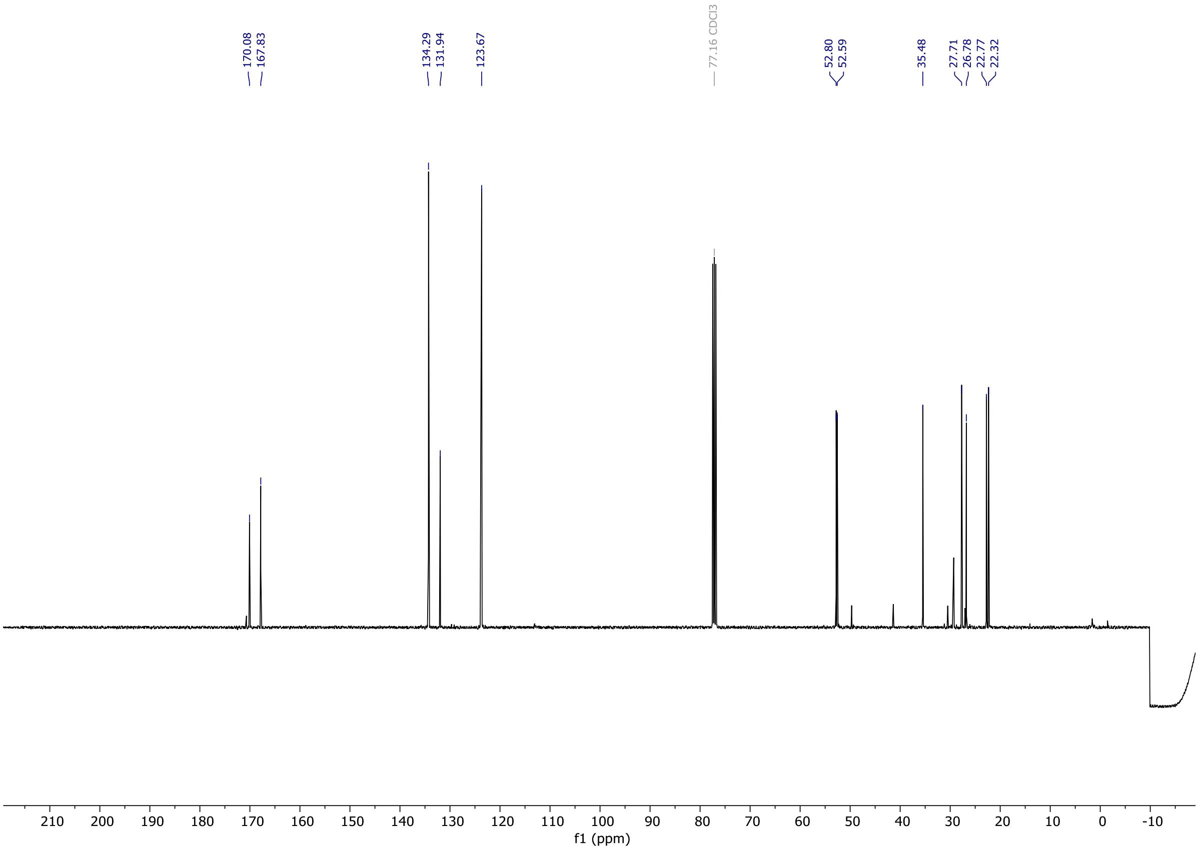

13, ^{1}H NMR (400 MHz, $CDCl_3$)

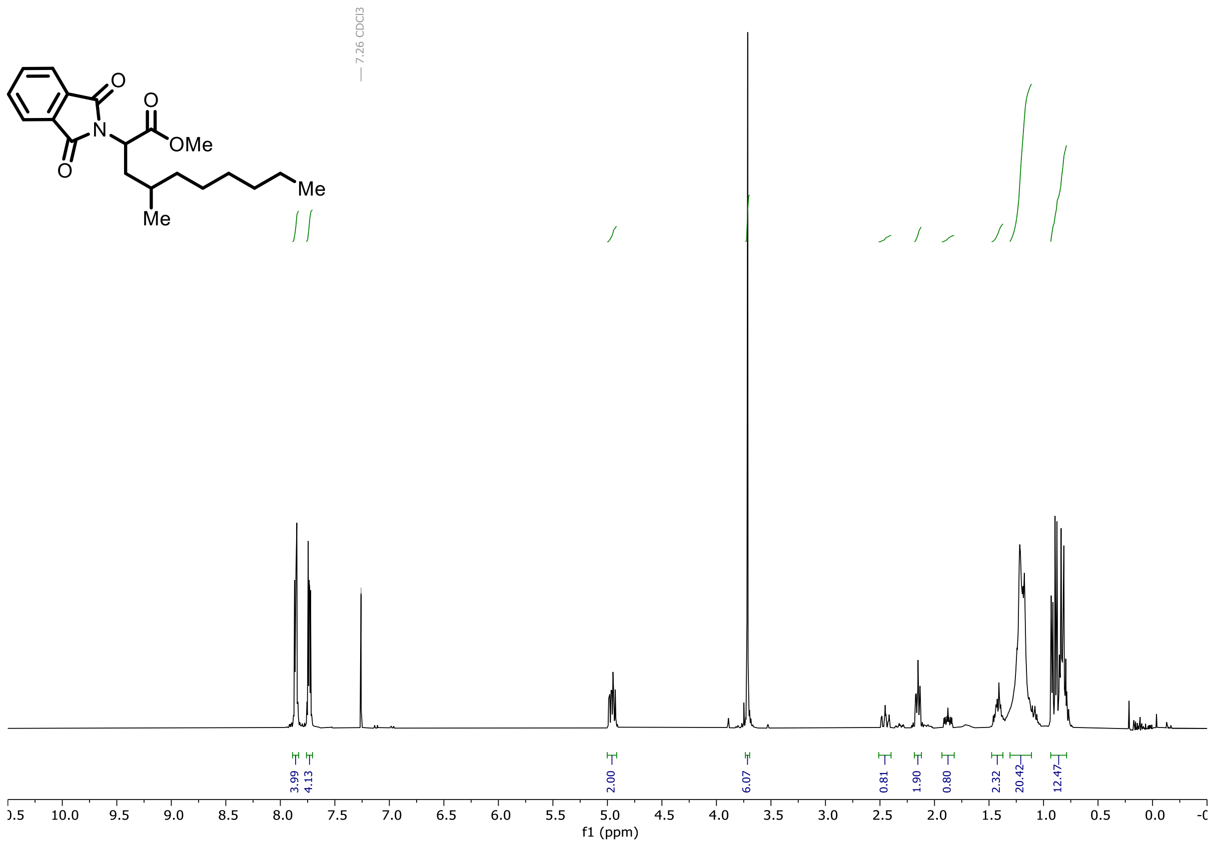

13, ^{13}C NMR (101 MHz, $CDCl_3$)

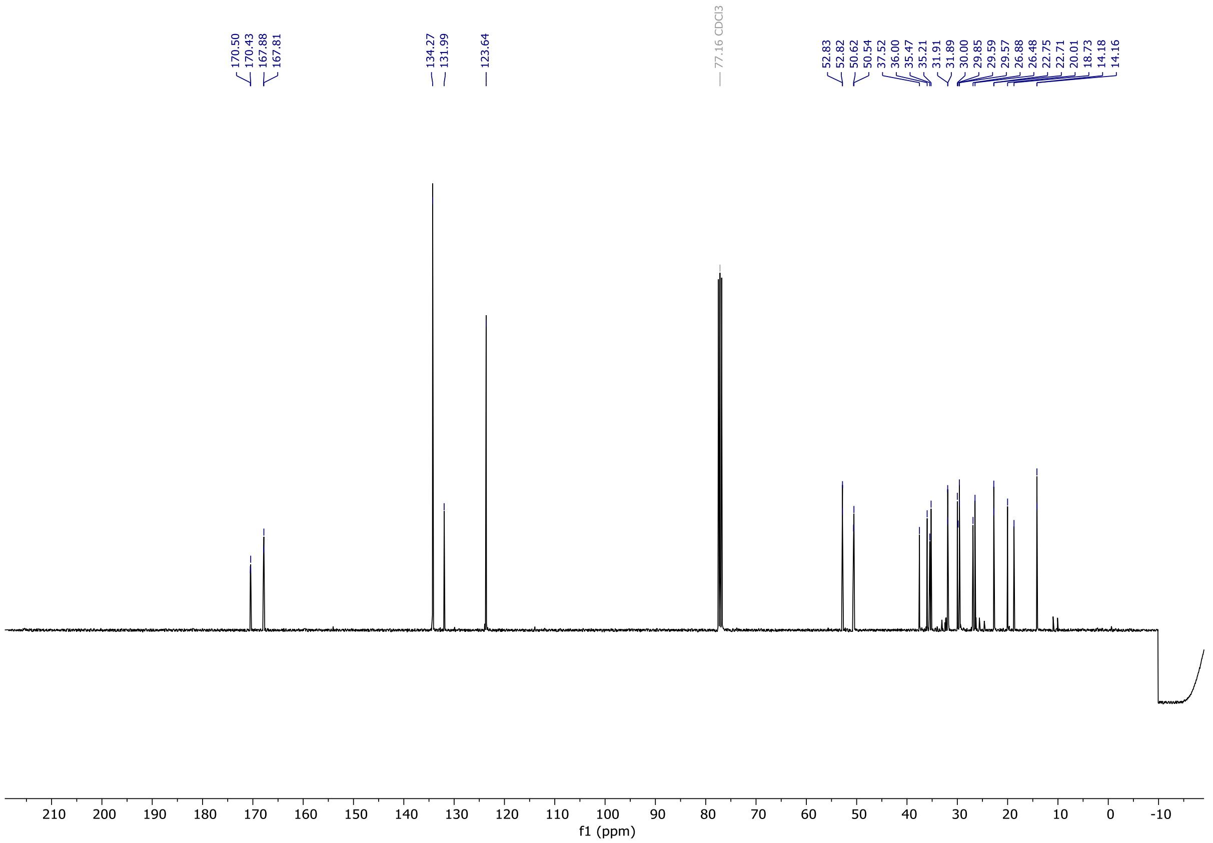

14, ^{1}H NMR (600 MHz, $CDCl_3$)

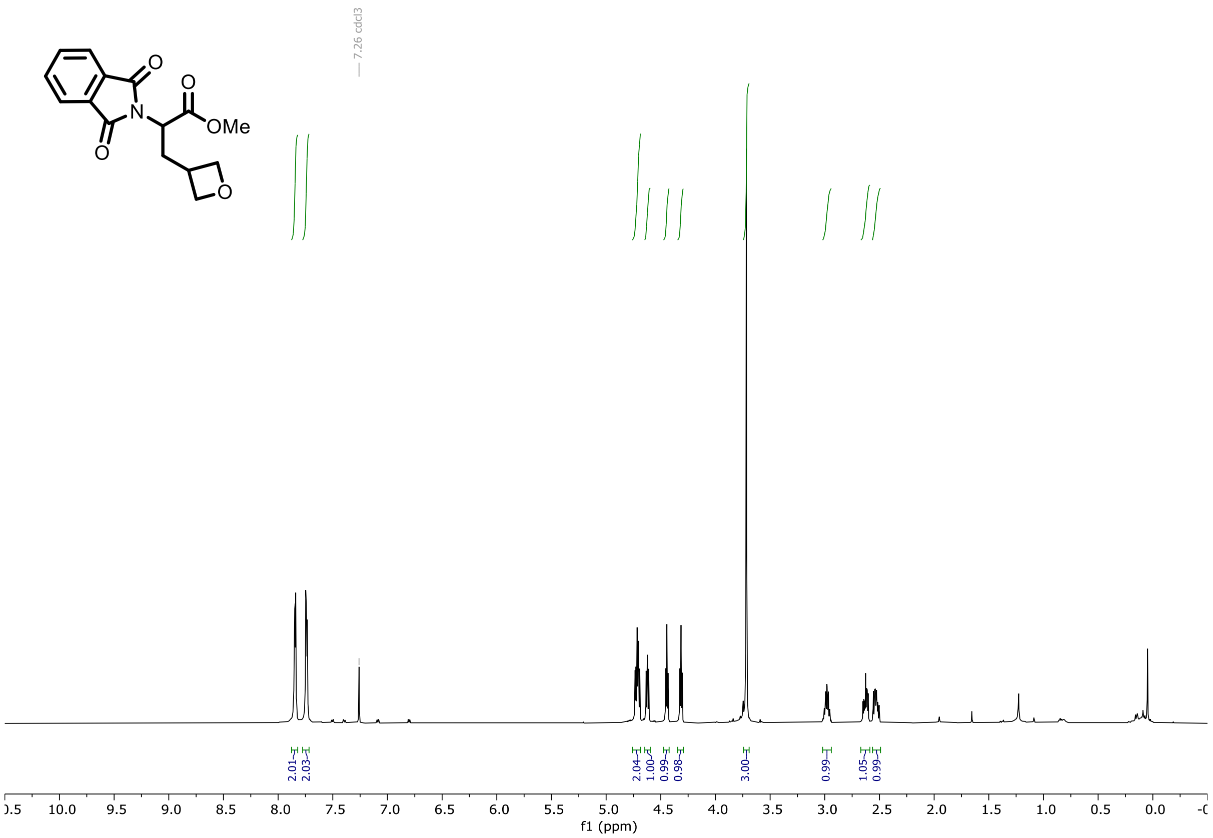

14, ^{13}C NMR (151 MHz, $CDCl_3$)

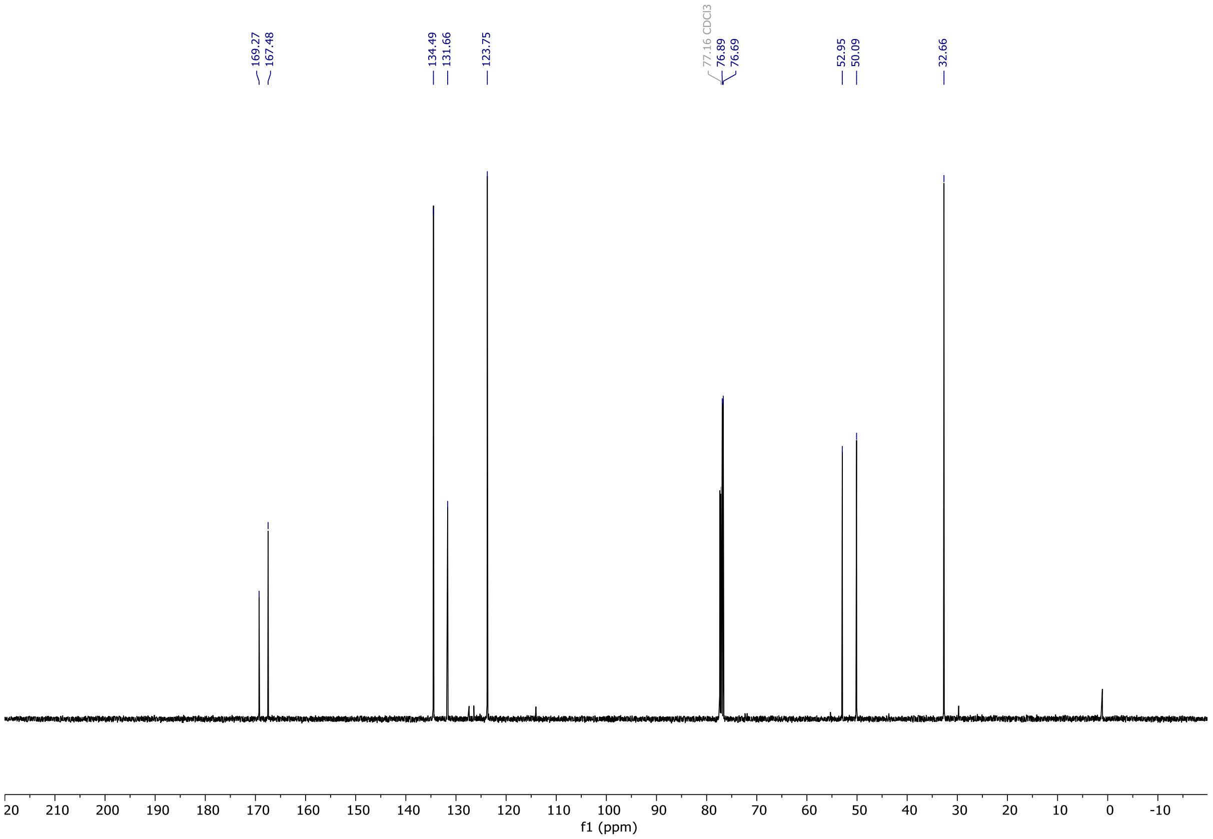

15, ^{1}H NMR (600 MHz, $CDCl_3$)

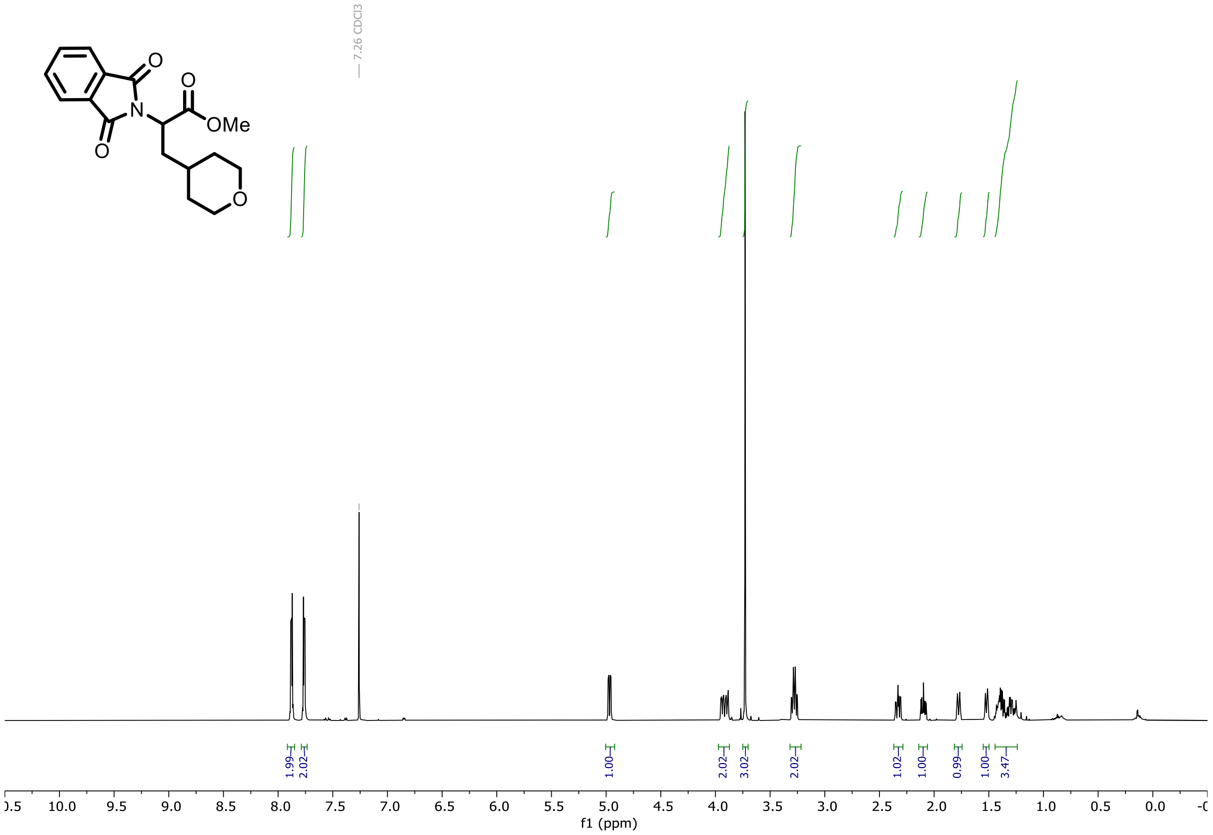

15, ^{13}C NMR (151 MHz, $CDCl_3$)

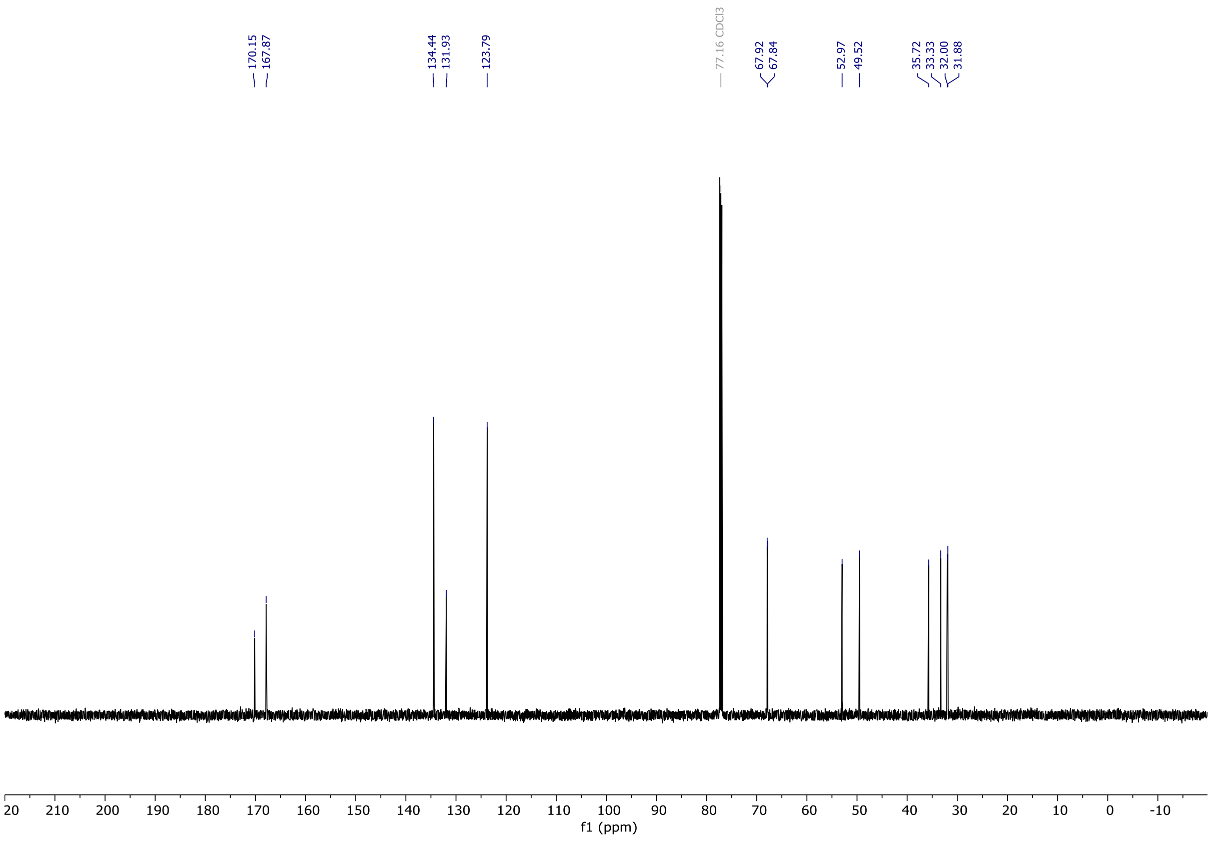

16, ^{1}H NMR (600 MHz, CDCl$_3$)

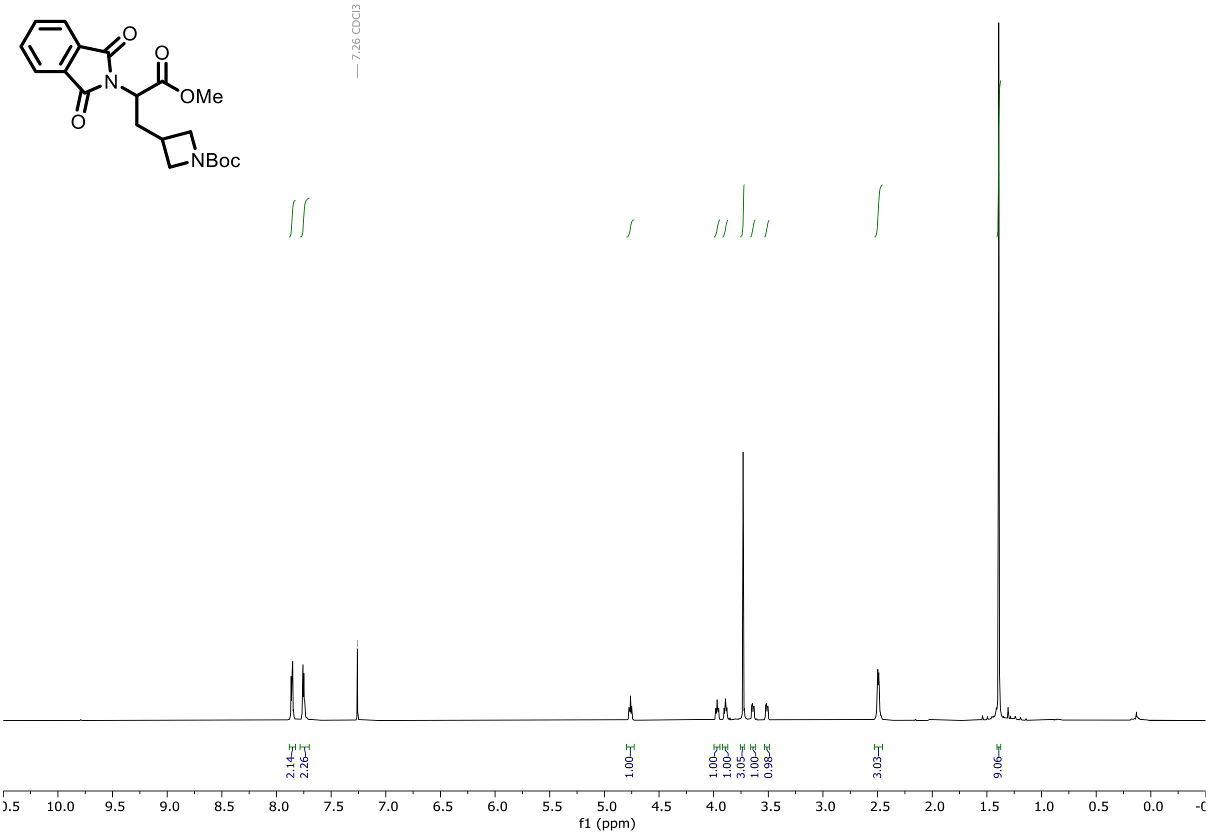

16, ^{13}C NMR (151 MHz, CDCl$_3$)

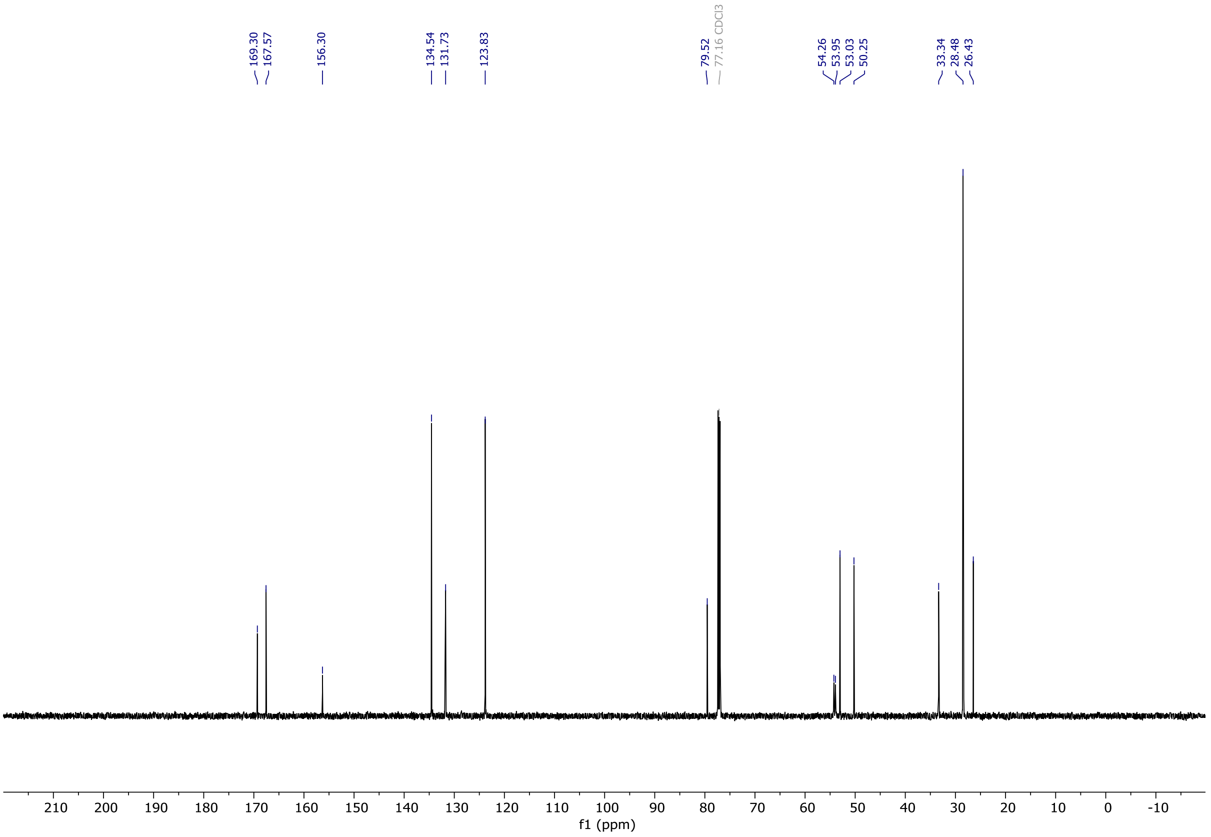

17, ^{1}H NMR (600 MHz, $CDCl_3$)

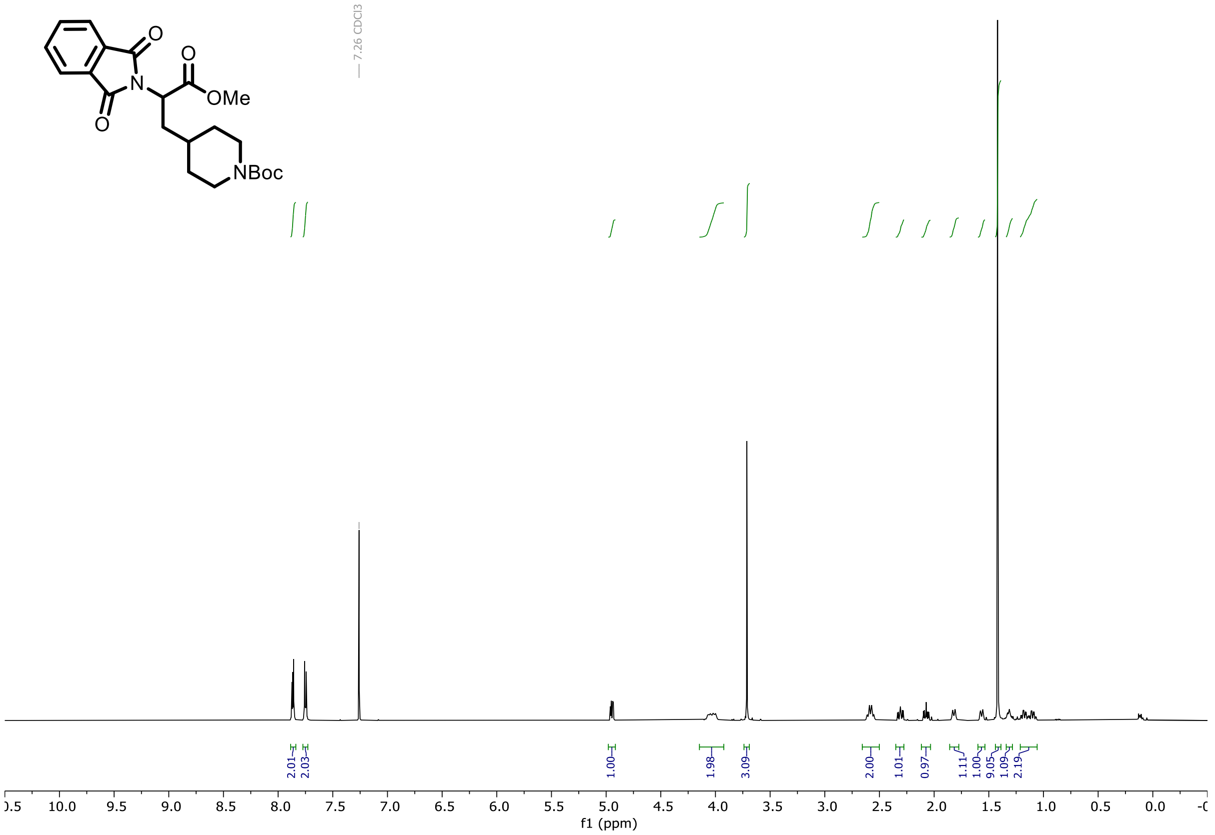

17, ^{13}C NMR (151 MHz, $CDCl_3$)

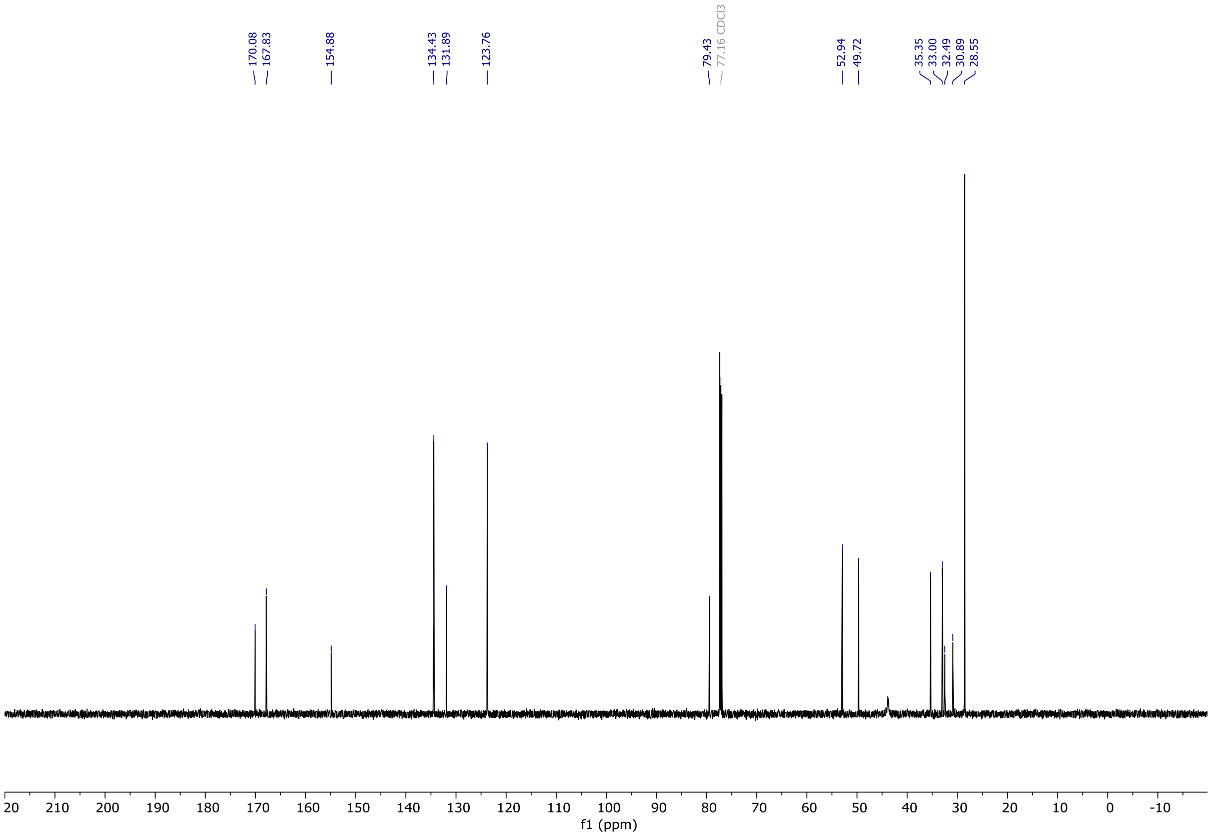

18, ^{1}H NMR (400 MHz, $CDCl_3$)

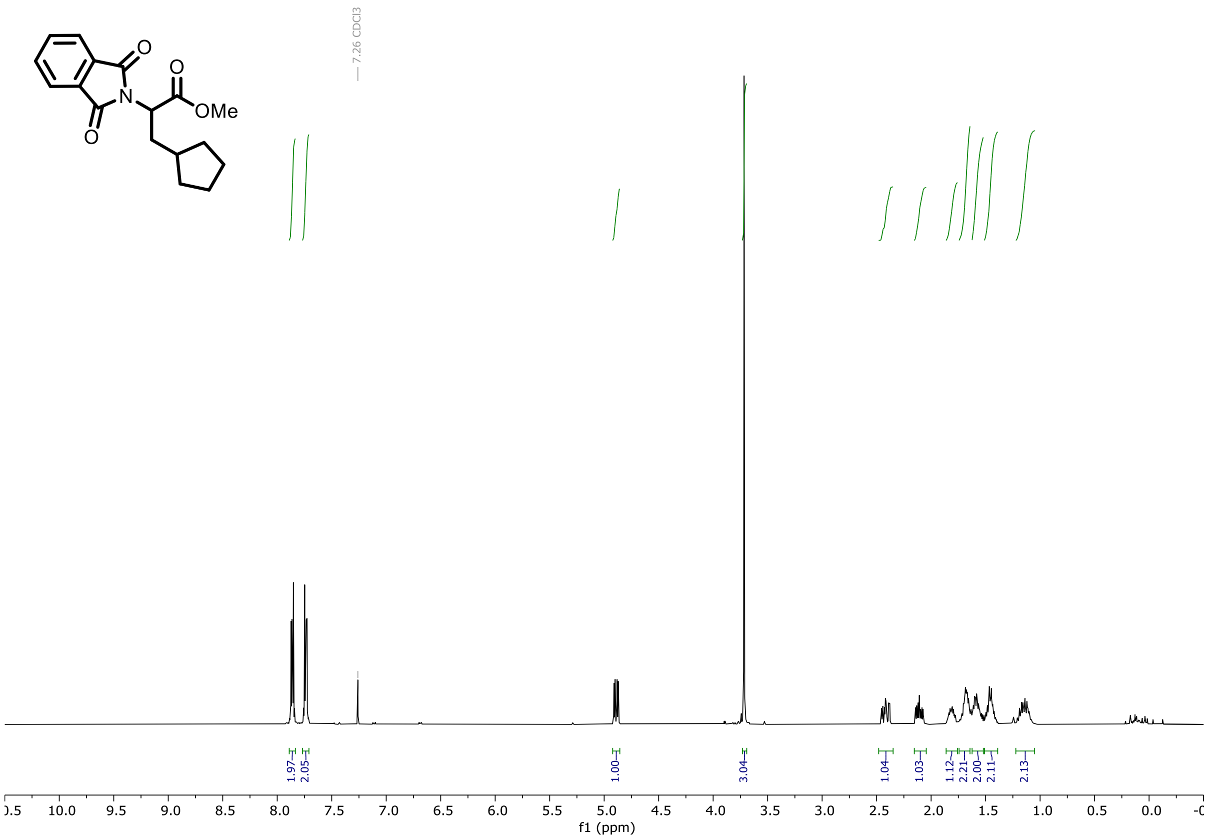

18, ^{13}C NMR (101 MHz, $CDCl_3$)

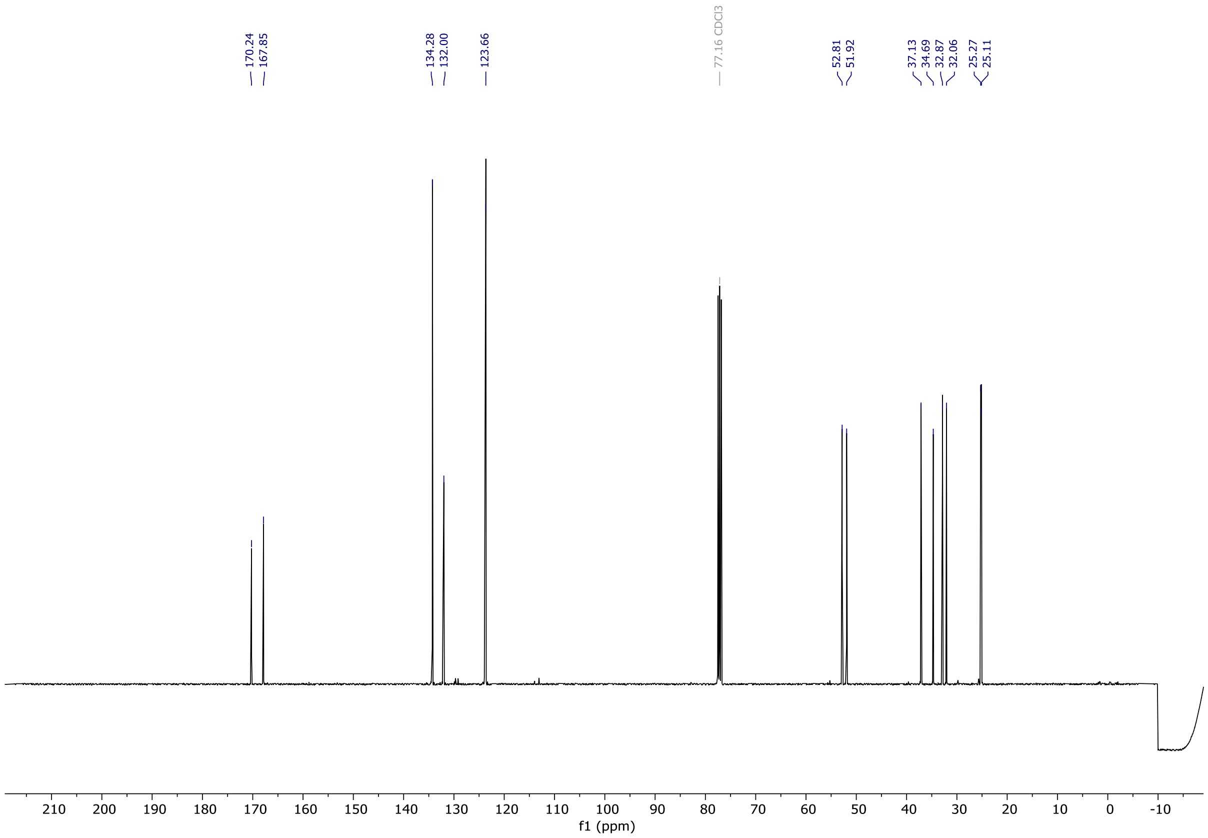

19, ^{1}H NMR (600 MHz, $CDCl_3$)

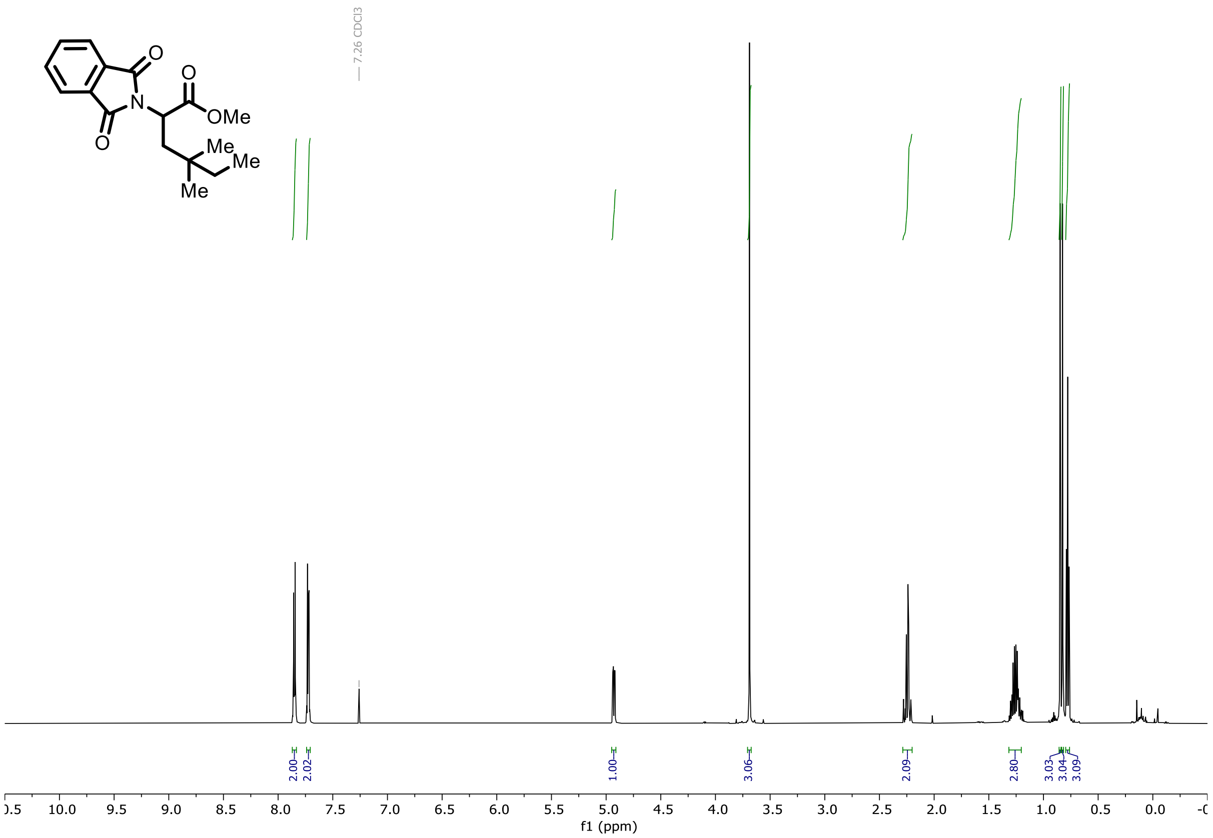

19, ^{13}C NMR (151 MHz, $CDCl_3$)

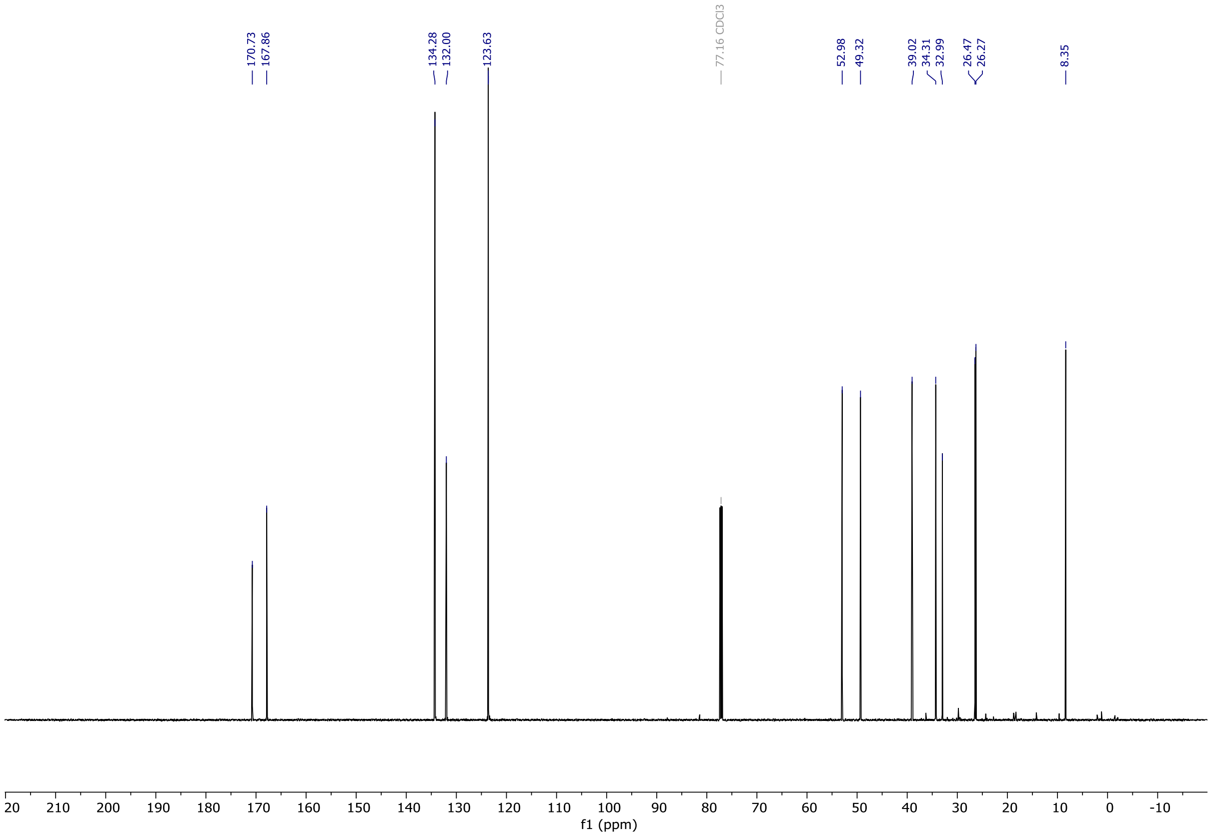

20, ^{1}H NMR (400 MHz, $CDCl_3$)

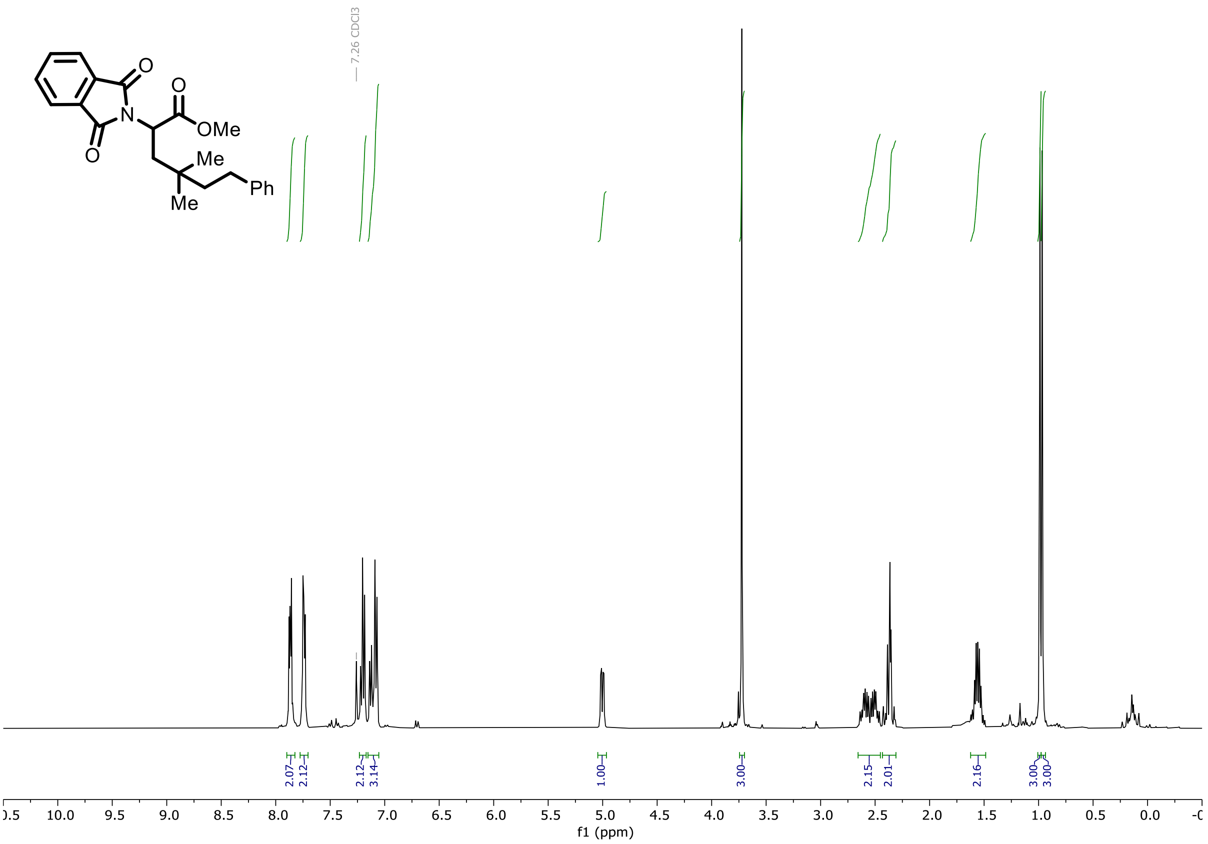

20, ^{13}C NMR (101 MHz, $CDCl_3$)

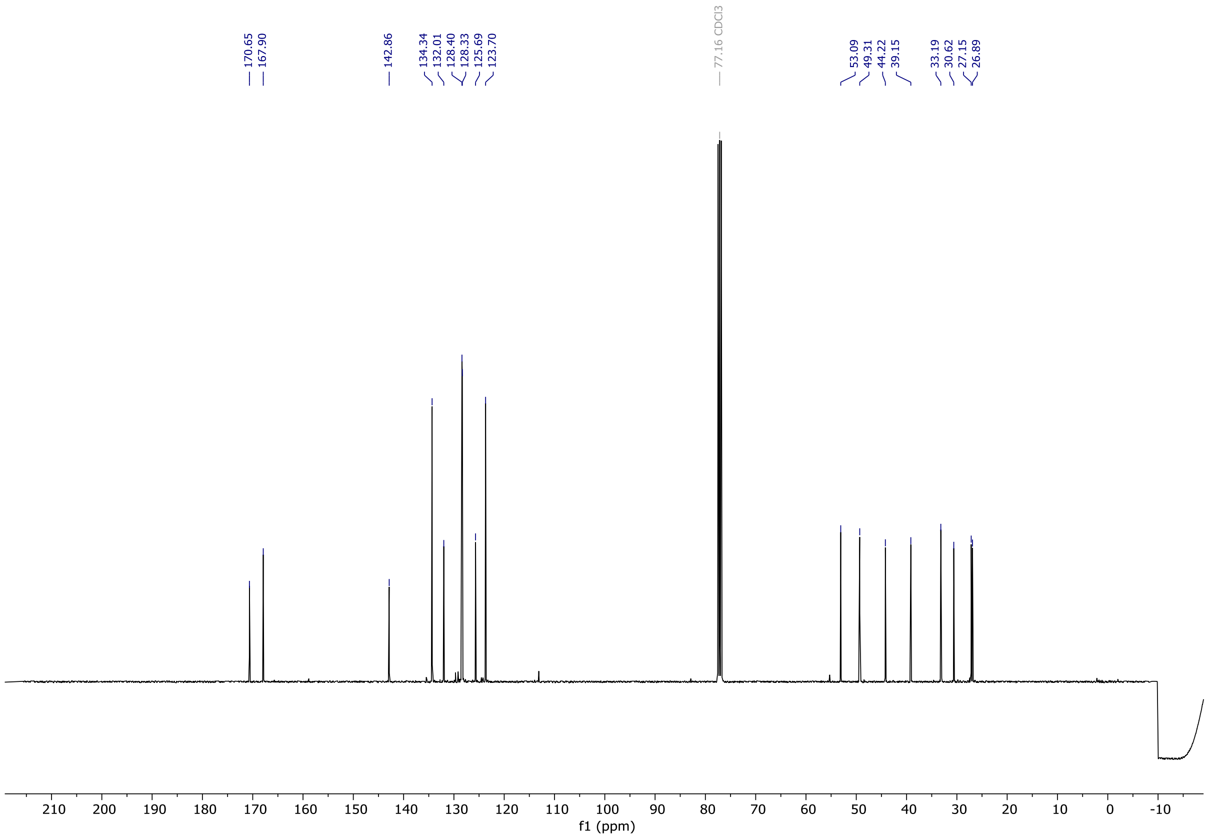

21, ^{1}H NMR (600 MHz, $CDCl_3$)

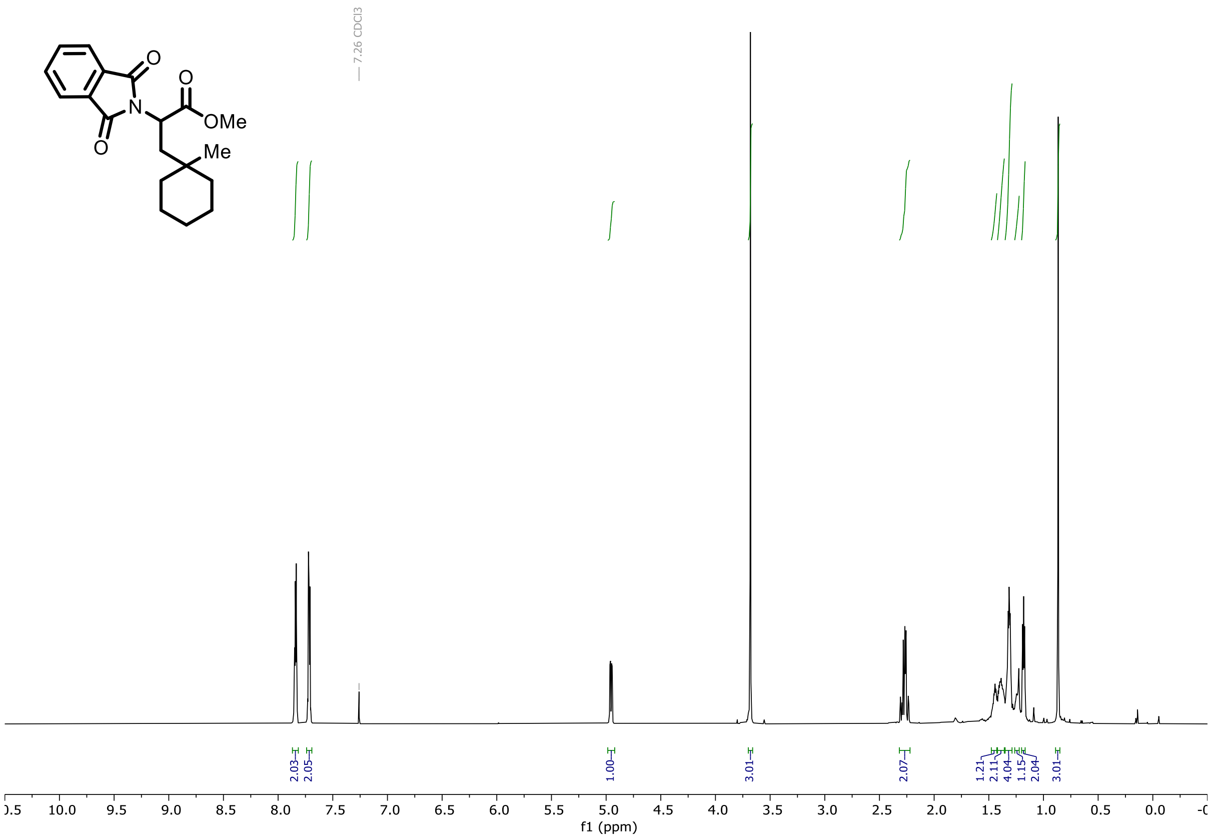

21, ^{13}C NMR (151 MHz, $CDCl_3$)

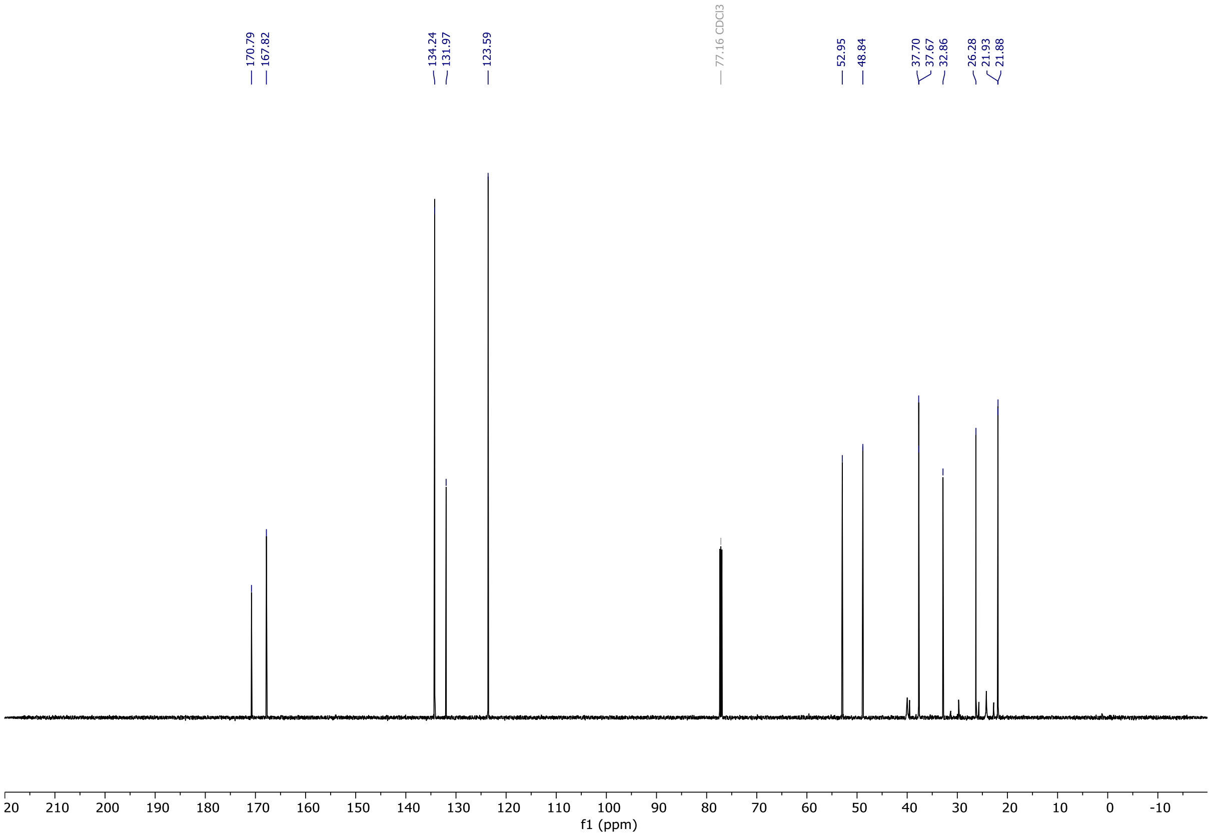

22, ^{1}H NMR (600 MHz, $CDCl_3$)

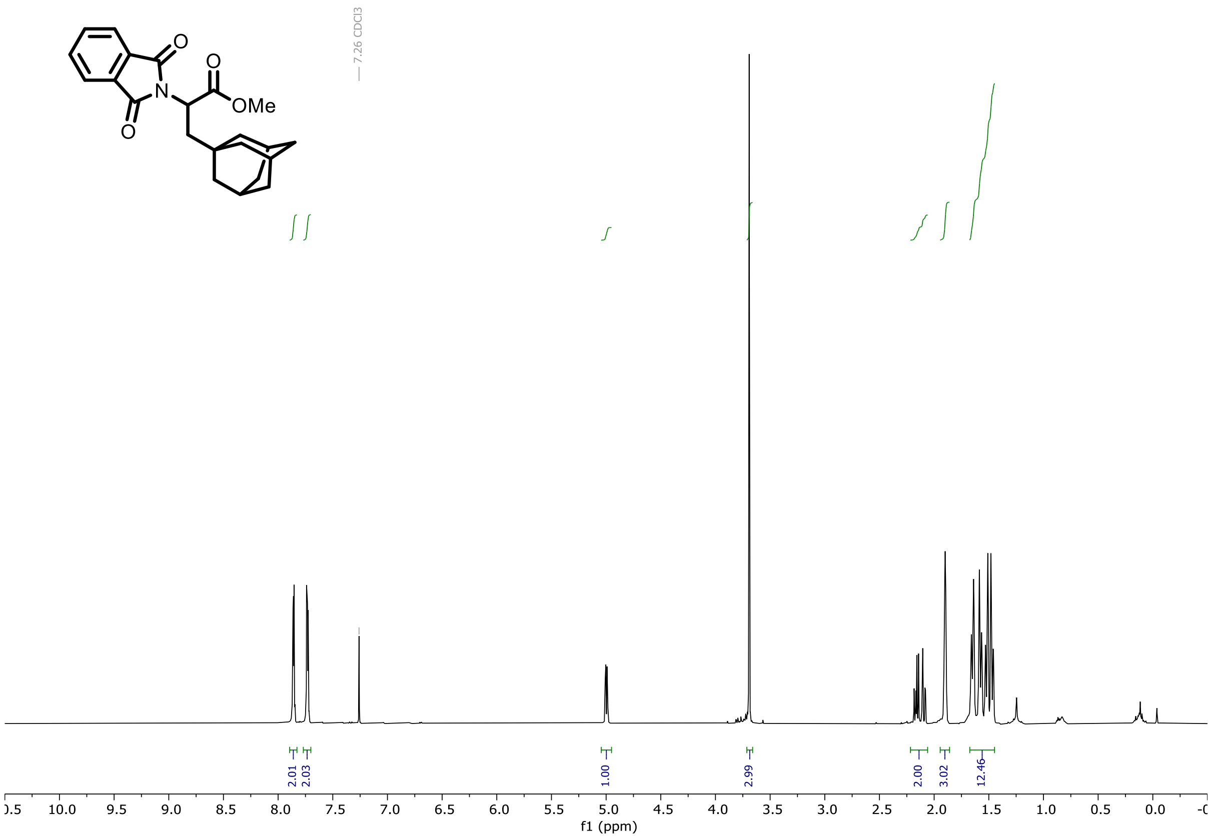

22, ^{13}C NMR (151 MHz, $CDCl_3$)

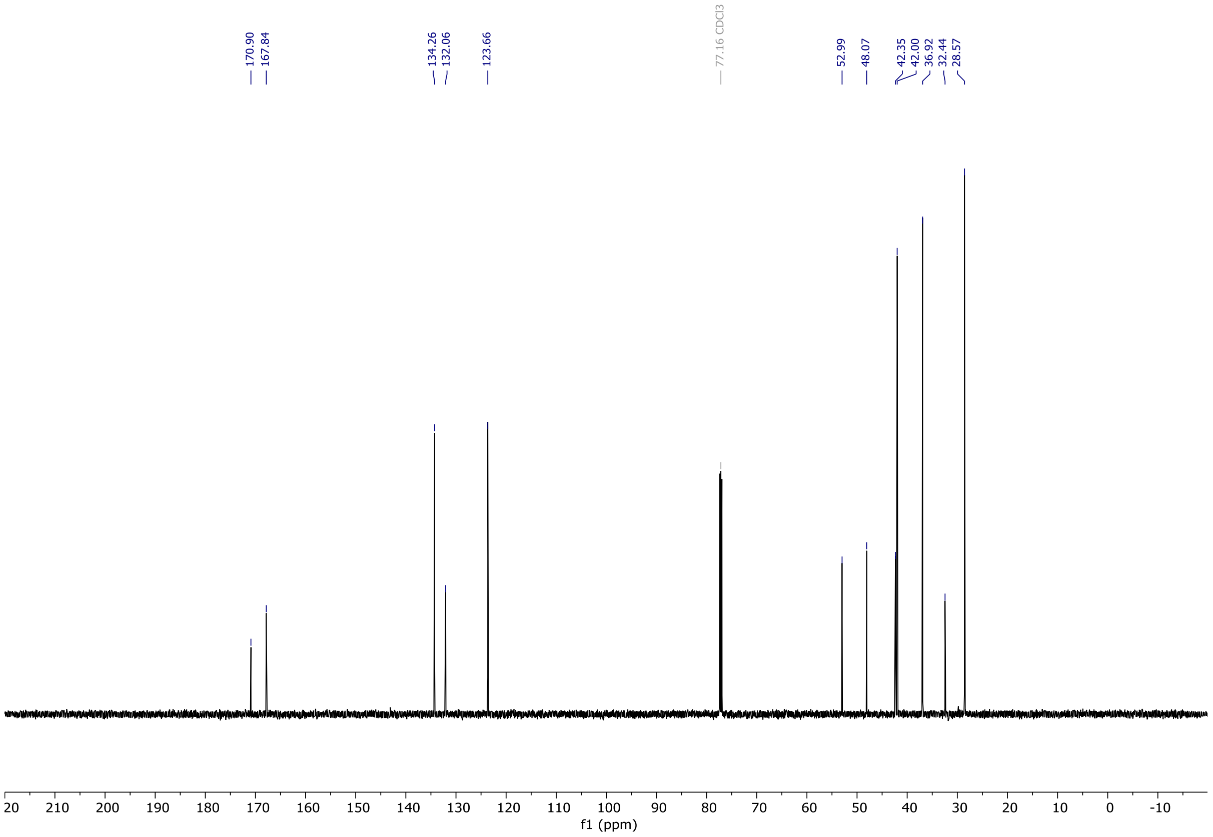

23, ^{1}H NMR (600 MHz, $CDCl_3$)

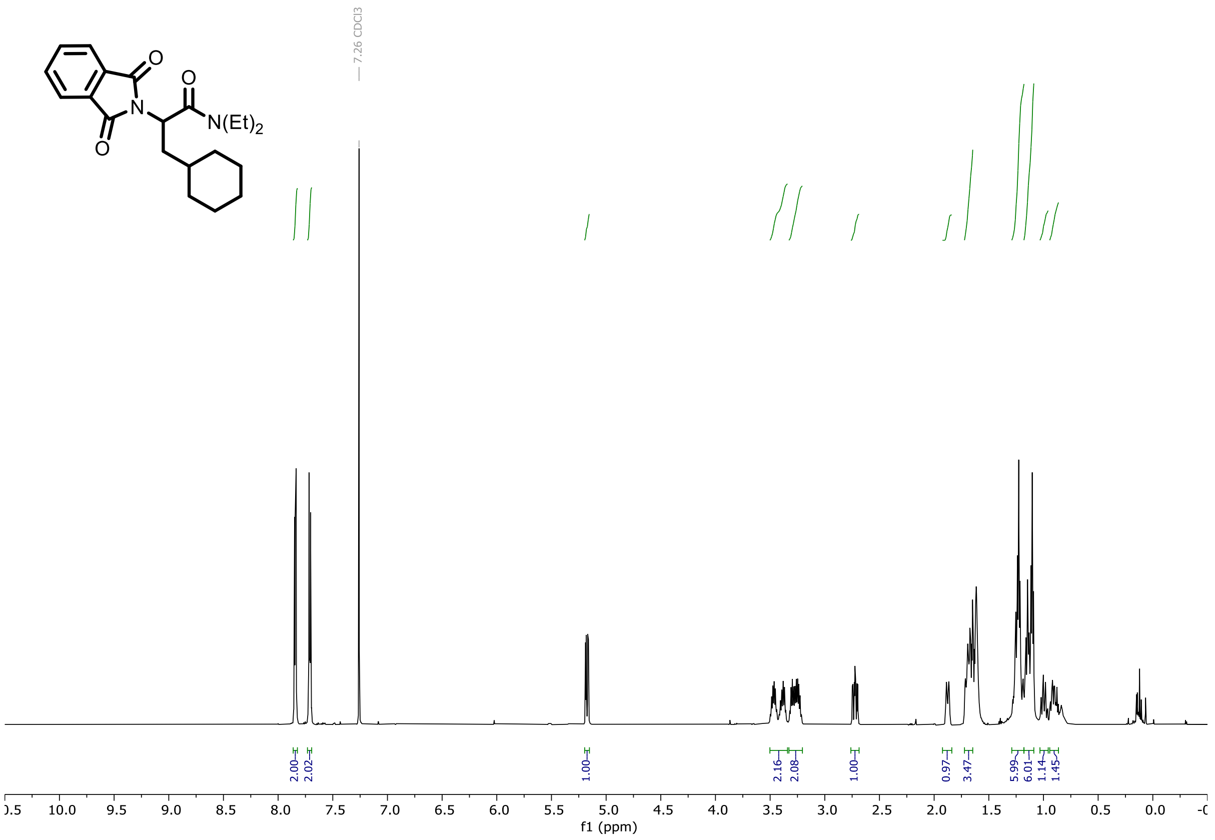

23, ^{13}C NMR (151 MHz, $CDCl_3$)

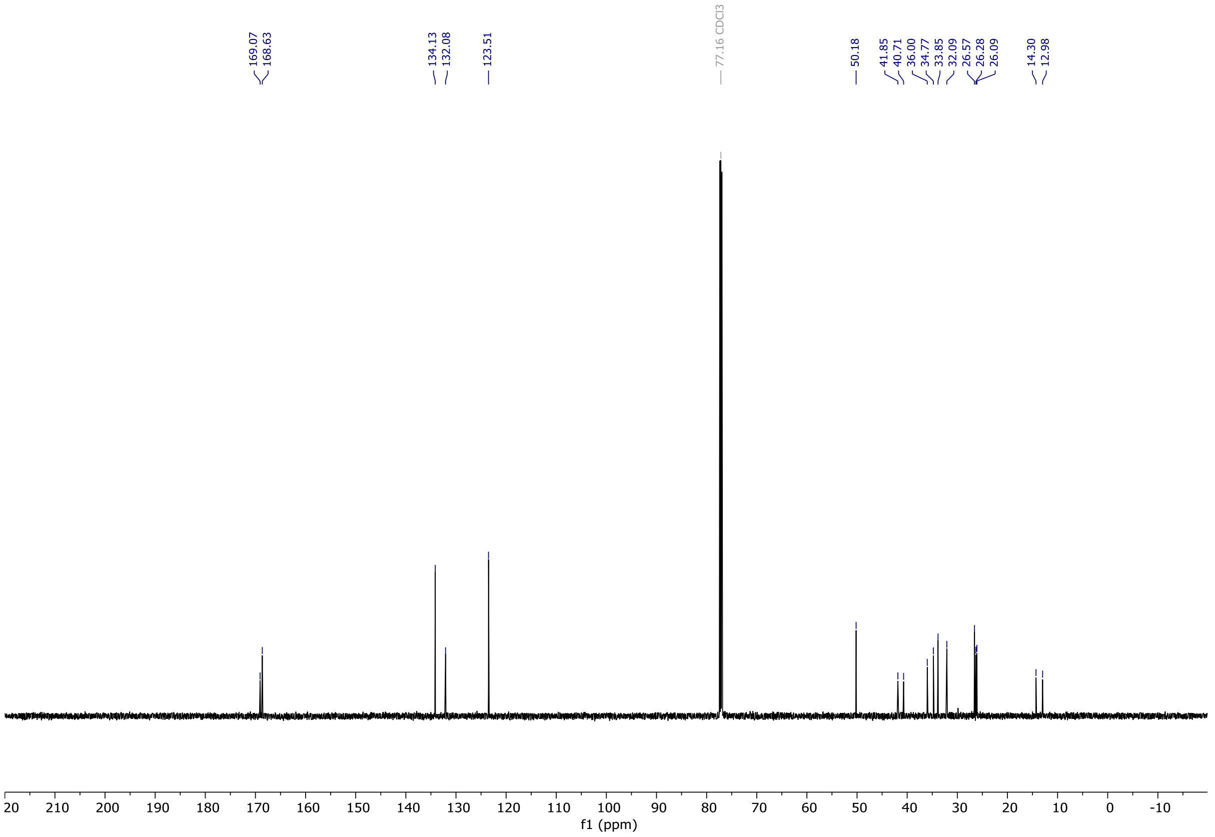

24, ^{1}H NMR (600 MHz, CDCl$_3$)

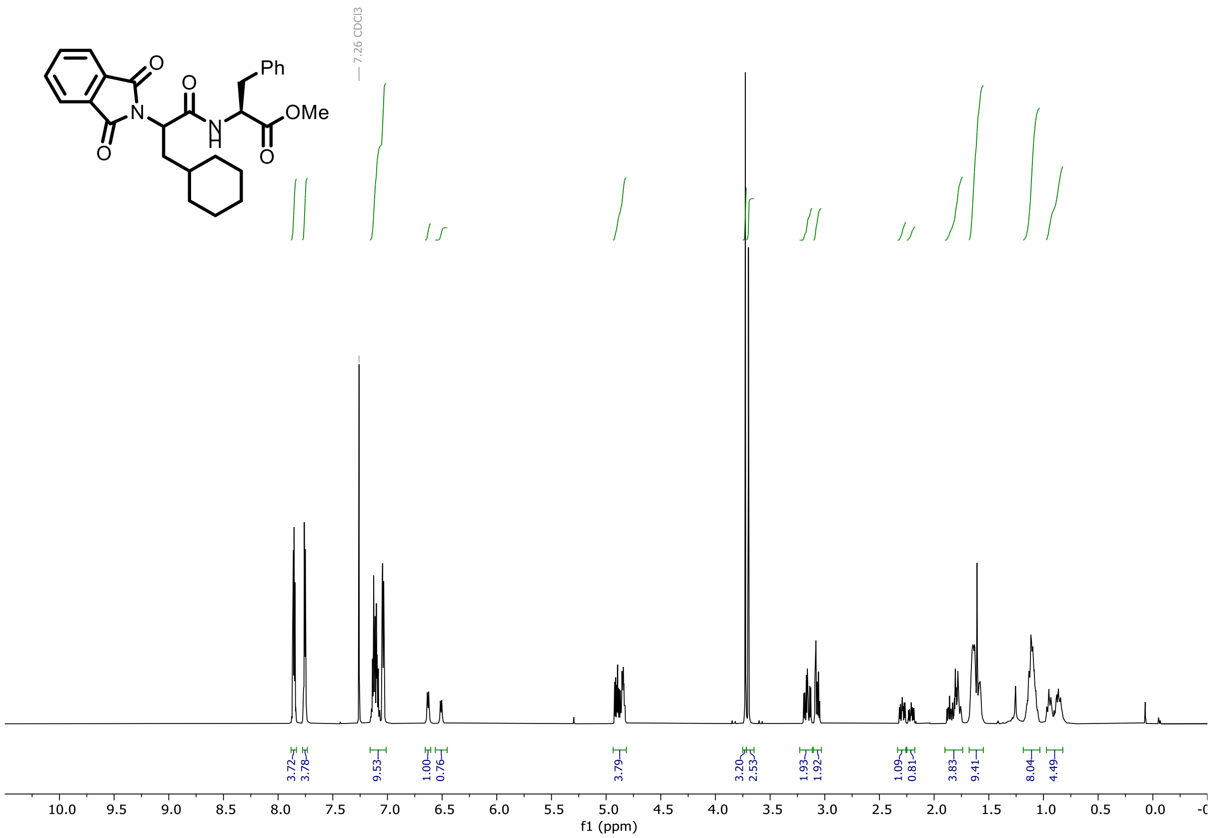

24, ^{13}C NMR (151 MHz, CDCl$_3$)

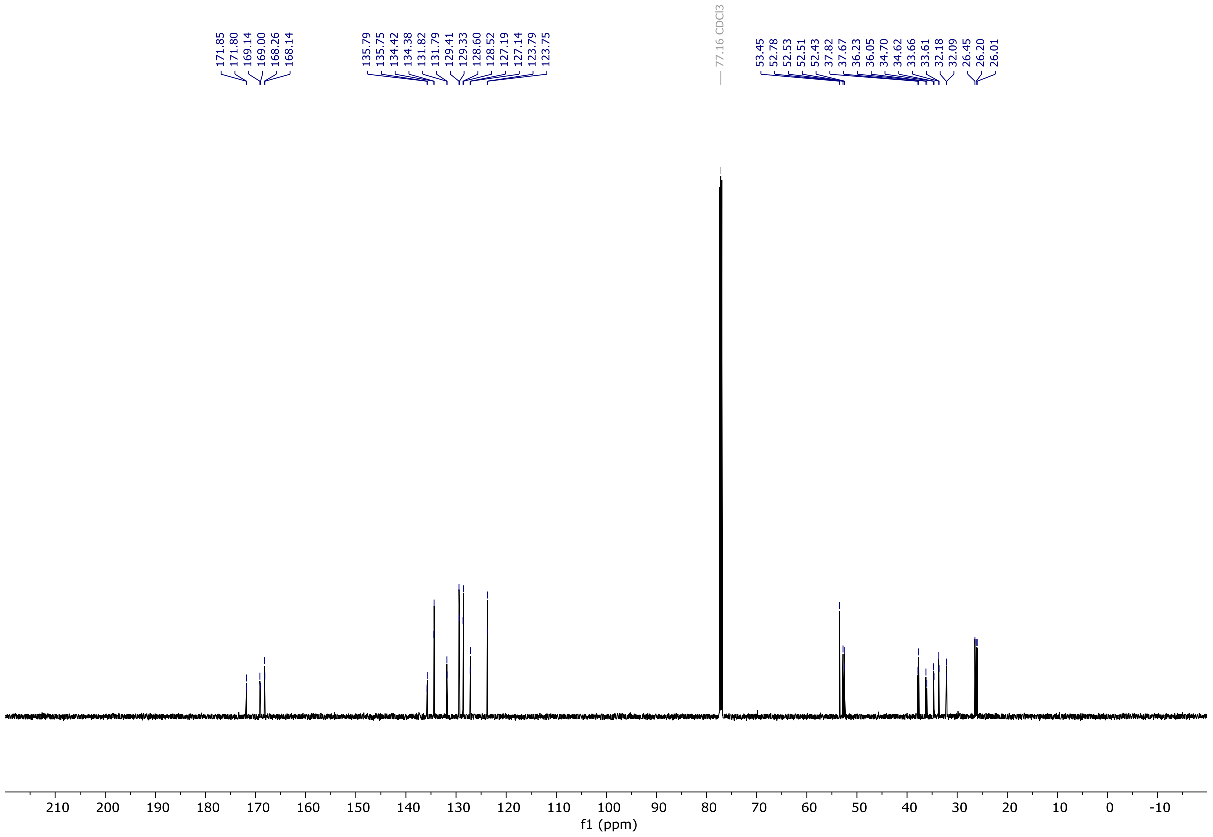

25, ^{1}H NMR (600 MHz, $CDCl_3$)

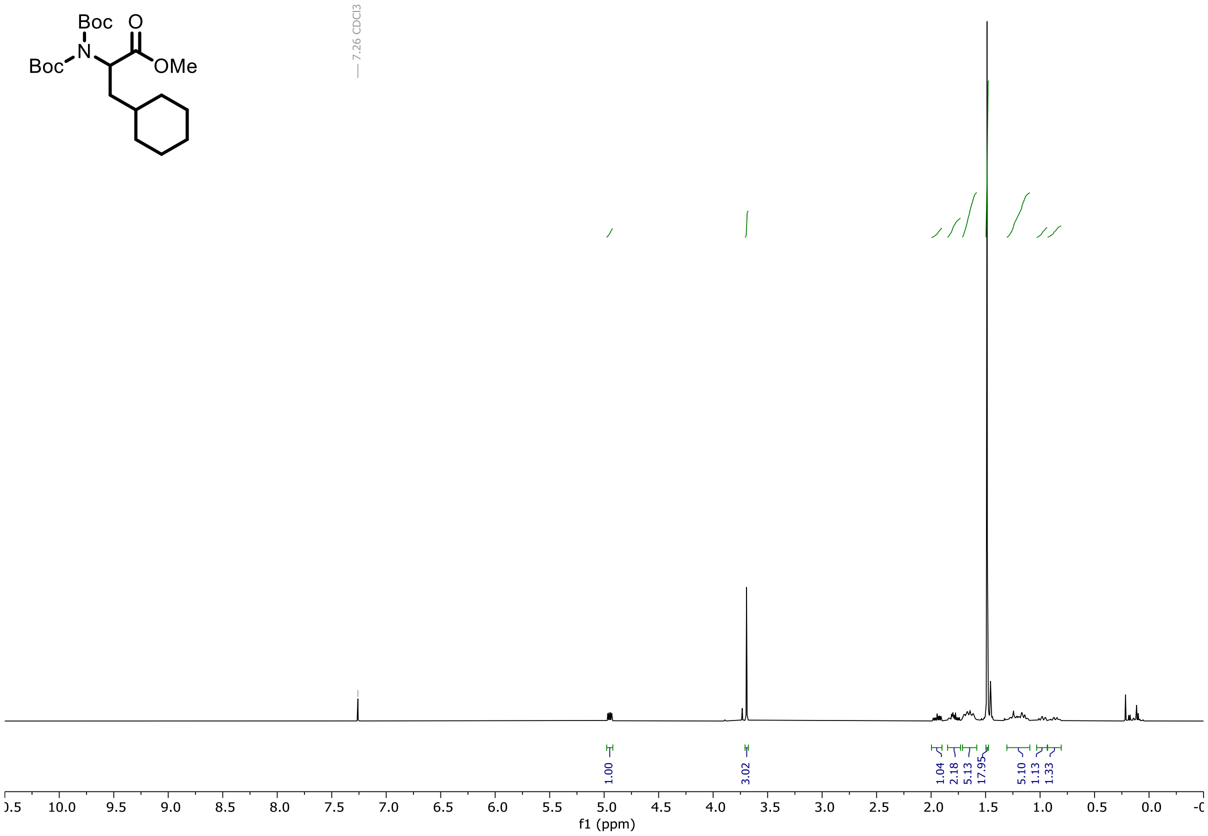

25, ^{13}C NMR (101 MHz, $CDCl_3$)

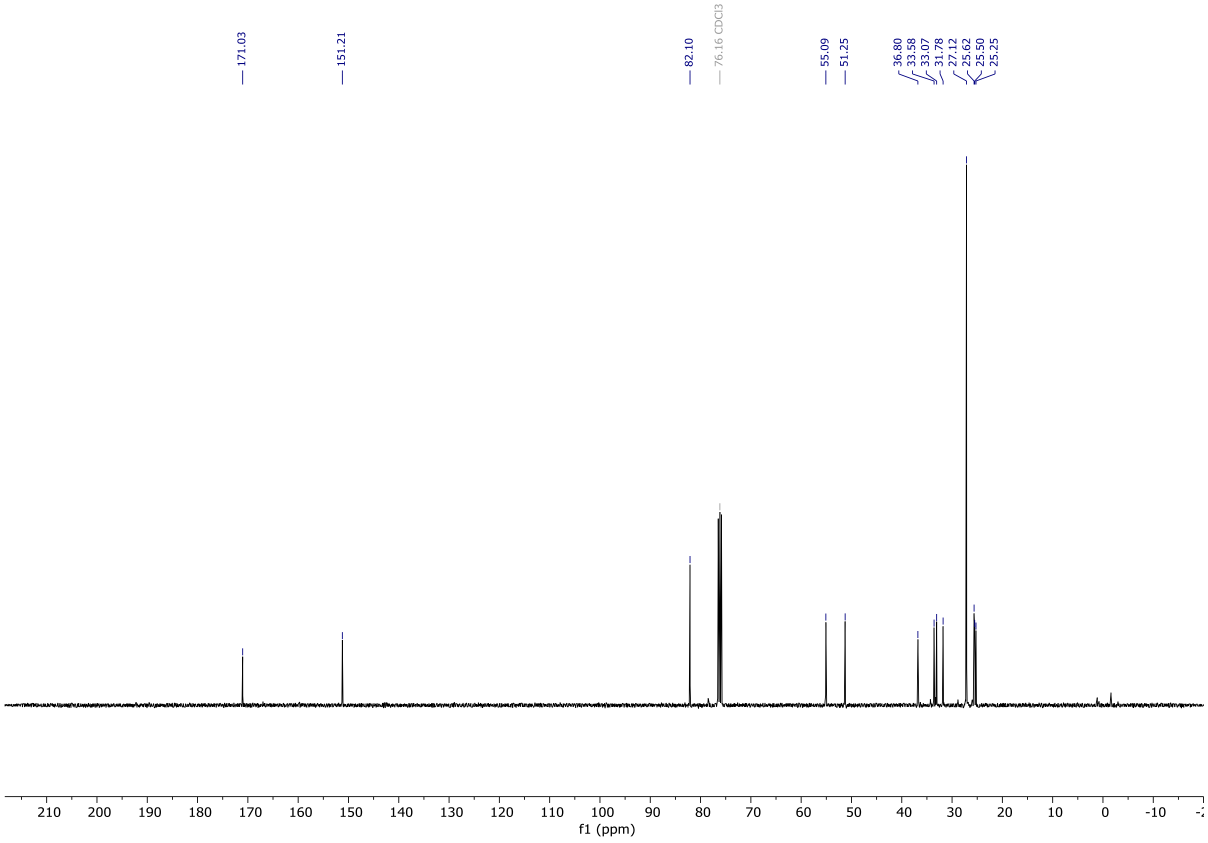

26, ^{1}H NMR (400 MHz, $CDCl_3$)

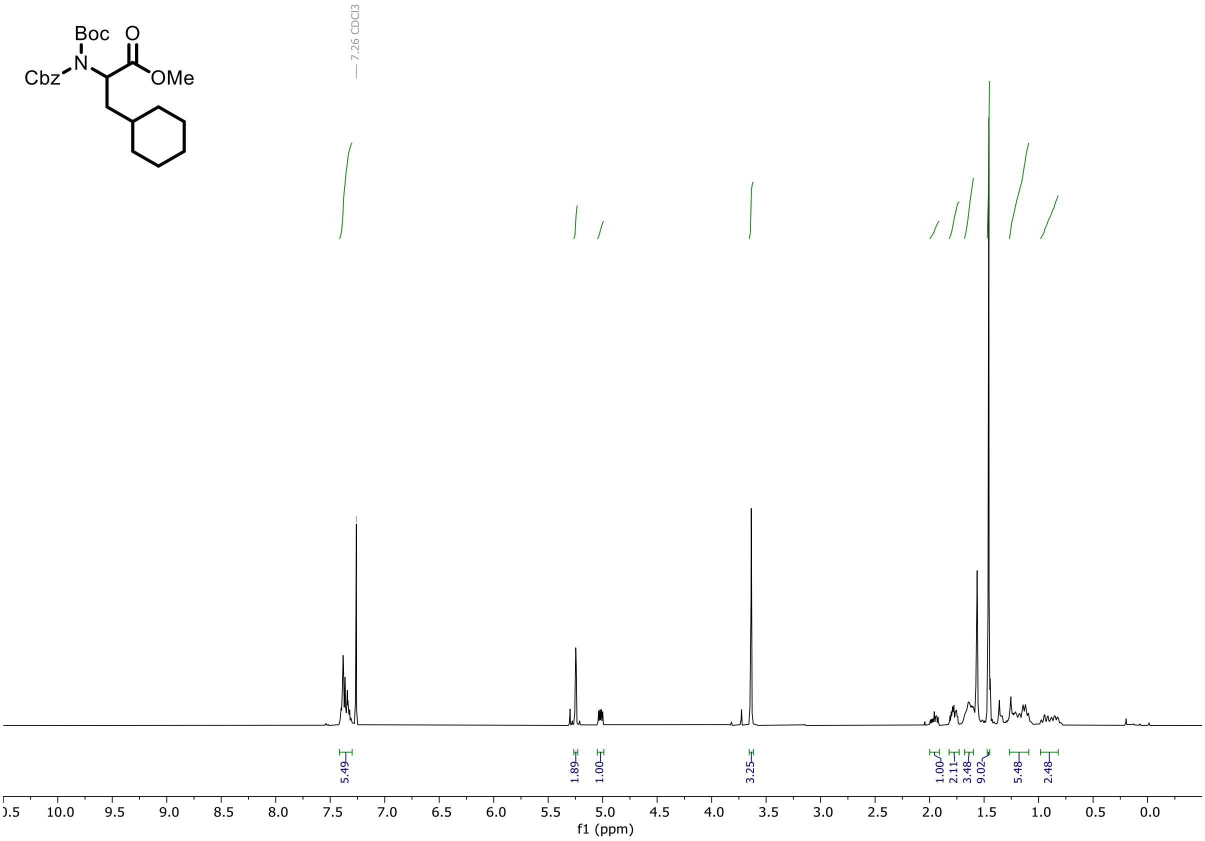

26, ^{13}C NMR (101 MHz, $CDCl_3$)

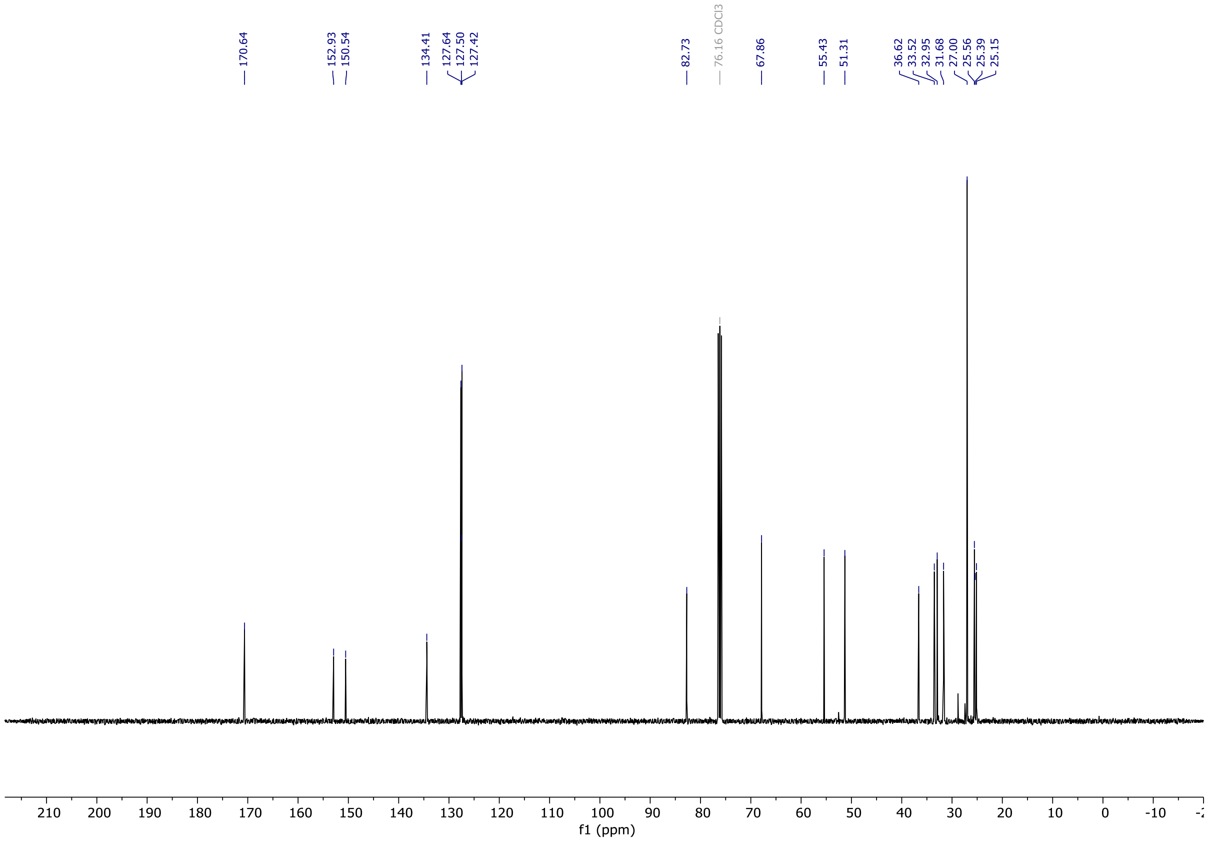

27, ^{1}H NMR (400 MHz, $CDCl_3$)

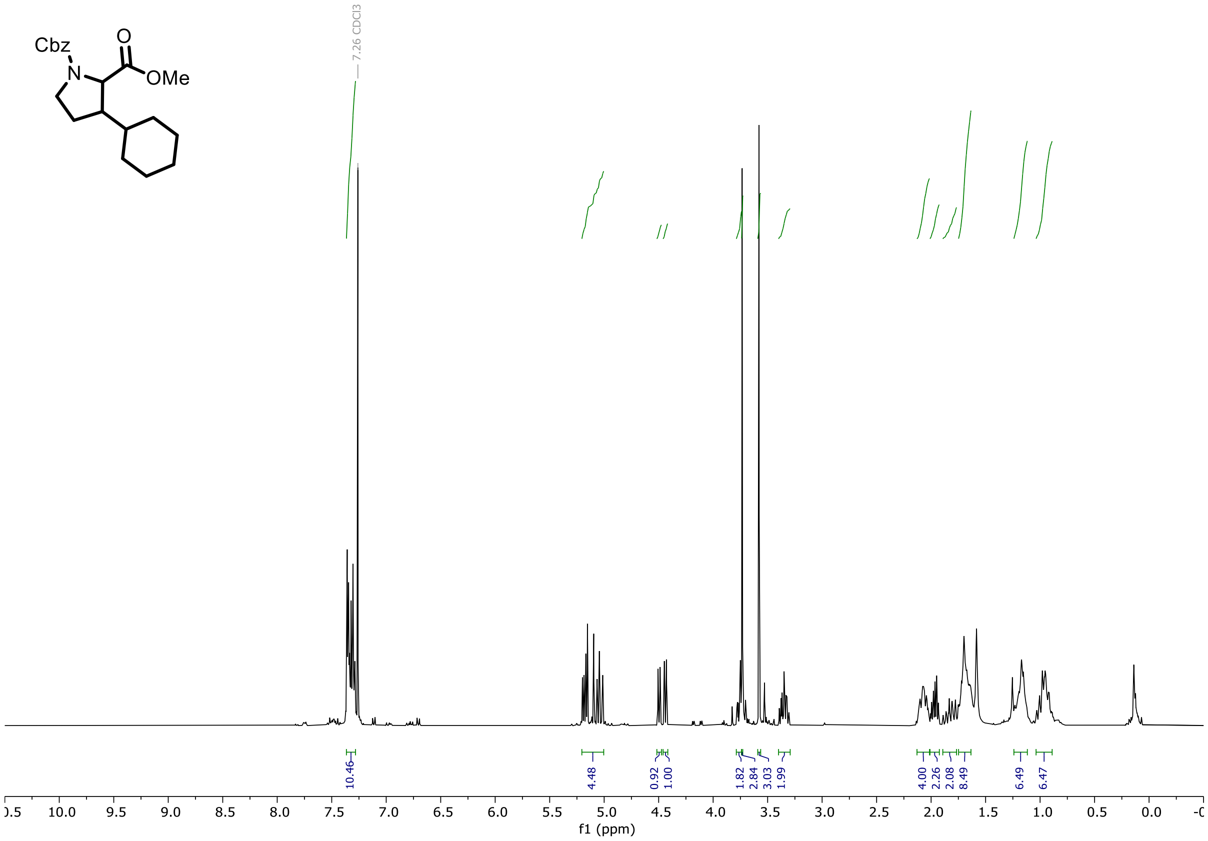

27, ^{13}C NMR (101 MHz, $CDCl_3$)

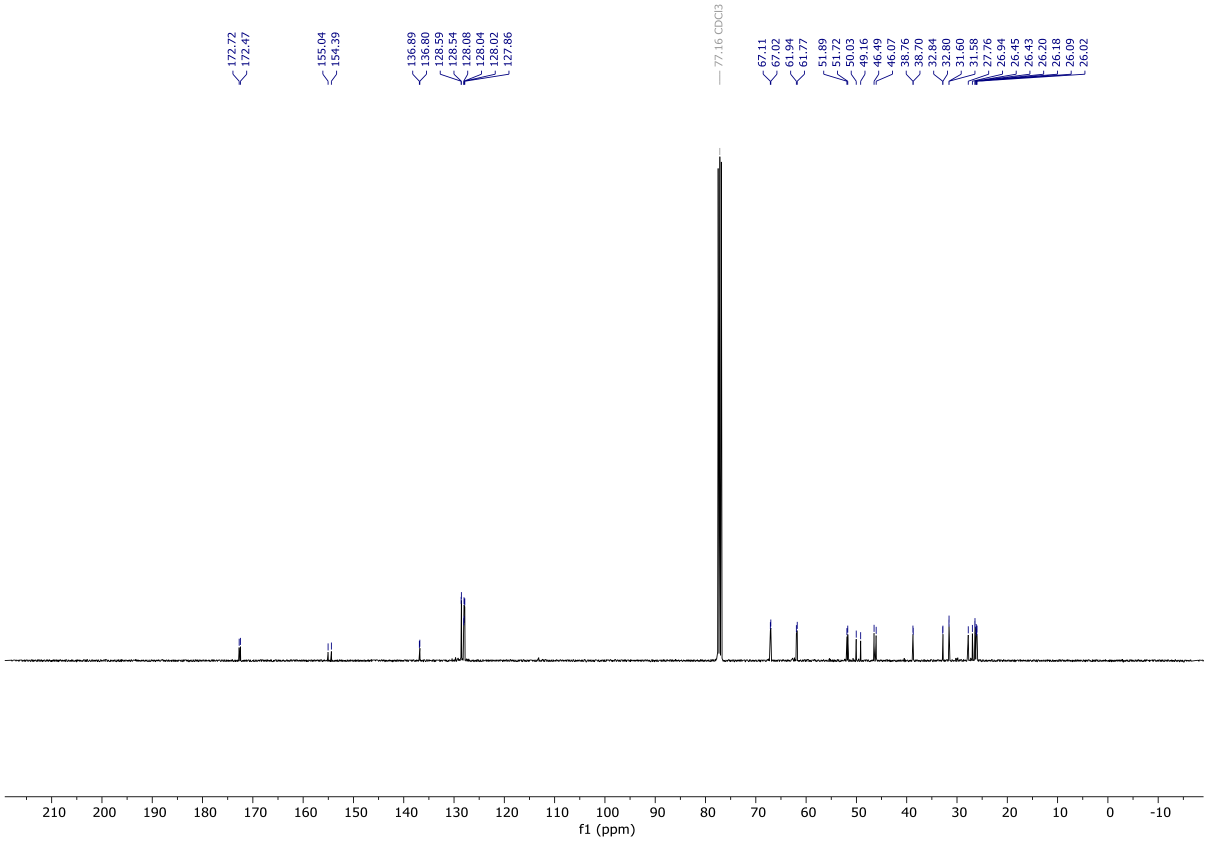

7. References

[1] Wan, Y.; Zhu, J.; Yuan, Q.; Wang, W.; Zhang, Y. *Org. Lett.* **2021**, *23*, 1406−1410.
[2] Aycock, R. A.; Vogt, D. B.; Jui, N. T. C*hem. Sci.* **2017**, *8*, 7998−8003.
[3] Knowles, O. J.; Johannissen, L. O.; Crisenza, G. E. M.; Hay, S.; Leys, D.; Procter, D. J. *Angew. Chem. Int. Ed.* **2022**, *61*, e202212158.
[4] Huy, P.; Neudörfl, J.-M.; Schmalz, H.-G. *Org. Lett.* **2011**, *13*, 216−219.
[5] Sandoval, B.A.; Clayman, P.D.; Oblinsky, D.G.; Oh, S.; Nakano, Y.; Bird, M.; Scholes, G.D.; Hyster, T.K. *J. Am. Chem. Soc.* **2020**, *143*(4), 1735-1739.
[6] Sigmaaldrich. Phosphate buffered saline. https://www.sigmaaldrich.com/NL/en/product/sigma/p4417 (accessed on April 14, 2024)
[7] Ohmatsu, K.; Suzuki, R.; Fujita, H.; Ooi, T. *J. Org. Chem.* **2023**, *88*, 6553–6556
[8] Uraguchi, D.; Kinoshita, N.; Ooi, T. *J. Am. Chem. Soc.* **2010**, *132*, 12240– 12242.
[9] Bower, J. F.; Jumnah, R.; Williams, A. C.; Williams, J. M. J. *J. Chem. Soc., Perkin Trans. 1* **1997**, 1411−1420.
[10] Zhao, J.; Zhang, J.; Fang, P.; Wu, J.; Wang, F.; Liu, Z. Q. *Green Chem.* **2024**, *26*, 507–512.
[11] Nathanael, J. G.; Wille, U. J. *Org. Chem.* **2019**, *84*, 3405–3418.
